# Supplementary material for: Understanding how junction resistances impact the conduction mechanism in nano-networks
Source: Nat Commun. 2024 May 28;15:4517. doi: 10.1038/s41467-024-48614-5 (PMC11133347; doi:10.1038/s41467-024-48614-5)
Supplement: Supplementary file 1 — Supplementary Information [file 41467_2024_48614_MOESM1_ESM.pdf]

Supplementary Information for

Understanding how junction resistances impact the conduction mechanism in nano-networks

Cian Gabbett[1†], Adam G. Kelly[1,2†], Emmet Coleman[1], Luke Doolan[1], Tian Carey[1], Kevin Synnatschke[1], Shixin Liu[1], Anthony Dawson[1], Domhnall O'Suilleabhain[1], Jose Munuera[1,3], Eoin Caffrey[1], John B. Boland[1], Zdeněk Sofer[4], Goutam Ghosh[5], Sachin Kinge[6], Laurens D.A. Siebbeles[5], Neelam Yadav[7], Jagdish K. Vij[7], Muhammad Awais Aslam[8], Aleksandar Matkovic[8] and Jonathan N. Coleman[1]*

[1]School of Physics, CRANN & AMBER Research Centres, Trinity College Dublin, Dublin 2, Ireland.

[2]i3N/CENIMAT, Faculty of Science and Technology, Universidade NOVA de Lisboa, Campus de Caparica, 2829-516 Caparica, Portugal.

[3]Department of Physics, Faculty of Sciences, University of Oviedo, C/ Leopoldo Calvo Sotelo, 18, 33007 Oviedo, Asturias, Spain.

[4]Department of Inorganic Chemistry, University of Chemistry and Technology Prague, Technická 5, Prague 6, 166 28, Czech Republic.

[5]Chemical Engineering Department, Delft University of Technology, Van der Maasweg 9, NL-2629 HZ Delft, The Netherlands.

[6]Materials Research & Development, Toyota Motor Europe, B1930 Zaventem, Belgium.

[7]Department of Electronic & Electrical Engineering, Trinity College Dublin 2, Ireland.

[8]Chair of Physics, Department Physics, Mechanics and Electrical Engineering, Montanuniversität Leoben, Franz Josef Strasse 18, 8700 Leoben, Austria.

[†]These authors contributed equally

*colemaj@tcd.ie (Jonathan N. Coleman); Tel: +353 (0) 1 8963859.

Contents

Table of Abbreviations 3
Supplementary note 1. Model development 5
Supplementary note 2. Nanosheet and nanowire size analysis 22
Supplementary note 3. Fitting DC resistivity data 33
Supplementary note 4. Impedance basics 36
Supplementary note 5. Conversion of Z_{Net} to $Z_{NS\text{-}J}$: Mathematics 38
Supplementary note 6. Conversion of Z_{Net} to $Z_{NS\text{-}J}$: Concepts and an example 40
Supplementary note 7. Transistor measurements on a nanosheet network 43
Supplementary note 8. Measuring impedance: Substrate capacitance 44
Supplementary note 9. Measuring impedance: Influence of substrate capacitance 45
Supplementary note 10. Measuring impedance: Background artefacts 47
Supplementary note 11. Measuring impedance: Contact resistance 49
Supplementary note 12. Impedance: Fitting to equivalent circuits 51
Supplementary note 13. Impedance: Fitting methodologies 57
Supplementary note 14. Room-temperature impedance of MoS_2 59
Supplementary note 15. Extracting nanosheet resistivity directly from the network impedance 61
Supplementary note 16. Terahertz spectroscopy 62
Supplementary note 17. Transistor measurements on an individual nanosheet 65
Supplementary note 18. Impedance of networks of 2D materials beyond MoS_2 66
Supplementary note 19. Fitting temperature-dependent impedance of MoS_2 67
Supplementary note 20. Measuring the inter-sheet junction area 70
Supplementary note 21. The effective permittivity of an MoS_2 network 71
Supplementary note 22. Temperature-dependent transport in nanosheet networks 73
References 79

Table of Abbreviations

R_J .... Interparticle Junction Resistance

R_{NP} .... Nanoparticle Resistance

R_{NS} .... Nanosheet Resistance

R_{NW} .... Nanowire Resistance

ρ_{NS} .... Nanosheet Resistivity

ρ_{NW} .... Nanowire Resistivity

μ_{NP} .... Nanoparticle Mobility

μ_{NS} .... Nanosheet Mobility

μ_{NW} .... Nanowire Mobility

n_{NP} .... Nanoparticle Carrier Density

n_{NS} .... Nanosheet Carrier Density

n_{NW} .... Nanowire Carrier Density

ρ_{Net} .... Network Resistivity

P_{Net} .... Network Porosity

μ_{Net} .... Network Mobility

σ_{Net} .... Network Conductivity

n_{Net} .... Network Carrier Density

l_{NP} .... Nanoparticle Length

A_{NP} .... Nanoparticle Cross-sectional Area

e .... Elementary Charge

l_{NW} .... Nanowire Length

D_{NW} .... Nanowire Diameter

A_{NW} .... Nanowire Cross-sectional Area

t_{NS} .... Nanosheet Thickness

l_{NS} .... Nanosheet Length

k_{NS} .... Nanosheet Aspect Ratio

Z_{Net} .... Network Impedance

$Z_{NS\text{-}J}$ .... Impedance of an Average Nanosheet-Junction Pair

ρ^*_{Net} .... Complex Network Resistivity

Re(ρ^{*}_{Net}).........................Real Component of Complex Network Resistivity

Re(Z_{NS-J}).........................Real Component of the Impedance of a Nanosheet-Junction Pair

Im(Z_{NS-J}).........................Imaginary Component of the Impedance of a Nanosheet-Junction Pair

Re(Z_{NS-J}).........................Real Component of the Impedance of a Nanosheet-Junction Pair

Im(Z_{NS-J}).........................Imaginary Component of the Impedance of a Nanosheet-Junction Pair

C_J....................................Junction Capacitance

n.....................................Impedance ideality Factor

T ....Temperature

E_a.....................................Activation Energy

k_B....................................Boltzmann Constant

A_J....................................Junction Area

l_J.......................................Junction (Inter-sheet) Separation

m.....................................Effective Electron Mass

m_e ....Electron Mass

d_0.....................................Monolayer Thickness

A_{Net} ....Channel Cross-sectional Area

L_{Ch} or $L_{Channel}$.................Channel Length

W_{Ch} or $W_{Channel}$...............Channel Width

a.....................................Localisation Length

Supplementary note 1. Model development

Here we develop a simple model describing the conductivity and mobility of networks of 1D (nanotubes, nanowires etc.), 2D (nanosheets, nanoplatelets etc.), or 0D (nanodots etc.) nano-particles as a function of both particle (e.g. size) and network (e.g. porosity, junction resistance, tortuosity) properties. We note that we expect these models to apply to networks that are thick enough that the disorder associated with local thickness fluctuations (often refered to as percolation effects)[1] is not dominant. Such films are in the bulk-like regime and demonstrate thickness-independent conductivity[1, 2].

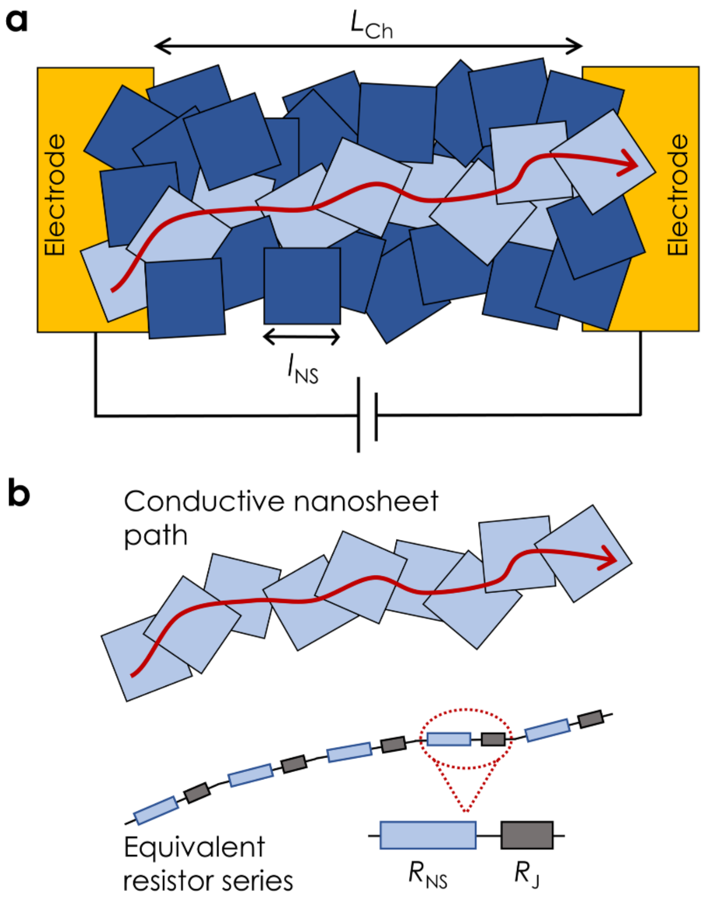

Supplementary figure 1: Model schematic for a nanosheet network. (a) Schematic illustrating a nanosheet network connected to two electrodes with channel length, L_{Ch}, under an applied voltage. A single conducting path consisting of a linear array of nanosheets is shown spanning the channel length (red arrow). The nanosheet lateral size is l_{NS}. While this schematic depicts a nanosheet network, a similar diagram could easily be produced to represent a nanowire network. **(b)** This conducting path can be considered as a chain of resistor pairs, with each pair consisting of a resistance representing a nanosheet, R_{NS}, and the inter-sheet junction, R_J.

1.1 Calculation of network mobility using a random-resistor model

We begin by discussing the general case of networks of nanoparticles. Here, by nanoparticles we mean those with any dimension on the nanometer scale, for example 2D nanosheets, 1D nanowires or 0D nanodots. The basic principle of this model is that we consider charge carriers flowing through the network via a large number of parallel current paths. Here we consider a single (average) current path. The basic unit of a current path is the nanoparticle/junction pair (Suppl. Fig. 1). When moving along a current path, a carrier travels a distance L from one electrode to another. This is greater than the electrode-to-electrode channel length (L_{Ch}) because the conductive path is not straight and is related to the channel length via the tortuosity factor[3], κ_{Net}, such that $L = L_{\mathrm{Ch}}\sqrt{\kappa_{\mathrm{Net}}}$. We include the tortuosity factor in our calculations here for completeness. However, in many networks, we expect $\kappa_{\mathrm{Net}} \approx 1$ so we neglect it in the main manuscript.

Every time an electron passes through a nanoparticle, it must also pass through a junction to get to the next nanoparticle. A current path is made up of many of these pairs in series. Here, the average resistances experienced by an electron moving through individual nanoparticles and junctions are R_{NP} and R_{J}. When a voltage, V, is applied to a network, we can consider its effect on a current path. In a given path, the voltage will be dropped over the entire set of nanoparticle/junction pairs. The number of pairs in a conductive path is $N = L/(d_{NP} + d_J)$ where d_{NP} is the distance the electron travels on average through a single nanoparticle before jumping to the next nanoparticle and the forward distance travelled during the inter-particle jump is d_{J} (where we expect $d_{\mathrm{J}} << d_{\mathrm{NP}}$). The average voltage drop across one junction-nanoparticle pair is

$$V_{\mathrm{p}} = V/N = V \times (d_{\mathrm{NP}} + d_{\mathrm{J}})/L$$

This voltage drop can then be separated into separate contributions due to the junction and the nanoparticle, $V_{\mathrm{p}} = V_{\mathrm{J}} + V_{\mathrm{NP}}$. The voltage drop across a junction is given by

$$V_{\mathrm{J}} = \frac{R_{\mathrm{J}}}{R_{\mathrm{J}} + R_{\mathrm{NP}}} V \frac{(d_{\mathrm{NP}} + d_{\mathrm{J}})}{L}$$

And the voltage drop across a nanoparticle is

$$V_{\mathrm{NP}} = \frac{R_{\mathrm{NP}}}{R_{\mathrm{J}} + R_{\mathrm{NP}}} V \frac{(d_{\mathrm{NP}} + d_{\mathrm{J}})}{L}$$

These equations can now be used to calculate some conduction properties. The time taken for an electron to cross the entire network is the channel length, L_{Ch}, divided by the effective carrier speed

$$\tau_{\mathrm{Net}} = \frac{L_{\mathrm{Ch}}}{\mu_{\mathrm{Net}} E_{\mathrm{A}}} = \frac{L_{\mathrm{Ch}} L_{\mathrm{Ch}}}{\mu_{\mathrm{Net}} V}$$

where $E_{\mathrm{A}} = V / L_{\mathrm{Ch}}$ is the applied field. Since a current path has N nanoparticle/junction pairs

$$\tau_{\mathrm{Net}} \approx N(\tau_{\mathrm{NP}} + \tau_{\mathrm{J}}) = \frac{L}{(d_{\mathrm{NP}} + d_{\mathrm{J}})}(\tau_{\mathrm{NP}} + \tau_{\mathrm{J}})$$

where τ_{NP} and τ_{J} are the times taken to cross a nanoparticle and a junction respectively. The time to cross a nanoparticle is given by

$$\tau_{\mathrm{NP}} = \frac{d_{\mathrm{NP}}}{\mu_{\mathrm{NP}} E_{\mathrm{NP}}} = \frac{d_{\mathrm{NP}}{}^2}{\mu_{\mathrm{NP}} V_{\mathrm{NP}}} = \frac{d_{\mathrm{NP}}{}^2}{\mu_{\mathrm{NP}}} \frac{R_{\mathrm{J}} + R_{\mathrm{NP}}}{R_{\mathrm{NP}}} \frac{L}{(d_{\mathrm{NP}} + d_{\mathrm{J}})V}$$

The time to cross the junction can be related to the current flowing across the junction

$$\tau_{\mathrm{J}} = \frac{e}{I_{\mathrm{J}}} = \frac{eR_{\mathrm{J}}}{V_{\mathrm{J}}}$$

Using the above expression for V_J, we find

$$\tau_{\mathrm{J}} = \frac{e}{I_{\mathrm{J}}} = \frac{eR_{\mathrm{J}}}{V_{\mathrm{J}}} = e(R_{\mathrm{J}} + R_{\mathrm{NP}}) \frac{L}{(d_{\mathrm{NP}} + d_{\mathrm{J}})V}$$

Then

$$\tau_{\mathrm{Net}} = \frac{L_{\mathrm{Ch}}^2}{\mu_{\mathrm{Net}} V} \approx \frac{L}{(d_{\mathrm{NP}} + d_{\mathrm{J}})} \left[\frac{d_{\mathrm{NP}}{}^2}{\mu_{\mathrm{NP}}} \frac{R_{\mathrm{J}} + R_{\mathrm{NP}}}{R_{\mathrm{NP}}} \frac{L}{(d_{\mathrm{NP}} + d_{\mathrm{J}})V} + e(R_{\mathrm{J}} + R_{\mathrm{NP}}) \frac{L}{(d_{\mathrm{NP}} + d_{\mathrm{J}})V} \right]$$

Tidying terms then gives (where $\kappa_{\mathrm{Net}} = (L / L_{\mathrm{Ch}})^2$ is the tortuosity factor)

$$\frac{1}{\mu_{\mathrm{Net}}} \approx \kappa_{\mathrm{Net}} \frac{R_{\mathrm{J}} + R_{\mathrm{NP}}}{(d_{\mathrm{NP}} + d_{\mathrm{J}})^2} \left[\frac{d_{\mathrm{NP}}{}^2}{\mu_{\mathrm{NP}} R_{\mathrm{NP}}} + e \right]$$

Or

$$\mu_{\mathrm{Net}} = \frac{(d_{\mathrm{NP}} + d_{\mathrm{J}})^2 / d_{\mathrm{NP}}{}^2}{\left(1 + \frac{R_{\mathrm{J}}}{R_{\mathrm{NP}}}\right)} \frac{\mu_{\mathrm{NP}} / \kappa_{\mathrm{Net}}}{\left[1 + \frac{\mu_{\mathrm{NP}} e R_{\mathrm{NP}}}{d_{\mathrm{NP}}{}^2}\right]}$$

When d_J is very small compared to d_{NP}, this reduces to

$$\mu_{Net} \approx \frac{\mu_{NP}/\kappa_{Net}}{\left(1+\frac{R_J}{R_{NP}}\right)\left[1+\frac{R_{NP}e\mu_{NP}}{d_{NP}^2}\right]} = \frac{\mu_{NP}/\kappa_{Net}}{\left(1+\frac{R_J}{R_{NP}}\right)\left[1+\frac{\sigma_{NP}R_{NP}}{n_{NP}d_{NP}^2}\right]} = \frac{\mu_{NP}/\kappa_{Net}}{\left(1+\frac{R_J}{R_{NP}}\right)\left[1+\frac{d_{NP}/A_{NP}}{n_{NP}d_{NP}^2}\right]}$$

where A_{NP} is the effective nanoparticle cross-sectional area, with $R_{NP} = d_{NP}/\sigma_{NP}A_{NP}$ and $\sigma_{NP} = n_{NP}e\mu_{NP}$. Here, μ_{NP}, σ_{NP} and n_{NP} are the nanoparticle mobility, conductivity, and carrier density respectively.

It is important to note that we have discussed the equations above as describing the mobility of the network. However, strictly speaking they describe the mobility of carriers along a single current path (a linear array of nanoparticles). Because we are considering the network as an array of many parallel current paths, we assume the mobility of the network is the same as the mobility along an average current path.

The equations above show explicitly that $\mu_{Net}^{-1} \propto (R_J + R_{NP})$, which implies that the network resistivity also scales with the sum of junction and nanosheet resistances: $\rho_{Net} \propto (R_J + R_{NP})$. This is a fundamental feature of this model and reflects the fact that every time a charge carrier travels through a nanosheet it must then travel across a junction. It should be noted that this feature has been proposed previously[4, 5] and has independently been deduced empirically from experimental data[6].

1.2 Converting network mobility to network conductivity (or resistivity)

Method 1: Although this equation for network mobility will certainly be useful, it would be more useful to have an equation for network conductivity or resistivity. To achieve this, we need an expression for the carrier density of a network. In the simplest case, charge carriers exist only in the nanoparticles making up the network and not the pores in between. Then the network carrier density is given by $n_{Net} = n_{NP}(1-P_{Net})$, where P_{Net} is the network porosity.

It is worth noting that a more complicated scenario is possible. Studies on mixed networks of graphene and WS_2 nanosheets[7] have shown that the carrier density in the graphene network is given by $n_{Net} = n_{NP}(\phi-\phi_c)^a$, where ϕ is the graphene volume fraction, ϕ_c is the graphene percolation threshold and a is an exponent. Converting ϕ into porosity for a single-material nanoparticle network would imply: $n_{Net} = n_{NP}(P_c - P)^a$ where P_c is the porosity at the

percolation threshold. This expression is a generalised version of $n_{\text{Net}} = n_{\text{NP}}(1-P_{\text{Net}})$ but has the disadvantage that it has two extra variables. On balance we believe $n_{\text{Net}} = n_{\text{NP}}(1-P_{\text{Net}})$ is a reasonable approximation, especially in a single-material nanoparticle network (e.g. a network of silver nanowires or a network of MoS_2 nanosheets) as percolation effects such as the presence of dead ends are minimal.

Thus, in a network, we hypothesise that the carrier density scales as $n_{\text{Net}} = n_{\text{NP}}(1-P_{\text{Net}})$, where P_{Net} is the network porosity. This implies that $\sigma_{\text{Net}} = n_{\text{NP}} e \mu_{\text{Net}} (1-P_{\text{Net}})$, or

$$\sigma_{\text{Net}} \approx \frac{\sigma_{\text{NP}}(1-P_{\text{Net}})/\kappa_{\text{Net}}}{\left[1+\dfrac{R_{\text{J}}}{R_{\text{NP}}}\right]\left[1+\dfrac{d_{\text{NP}}/A_{\text{NP}}}{n_{\text{NP}} d_{\text{NP}}^{2}}\right]} \qquad \text{Equation S1}$$

We use the subscript "Net" because the tortuosity factor refers to the nanoparticle network. The $(1-P_{\text{Net}})/\kappa_{\text{Net}}$ part of this equation is consistent with models used for ion transport in electrolyte-filled pore networks[8] where the conductivity within a pore network is related to the conductivity in the bulk electrolyte by $\sigma_{\text{pore}} = \sigma_{\text{bulk}} P_{\text{Net}} / \kappa_{\text{pore}}$. Here, we are considering transport in the nanoparticle network so we use (1 - P_{Net}), the volume fraction of nanosheets instead of P_{Net}, the volume fraction of pores. We expect κ_{Net} to be a dimensionless number slightly larger than 1 and so can be approximated as 1 to first order[9].

It is important to note that Eqn. S1 is general. Up to this point we have not incorporated the nanoparticle geometry into the model. The geometric parameters, d_{NP} (the mean distance travelled by an electron within a nanoparticle) and A_{NP} (the effective cross-sectional area of the nanoparticle) are written in a geometry-independent way. This means Eqn. S1 could be applied to networks of 0D, 1D or 2D particles just by using appropriate values of d_{NP} and A_{NP}.

Method 2: We can derive the same equation more explicitly by considering that charge carriers flow along many parallel current paths, each one consisting of a linear chain of nanoparticle-junction pairs. Then the overall resistance of the network is $R_{\text{Net}} = R_{\text{Path}} / m$. Here R_{Path} is the resistance of a single current path and m is the number of current paths which we must consider in parallel. We can find m from the cross-sectional area of the film, A_{Net}, divided by the cross-sectional area occupied by each nanoparticle: $m = A_{\text{Net}} / (A_{\text{NP}} / (1-P_{\text{Net}}))$ where the factor $(1-P_{\text{Net}})$ accounts for the free space between nanosheets. This means $R_{\text{Net}} = R_{\text{Path}} A_{\text{NP}} / ((1-P_{\text{Net}}) A_{\text{Net}})$.

The resistance of a single current path is

$$R_{\text{Path}} = \frac{1}{\sigma_{\text{Path}}}\frac{L}{A_{\text{Path}}} = \frac{1}{n_{\text{NS}}e\mu_{\text{Path}}}\frac{L}{A_{\text{Path}}}$$

Where L is the channel length, σ_{Path} is the conductivity of the current path and A_{Path} is the cross-section area of the path (which is just a chain of nanoparticles). Combining equations for R_{Net} and R_{Path}

$$R_{\text{Net}} = \frac{1}{n_{\text{NP}}e\mu_{\text{Path}}}\frac{L}{A_{\text{Path}}}\frac{A_{\text{NP}}}{((1-P_{\text{Net}})A_{\text{Net}})}$$

Now, we note that because the current path is just a chain of nanoparticles, $A_{\text{Path}} = A_{\text{NP}}$. In addition, as discussed above, μ_{Path} and μ_{Net} are identical. This allows us to rearrange

$$\sigma_{\text{Net}} = n_{\text{NP}}e\mu_{\text{Net}}(1-P_{\text{Net}})$$

which is the same equation we used in Method 1.

We will now develop this model for the specific cases of 2D, 1D, and 0D nanoparticles.

1.3 2D Nanosheet Networks

While $R_{\text{NP}} = d_{\text{NP}}(\sigma_{\text{NP}}A_{\text{NP}})^{-1}$ is geometry-independent, we can make it specific to nanosheets (assuming they are square) by writing the cross-sectional area as $A_{\text{NS}} = l_{\text{NS}}t_{\text{NS}}$ where t_{NS} and l_{NS} are the mean nanosheet thickness and length. In addition, we need to relate d_{NS} (the mean distance travelled by an electron within a nanosheet) to the nanosheet length, l_{NS}. Depending on the stacking arrangement of nanosheets, d_{NS} could vary from close to zero (junctions where the electrons entered and left the nanosheet very close together) to close to the entire nanosheet length (junctions where the electrons entered and left the nanosheets very close to each end). Thus, on average, the distance travelled through the nanosheet is roughly half the nanosheet length such that $d_{\text{NS}} = l_{\text{NS}}/2$ (although this will not apply in cases such as Langmuir-Blodgett edge-to-edge connected monolayer networks[10]). This gives $d_{\text{NS}}/A_{\text{NS}} = (2t_{\text{NS}})^{-1}$. We can then obtain an equation for the conductivity of a nanosheet network as

$$\sigma_{\text{Net}} \approx \frac{\sigma_{\text{NS}}(1-P_{\text{Net}})/\kappa_{\text{Net}}}{\left[1+\dfrac{R_{\text{J}}}{R_{\text{NS}}}\right]\left[1+\dfrac{2}{n_{\text{NS}}t_{\text{NS}}l_{\text{NS}}^{2}}\right]} \qquad \text{Equation S2a}$$

Alternatively, this equation can be written in terms of network resistivity.

$$\rho_{\text{Net}} \approx \frac{\rho_{\text{NS}}\kappa_{\text{Net}}}{(1-P_{\text{Net}})}\left[1+\frac{R_{\text{J}}}{R_{\text{NS}}}\right]\left[1+\frac{2}{n_{\text{NS}}t_{\text{NS}}l_{\text{NS}}^{2}}\right] = \frac{2t_{\text{NS}}\kappa_{\text{Net}}}{(1-P_{\text{Net}})}\left[R_{\text{NS}}+R_{\text{J}}\right]\left[1+\frac{2}{n_{\text{NS}}t_{\text{NS}}l_{\text{NS}}^{2}}\right] \quad \text{Equation S2b}$$

Or

$$\rho_{\text{Net}} \approx \frac{\kappa_{\text{Net}}}{(1-P_{\text{Net}})}\left[\rho_{\text{NS}}+2t_{\text{NS}}R_{\text{J}}\right]\left[1+\frac{2}{n_{\text{NS}}t_{\text{NS}}l_{\text{NS}}^{2}}\right]$$

where we use $R_{\text{NS}} = d_{\text{NS}}(\sigma_{\text{NS}}A_{\text{NS}})^{-1} = (\sigma_{\text{NS}}2t_{\text{NS}})^{-1} = \rho_{\text{NS}}(2t_{\text{NS}})^{-1}$ and $\sigma_{\text{NS}} = en_{\text{NS}}\mu_{\text{NS}}$.

Or

$$\sigma_{\text{Net}} \approx \frac{n_{\text{NS}}e\mu_{\text{NS}}(1-P_{\text{Net}})/\kappa_{\text{Net}}}{\left[1+2R_{\text{J}}n_{\text{NS}}e\mu_{\text{NS}}t_{\text{NS}}\right]\left[1+\dfrac{2}{t_{\text{NS}}l_{\text{NS}}^{2}n_{\text{NS}}}\right]} \qquad \text{Equation S2c}$$

We note that the combination of parameters found in the denominator of the final term of this equation ($t_{\text{NS}}l_{\text{NS}}^{2}n_{\text{NS}}$) represents the number of free carriers in the nanosheet.

Eqns. S2a-c are general equations for conductivity in nanosheet networks. It is very important to note that we have taken a classical approach, treating the nanosheets as classical objects where $R_{\text{NS}} = d_{\text{NS}}(\sigma_{\text{NS}}A_{\text{NS}})^{-1}$. However, one could easily take a semi-quantum approach, for example by using $R_{\text{NS}} = R_{\text{Q}}d_{\text{NS}}/\zeta$ where R_{Q} is the resistance quantum and ζ is the scattering length.

Conduction Regimes

Looking at the second bracketed term in the denominator in Eqn. S2a, there are two main regimes that this model can reduce to depending on the carrier density. We elucidate this explicitly for 2D nanosheets but the same could be done for other geometries. For a network of 2D nanosheets, the resistivity is given by

$$\rho_{\text{Net}} \approx \frac{2t_{\text{NS}}\kappa_{\text{Net}}}{(1-P_{\text{Net}})}\left[R_{\text{NS}}+R_{\text{J}}\right]\left[1+\frac{2}{n_{\text{NS}}t_{\text{NS}}l_{\text{NS}}^{2}}\right]$$

For nanosheets with a low carrier density such as semiconductors, $n_{\mathrm{NS}} << 2(t_{\mathrm{NS}} l_{\mathrm{NS}}^2)^{-1}$. Then

$$\rho_{\mathrm{Net}} \approx \frac{4(R_{\mathrm{NS}} + R_{\mathrm{J}})\kappa_{\mathrm{Net}}}{(1-P_{\mathrm{Net}})n_{\mathrm{NS}} l_{\mathrm{NS}}^2} \qquad \text{or} \qquad \sigma_{\mathrm{Net}} \approx \frac{(1-P_{\mathrm{Net}})n_{\mathrm{NS}} l_{\mathrm{NS}}^2}{4(R_{\mathrm{NS}} + R_{\mathrm{J}})\kappa_{\mathrm{Net}}}$$

For this to be the case, the number of carriers per nanosheet $n_{\mathrm{NS}} t_{\mathrm{NS}} l_{\mathrm{NS}}^2 = n_{\mathrm{NS}} V_{\mathrm{NS}} = N_{\mathrm{NS}}$ must be very small (<< 2).

For nanosheets with a high carrier density such as highly doped semiconductors or conductors $n_{\mathrm{NS}} >> 2(t_{\mathrm{NS}} l_{\mathrm{NS}}^2)^{-1}$. Then

$$\rho_{\mathrm{Net}} \approx \frac{2t_{\mathrm{NS}}\kappa_{\mathrm{Net}}}{(1-P_{\mathrm{Net}})}\left[R_{\mathrm{NS}} + R_{\mathrm{J}}\right] \qquad \text{or} \qquad \sigma_{\mathrm{Net}} \approx \frac{(1-P_{\mathrm{Net}})}{2t_{\mathrm{NS}}(R_{\mathrm{NS}} + R_{\mathrm{J}})\kappa_{\mathrm{Net}}}$$

For this to be the case, the number of carriers per nanosheet $n_{\mathrm{NS}} t_{\mathrm{NS}} l_{\mathrm{NS}}^2 = n_{\mathrm{NS}} V_{\mathrm{NS}} = N_{\mathrm{NS}}$ must be large (>>2).

By now considering the relative magnitudes of R_{NS} and R_{J}, we can further develop these equations in five cases.

Case 1: A nanosheet network with a low carrier density and limited by inter-sheet junctions.

Then, $1 << 2(n_{\mathrm{NS}} t_{\mathrm{NS}} l_{\mathrm{NS}}^2)^{-1}$ and $R_{\mathrm{J}} / R_{\mathrm{NS}} >> 1$ which gives

$$\sigma_{\mathrm{Net}} \approx \frac{(1-P_{\mathrm{Net}}) l_{\mathrm{NS}}^2 n_{\mathrm{NS}}}{4R_{\mathrm{J}}\kappa_{\mathrm{Net}}}$$

e.g. the case of a junction-limited semiconductor. This equation is somewhat similar to one proposed in ref[5] and found via dimensional analysis. However, this equation contains l_{NS} whereas the previously proposed version wrongly included t_{NS}. The version presented here is correct.

Case 2: A nanosheet network with light doping and low junction resistance.

Then, $1 << 2(n_{\mathrm{NS}} t_{\mathrm{NS}} l_{\mathrm{NS}}^2)^{-1}$ and $R_{\mathrm{J}} / R_{\mathrm{NS}} << 1$ which gives

$$\sigma_{\mathrm{Net}} \approx \frac{(1-P_{\mathrm{Net}})n_{\mathrm{NS}} l_{\mathrm{NS}}^2}{4R_{\mathrm{NS}}\kappa_{\mathrm{Net}}} = \sigma_{\mathrm{NS}}(1-P_{\mathrm{Net}}) t_{\mathrm{NS}} l_{\mathrm{NS}}^2 n_{\mathrm{NS}} / 2\kappa_{\mathrm{Net}}$$

e.g. the case of a material-limited system with few carriers.

Case 3: A nanosheet network with a high carrier density and limited by inter-sheet junctions.

Then, $1 >> 2(n_{\mathrm{NS}} t_{\mathrm{NS}} l_{\mathrm{NS}}^2)^{-1}$ and $R_{\mathrm{J}} / R_{\mathrm{NS}} >> 1$ which gives

$$\sigma_{\mathrm{Net}} \approx \frac{(1-P_{\mathrm{Net}})}{2 t_{\mathrm{NS}} R_{\mathrm{J}} \kappa_{\mathrm{Net}}}$$

e.g. the case for junction-limited conductors like graphene or silver nanoplatelets, or a heavily doped semiconductor. This equation is very similar to one proposed in ref[5] and found via dimensional analysis. The version presented here is correct.

Case 4: A nanosheet network with a high carrier density and low junction resistance.

Then, $1 >> 2(n_{\mathrm{NS}} t_{\mathrm{NS}} l_{\mathrm{NS}}^2)^{-1}$ and $R_{\mathrm{J}} / R_{\mathrm{NS}} << 1$ which gives

$$\sigma_{\mathrm{Net}} \approx \sigma_{\mathrm{NS}} (1-P_{\mathrm{Net}}) / \kappa_{\mathrm{Net}}$$

e.g. the case for material limited system with many carriers (for example, heavily doped TMDs as published by Lin et al.[11]).

Special Case: Networks of nanosheets prepared by liquid-phase exfoliation (LPE).

A specific case which is particularly important here is that of networks of LPE nanosheets as used in Fig. 2 and Fig. 3 in the main text. Owing to the mechanics of the ultrasonication-based exfoliation process[12], LPE nanosheets tend to have a roughly constant aspect ratio, k_{NS}, (defined by nanosheet mechanics) allowing us to define a well-defined relationship between nanosheet length and thickness $k_{\mathrm{NS}} = l_{\mathrm{NS}} / t_{\mathrm{NS}}$. In this case, Eqn. S2a becomes

$$\sigma_{\mathrm{Net}} \approx \frac{n_{\mathrm{NS}} e \mu_{\mathrm{NS}} (1-P_{\mathrm{Net}})}{\left[1 + 2 R_{\mathrm{J}} n_{\mathrm{NS}} e \mu_{\mathrm{NS}} l_{\mathrm{NS}} / k_{\mathrm{NS}}\right]\left[1 + \frac{2 k_{\mathrm{NS}}}{l_{\mathrm{NS}}^3 n_{\mathrm{NS}}}\right]} \quad \text{Equation S3}$$

1.4 1D Nanotube/Nanowire Networks

As for the 2D case, Eqn. S1 can be modified to describe conduction in 1D networks. We take R_{NS} as the resistance (on average) of the portion of the nanowire the electron passes through between junctions (length, d_{NW}). Assuming each nanowire has many junctions with other nanowires, this distance could vary from close to zero (junctions where electrons entered and left the nanowire very close together) to close to the nanowire length, l_{NW}, (junctions where electrons entered and left the nanowire very close to each end). The average distance travelled

through the nanowire is roughly half the nanowire length allowing us to replace d_{NW} with $l_{NW}/2$. Then R_{NW} can be replaced by

$$R_{\mathrm{NW}} = (l_{\mathrm{NW}}/2)/(\sigma_{\mathrm{NW}}\pi D_{\mathrm{NW}}^2/4) = (2l_{\mathrm{NW}})/(\sigma_{\mathrm{NW}}\pi D_{\mathrm{NW}}^2)$$

where we take $A_{\mathrm{NS}} = \pi D_{\mathrm{NW}}^2/4$. Then

$$\sigma_{\mathrm{Net}} \approx \frac{\sigma_{\mathrm{NW}}(1-P_{\mathrm{Net}})/\kappa_{\mathrm{Net}}}{\left[1+\dfrac{R_{\mathrm{J}}\sigma_{\mathrm{NW}}\pi D_{\mathrm{NW}}^2}{2l_{\mathrm{NW}}}\right]\left[1+\dfrac{8}{n_{\mathrm{NW}}l_{\mathrm{NW}}\pi D_{\mathrm{NW}}^2}\right]}$$

or

$$\rho_{\mathrm{Net}} \approx \frac{\pi D_{\mathrm{NW}}^2\kappa_{\mathrm{Net}}}{2l_{\mathrm{NW}}(1-P_{\mathrm{Net}})}\left[R_{\mathrm{NW}}+R_{\mathrm{J}}\right]\left[1+\frac{8}{n_{\mathrm{NW}}l_{\mathrm{NW}}\pi D_{\mathrm{NW}}^2}\right]$$

Which simplifying further gives

$$\rho_{\mathrm{Net}} \approx \frac{\kappa_{\mathrm{Net}}}{(1-P_{\mathrm{Net}})}\left[\rho_{\mathrm{NW}}+\frac{\pi D_{\mathrm{NW}}^2 R_{\mathrm{J}}}{2l_{\mathrm{NW}}}\right]\left[1+\frac{8}{n_{\mathrm{NW}}l_{\mathrm{NW}}\pi D_{\mathrm{NW}}^2}\right]$$

If the 1D objects are conductors, n_{NW} is very large and meaning the above equation reduces to

$$\sigma_{\mathrm{Net}} \approx \frac{\sigma_{\mathrm{NW}}(1-P_{\mathrm{Net}})/\kappa_{\mathrm{Net}}}{\left[1+\dfrac{R_{\mathrm{J}}\sigma_{\mathrm{NW}}\pi D_{\mathrm{NW}}^2}{2l_{\mathrm{NW}}}\right]} \quad \text{or} \quad \rho_{Net} \approx \frac{\left[\rho_{\mathrm{NW}}+\dfrac{R_{\mathrm{J}}\pi D_{\mathrm{NW}}^2}{2l_{\mathrm{NW}}}\right]}{(1-P_{\mathrm{Net}})/\kappa_{\mathrm{Net}}} \qquad \text{Equation S4}$$

1.5 0D Nanoparticle Networks

We can also use Eqn. S1 to describe arrays of large, weakly coupled nanoparticles of diameter D_{NP}. Again, assuming bulk-like transport within the nanoparticle, we replace d_{NS} with D_{NP}, and R_{NS} can be replaced by $R_{\mathrm{NP}} \approx D_{\mathrm{NP}}/(\sigma_{\mathrm{NP}}D_{\mathrm{NP}}^2) = 1/(\sigma_{\mathrm{NP}}D_{\mathrm{NP}})$. We assume a tortuosity factor of $\kappa \approx 1$. Then

$$\sigma_{\mathrm{Net}} \approx \frac{\sigma_{NP}(1-P_{\mathrm{Net}})}{\left[1+\sigma_{\mathrm{NP}}D_{\mathrm{NP}}R_{\mathrm{J}}\right]\left[1+\dfrac{1}{n_{\mathrm{NP}}D_{\mathrm{NP}}^3}\right]}$$

If the 0D nanoparticles are conducting, n_{NP} is very large and

$$\sigma_{\text{Net}} \approx \frac{\sigma_{NP}(1-P_{\text{Net}})}{\left[1+\sigma_{\text{NP}} D_{\text{NP}} R_{\text{J}}\right]}$$

Which can alternatively be written as

$$\rho_{\text{Net}} \approx \frac{D_{\text{NP}}\left[R_{\text{NS}}+R_{\text{J}}\right]\left[1+\frac{1}{n_{\text{NP}} D_{\text{NP}}^{3}}\right]}{(1-P_{\text{Net}})}$$

1.6 Visualising the model

In general, for all dimensionalities, the model predicts Eqn. S1.

$$\sigma_{\text{Net}} \approx \frac{\sigma_{\text{NP}}(1-P_{\text{Net}})/\kappa_{\text{Net}}}{\left[1+\frac{R_{\text{J}}}{R_{\text{NP}}}\right]\left[1+\frac{d_{\text{NP}}/A_{\text{NP}}}{n_{\text{NP}} d_{\text{NP}}^{2}}\right]}$$

This can be written in terms of network resistivity as

$$\rho_{\text{Net}} \approx \rho_{\text{NS}} \frac{\kappa_{\text{Net}}}{(1-P_{\text{Net}})}\left[1+\frac{R_{\text{J}}}{R_{\text{NP}}}\right]\left[1+\frac{d_{\text{NP}}/A_{\text{NP}}}{n_{\text{NP}} d_{\text{NP}}^{2}}\right]$$

Clearly this is a multi-parameter equation that takes different forms in different dimensionalities. However, it can written in a useful simplified form which applies in 1D and 2D cases by noting that in those dimensionalities (as described above): $d_{\text{NP}} = l_{\text{NP}}/2$. Combining this with the fact that $l_{\text{NP}} A_{\text{NP}} n_{\text{NP}} = N_{\text{NP}}$, the number of charge carriers per nanoparticle and approximating $\kappa_{\text{Net}} = (1-P_{\text{Net}}) = 1$ yields

$$\frac{\rho_{\text{Net}}}{\rho_{\text{NP}}} \approx \left[1+\frac{R_{\text{J}}}{R_{\text{NP}}}\right]\left[1+\frac{2}{N_{\text{NP}}}\right]$$

Which is valid for 1D and 2D particles. We believe this is the simplest possible form in which our model can be expressed. It is useful because it allows visualisation as shown in Suppl. Fig. 2.

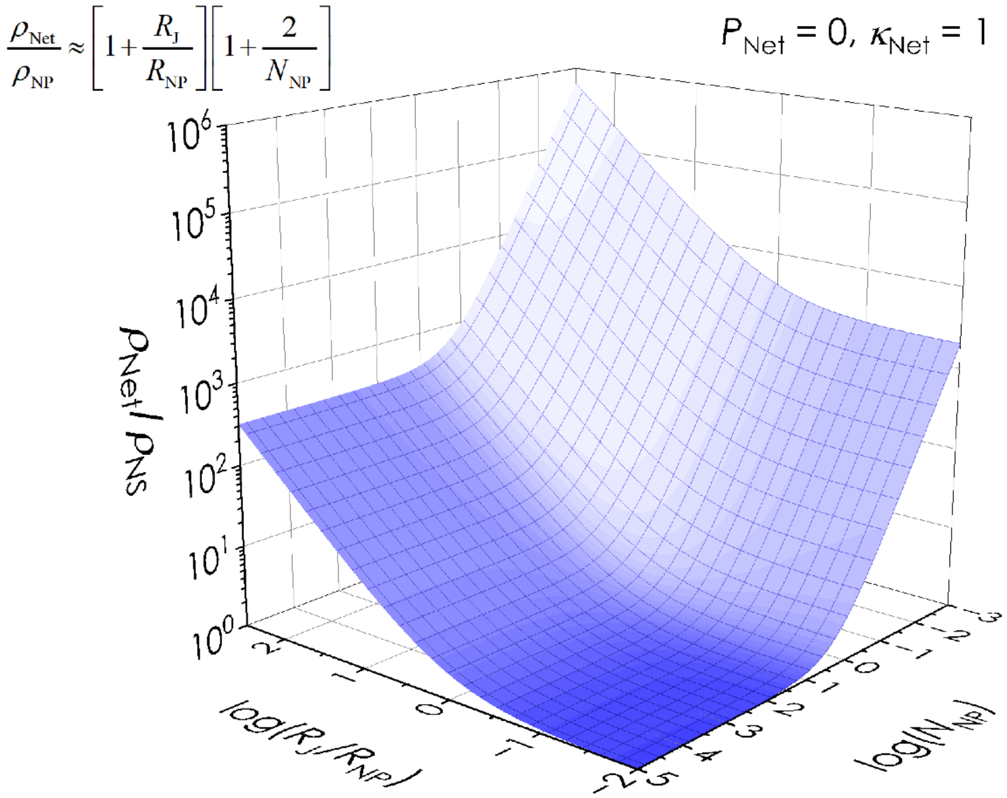

Supplementary figure 2: Visualisation of the network resistivity model. Plot of network resistivity, ρ_{Net}, (normalised to nanoparticle resistivity, ρ_{NP}) versus R_J/R_{NP} and N_{NP} (both on log scales).

This graph clearly shows the rich behaviour predicted by our model (and validated in the main text). In addition, it clearly illustrates the regimes discussed above.

However, the parameters on the axes of Suppl. Fig. 2 are somewhat unusual and may be unfamiliar to some readers. It is possible to reduce the number of variables in our network resistivity equation, specifically in the case of 2D materials by noting the following. In the main text (Fig. 3f) there is a clear relationship between junction resistance, R_J, and nanosheet resistivity, ρ_{NS}, as reproduced in Suppl. Fig. 3a. The red line shows an approximately linear relationship as defined by $R_J = \rho_{NS}/b$, where b = 1 nm. Combining this with the constant aspect ratio approximation ($k_{NS} = l_{NS}/t_{NS}$) applicable to liquid phase exfoliated nanosheets, gives a simplified equation for network resistivity as shown in the inset of Suppl. Fig. 3b. This allows us to plot network resistivity (normalised to nanosheet resistivity) versus l_{NS} and n_{NS} (Suppl. Fig. 3b). This clearly shows that, for LPE nanosheets with a carrier density below ~10^{23} m^{-3}, there exists a specific value of l_{NS} which results in the minimisation of network resistivity and so maximisation of network mobility. This optimal nanosheet length clearly depends on carrier density and so, for semiconducting nanosheets, doping level. However, for

completeness, we must also consider another scenario. For nanosheets produced by electrochemical exfoliation, the constant aspect ratio approximation does not hold. Probably more appropriate is to make the approximation that the nanosheet thickness is roughly constant (t_0). This yields a simplified equation for network resistivity as shown in the inset of Suppl. Fig. 3c. This allows us to plot network resistivity (normalised to nanosheet resistivity) versus l_{NS} and n_{NS} in a manner appropriate to EE nanosheets (Suppl. Fig. 3c). This plot shows no optimal nanosheet size.

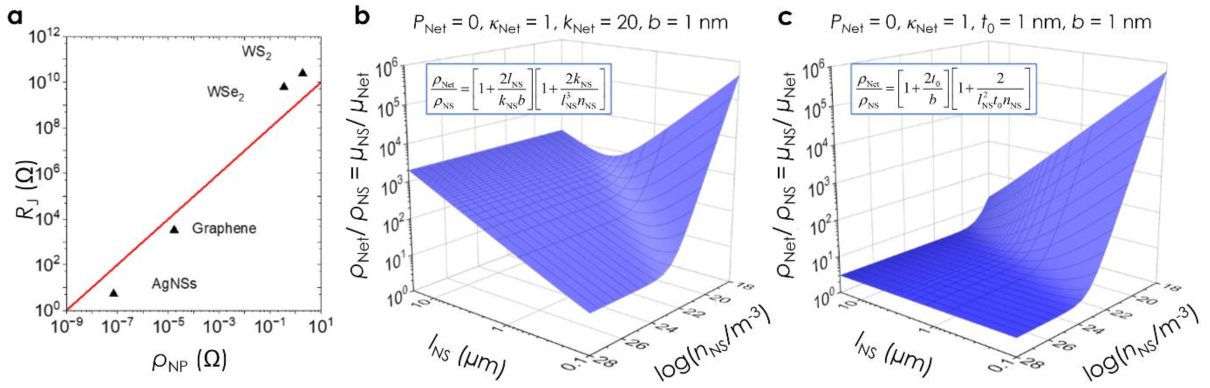

Supplementary figure 3: Visualisation of the network resistivity model for LPE and EE nanosheets. (a) Scaling between junction resistance, R_J, and nanosheet resistivity, ρ_{NS}, reproduced from the main text (Fig. 3f). The red line shows an approximately linear relationship as defined by $R_J = \rho_{NS} / b$, where b = 1 nm. **(b-c)** Plots of network resistivity (normalised to nanosheet resistivity) versus l_{NS} and n_{NS} for **(b)** liquid phase exfoliated (LPE) nanosheets (defined by $k_{NS} = l_{NS} / t_{NS}$) and **(c)** electrochemically exfoliated (EE) nanosheets (defined by constant thickness, t_0). In both cases, they are plotted using the approximation $R_J = \rho_{NS} / b$ (b = 1 nm).

1.7 Comparison with a previous model

Recently, Forro et al. published a model for conduction in nanowire networks which used a mean field approach to analyse a complex network of wires consisting of many parallel current paths[13]. This model resulted in an equation for the network sheet resistance, R_s, given by

$$R_s = \frac{R_{NW} / D_{Net}}{\left[\frac{1}{2} r_m - \sqrt{\frac{R_J r_m}{2 n_a R_{NW}}} \tanh\left(\sqrt{\frac{n_a R_{NW} r_m}{2 R_J}}\right)\right]} \qquad \text{Equation S5}$$

Where we have modified their notation to use R_{NW} and R_J. In addition, they use the following parameters:

$D_{\text{Net}} = \frac{N_{\text{NW}}}{A} l_{\text{NW}}^2$ is a measure of nanowire network coverage.

(N_{NW}/A) is the number of nanowires per unit area).

n_{a} is a measure of the number of junctions per nanowire.

The parameter r_{m} is a measure of the portion of the nanowire length through which current flows and is given by

$$r_{\text{m}} = \frac{1}{n_{\text{a}}+1}\left[n_{\text{a}} - 1 + \frac{R_{\text{J}}}{R_{\text{J}} + R_{\text{NW}}/(n_{\text{a}}+1)}\right] = \frac{1}{n_{\text{a}}+1}\left[n_{\text{a}} - 1 + \frac{R_{\text{J}}/R_{\text{NW}}}{R_{\text{J}}/R_{\text{NW}} + 1/(n_{\text{a}}+1)}\right]$$

Here, we aim to show that this model is similar in form to our equation for the conductivity of metallic nanowire networks (Eqn. S4). This will require a number of reasonable approximations.

First, we define an effective thickness of the network, t, which can be calculated considering the mass of nanowires per unit area, M/A

$$\frac{M}{A} = \rho_{\text{M,Net}} t = \frac{N_{\text{NW}}}{A} \rho_{\text{M,NW}} A_{\text{NW}} l_{\text{NW}}$$

where A_{NW} is the nanowire cross-sectional area. Here, $\rho_{\text{M,Net}}$ and $\rho_{\text{M,NW}}$ are the network and nanowire mass densities. Combining this equation with the definition of D_{Net} to eliminate N_{NW}/A yields

$$D_{\text{Net}} = \frac{\rho_{\text{M,Net}}}{\rho_{\text{M,NW}}} \frac{l_{\text{NW}}}{A_{\text{NW}}} t = (1 - P_{\text{Net}}) \frac{l_{\text{NW}}}{A_{\text{NW}}} t$$

Then, using the definition of sheet resistance and expressing t in terms of D_{Net}

$$\rho_{\text{Net}} = R_{\text{s}} t = \frac{R_{\text{s}} D_{\text{Net}}}{(1 - P_{\text{Net}})} \frac{A_{\text{NW}}}{l_{\text{NW}}}$$

Combining these equations with Eqn. S5 yields

$$\rho_{\text{Net}} = \frac{1}{(1 - P_{\text{Net}})} \frac{\rho_{\text{NW}}}{\left[\frac{1}{2} r_{\text{m}} - \sqrt{\frac{R_{\text{J}} r_{\text{m}}}{2 n_{\text{a}} R_{\text{NW}}}} \tanh\left(\sqrt{\frac{n_{\text{a}} R_{\text{NW}} r_{\text{m}}}{2 R_{\text{J}}}}\right)\right]}$$

Which can be rearranged to give

$$\rho_{\text{Net}} = \frac{1}{(1-P_{\text{Net}})} \frac{2\rho_{\text{NW}} / r_{\text{m}}}{\left[1 - \sqrt{\frac{2R_J}{n_{\text{a}} R_{\text{NW}} r_{\text{m}}}} \tanh\left(\sqrt{\frac{n_{\text{a}} R_{\text{NW}} r_{\text{m}}}{2R_{\text{J}}}}\right)\right]}$$

Finally, applying $R_{\text{NW}} = 4\rho_{\text{NW}} l_{\text{NW}} / (\pi D_{\text{NW}}^2)$

$$\rho_{\text{Net}} = \frac{1}{(1-P_{\text{Net}})} \frac{2\rho_{\text{NW}} / r_{\text{m}}}{\left[1 - \sqrt{\frac{R_{\text{J}} \pi D_{\text{NW}}^2}{2n_{\text{a}} \rho_{\text{NW}} l_{\text{NW}} r_{\text{m}}}} \tanh\left(\sqrt{\frac{2n_{\text{a}} \rho_{\text{NW}} l_{\text{NW}} r_{\text{m}}}{R_{\text{J}} \pi D_{\text{NW}}^2}}\right)\right]} \qquad \text{Equation S6}$$

We note that this equation is simply Forro's equation (Eqn. S5) rewritten and expressed in terms of network resistivity via a definition of effective network thickness and expressed via intensive properties. We can compare this with Eqn. S4 which we reproduce here for convenience:

$$\rho_{\text{Net}} \approx \frac{\left[\rho_{\text{NW}} + \frac{R_{\text{J}} \pi D_{\text{NW}}^2}{2l_{\text{NW}}}\right]}{(1-P_{\text{Net}}) / \kappa_{\text{Net}}} \qquad \text{Equation S4}$$

Limit of large R_{NW} and l_{NW}

To enable the comparison, we first approximate Eqn. S6 in the limit where $\left(\frac{2n_{\text{a}} \rho_{\text{NW}} l_{\text{NW}} r_{\text{m}}}{R_{\text{J}} \pi D_{\text{NW}}^2}\right)$ (i.e. $n_{\text{a}} R_{\text{NW}} r_{\text{m}} / (2R_{\text{J}})$) is large. This is true when $R_{\text{NW}} >> R_{\text{J}}$, e.g. when l_{NW} is large. Then, we find

$$\rho_{\text{Net}} \approx \frac{2\rho_{\text{NW}} / r_{\text{m}}}{(1-P_{\text{Net}})}$$

because when x is large $\tanh x \to 1$ and so $x^{-1} \tanh x \to 0$. We can compare Eqn. S4 in the limit of large R_{NW} and l_{NW}

$$\rho_{\text{Net}} \approx \frac{\rho_{\text{NW}} \kappa_{\text{Net}}}{(1-P_{\text{Net}})}$$

Clearly these limiting cases are similar. However, it is possible to extend the comparison.

By using the equation for r_{m} to generate a 3D graph of r_{m} versus n_{a} (range 0 - 100) and $R_{\text{J}}/R_{\text{NW}}$ (range 10^{-3} - 10^3) it can be shown that r_{m} is generally close to 1, lying between 0.5 and 1 in almost all of this range. This allows us to approximate $r_{\text{m}} \approx 1$.

In addition, one can roughly estimate the nanowire network's tortuosity factor, κ_{Net}. This parameter is defined as the square of the ratio of path length ($\propto a$ in Suppl. Fig. 4) to straight-line distance travelled by the charge carrier ($\propto b$ in Suppl. Fig. 4). If we imagine a very simple model where the carrier travels along a zig-zag path through a set of nanowires all at 45° to the overall direction of travel (Suppl. Fig. 4), then clearly, within this model $\kappa_{\text{Net}} = (a/b)^2 = 2$. This is probably a reasonable approximation for real nanowire networks, especially those with in-plane alignment.

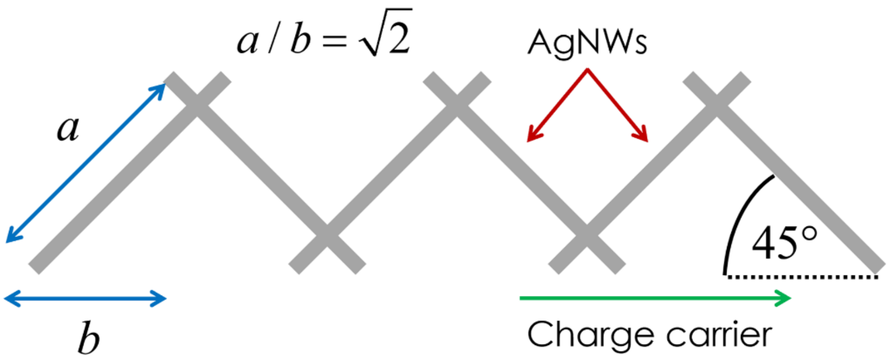

Supplementary figure 4: Estimation of the AgNW network tortuosity factor. Schematic of an AgNW network where the nanowires are arranged at 45° to the overall direction of travel (green arrow). The distance travelled by a charge along a given AgNW is given by *a*, while the straight-line distance travelled by the same charge, in the overall direction of travel, is given by *b*.

Taken together, these arguments mean the large-l_{NW} approximations to Eqn. S4 and Eqn. S6 are identical with both yielding $\rho_{\text{Net}} \approx 2\rho_{\text{NW}}/(1-P_{\text{Net}})$.

Limit of small R_{NW} and l_{NW}

Now we examine Eqn. S6 in the limit where $n_{\text{a}} R_{\text{NW}} r_{\text{m}} / (2R_{\text{J}})$ is small, an approximation that is reasonable so long as $R_{\text{NW}} < 2R_{\text{J}} / (n_{\text{a}} r_{\text{m}})$ i.e. when l_{NW} is small. To do this, we expand the hyperbolic tangent to second order ($\tanh x \approx x - x^3/3$), to obtain a much-simplified equation

$$\rho_{\text{Net}} \approx \frac{3R_{\text{J}}\pi D_{\text{NW}}^2}{(1-P_{\text{Net}})n_{\text{a}} r_{\text{m}}^2 l_{\text{NW}}}$$

In the same limit, Eqn. S4 is given by

$$\rho_{\mathrm{Net}} \approx \frac{R_{\mathrm{J}} \pi D_{\mathrm{NW}}^2 \kappa_{\mathrm{Net}}}{(1-P_{\mathrm{Net}}) 2 l_{\mathrm{NW}}}$$

These equations are very similar in form and are identical so long as $n_{\mathrm{a}} = 6/(r_{\mathrm{m}}^2 \kappa_{\mathrm{Net}})$.

As above, we can approximate $r_{\mathrm{m}} \approx 1$ and $\kappa_{\mathrm{Net}} \approx 2$ (Suppl. Fig. 4), which implies $n_{\mathrm{a}} \approx 3$. We interpret this as an effective number of junctions per nanowire, defining the number of junctions which are active on average for any given nanowire.

If we assume there are three active junctions per nanowire, then on average they are spaced $l_{\mathrm{NW}}/3$ apart. This means the average distance travelled through the nanowire by carriers entering and leaving by all combinations of these three junctions is $(l_{\mathrm{NW}}/3 + l_{\mathrm{NW}}/3 + 2l_{\mathrm{NW}}/3)/3 = 4l_{\mathrm{NW}}/9$. This is very close to the approximation made in the derivation of Eqn. S4 that the average distance travelled through each nanowire is $l_{\mathrm{NW}}/2$. This means Eqn. S4 and Eqn. S6 are consistent with each other in the limit of small R_{NW} and l_{NW}.

Given the fact that Eqn. S4 and Eqn. S6 are almost identical in both limits of large and small R_{NW} and l_{NW}, we can argue that they are very similar and largely consistent in almost all circumstances. This means that even though the approaches taken in this paper and that of Forro et al. were very different, they are self-consistent in terms of the equations they produce.

We can confirm this similarity by plotting Forro's equation (Eqn. S6) and our equation for nanowire network resistivity (Eqn. S4) on the same graph using the same parameters (see Suppl. Fig. 5 for values used). This confirms graphically that Forro's model and our own model are self-consistent (Suppl. Fig. 5).

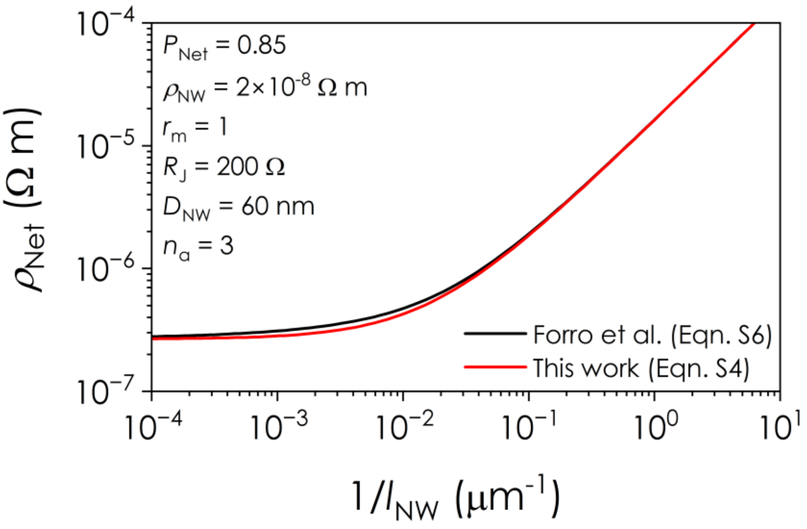

Supplementary figure 5: Comparison of predicted network resistivity for AgNWs using different models. Plots of AgNW network resistivity, ρ_{Net}, versus inverse nanowire length, $1/l_{\mathrm{NW}}$, using equations developed by Forro et al[13] (Eqn. S6) and in this work (Eqn. S4) on the same graph using the same parameters. The values used to plot the data are given in the panel.

Supplementary note 2. Nanosheet and nanowire size analysis

2.1. Silver Nanowires (AgNWs)

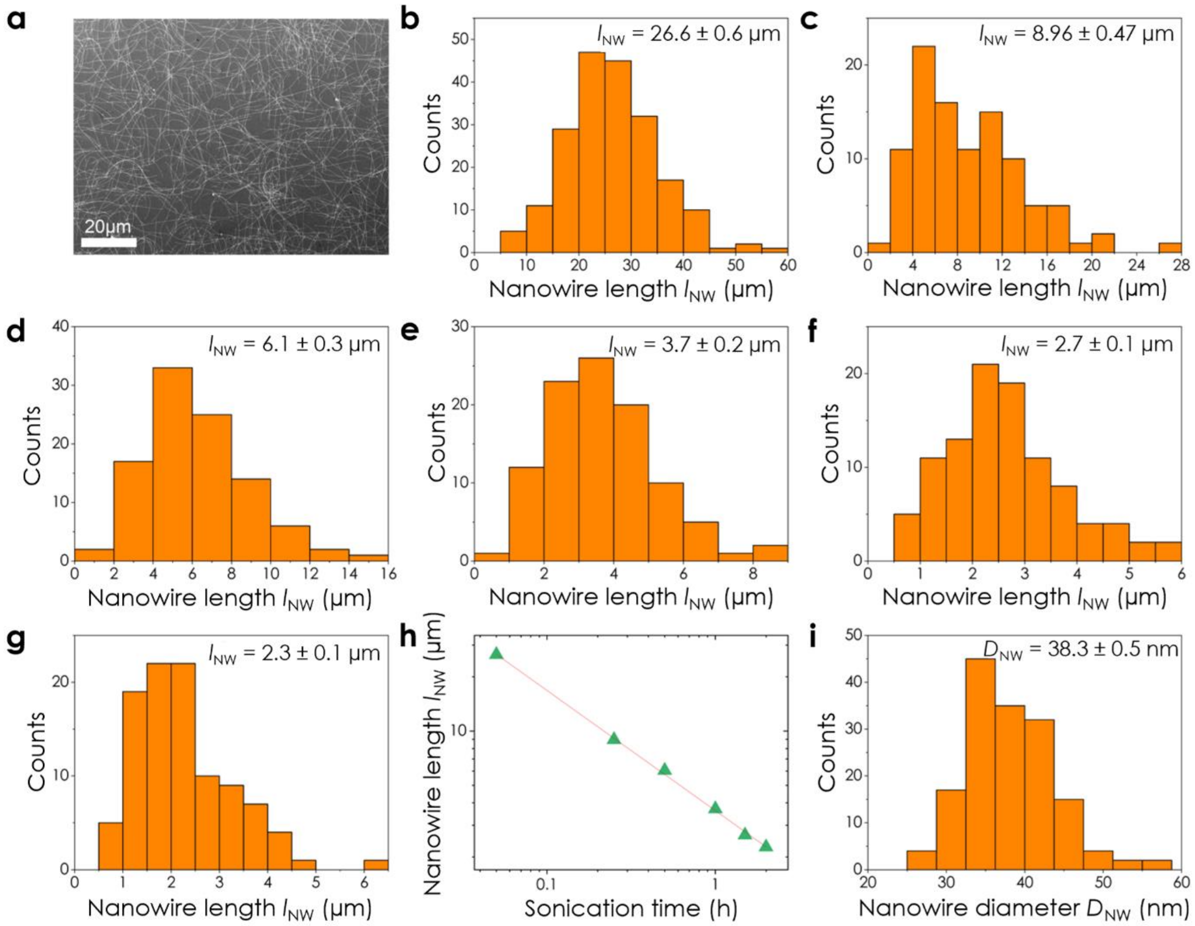

Supplementary figure 6: AgNW size analysis. (a) Representative SEM image of silver nanowires (AgNWs) with a nanowire length, $l_{NW,}$ of 26.6 ± 0.6 μm. **(b-g)** SEM histograms of l_{NW} for increasing sonication times of **(b)** 0.05, **(c)** 0.25, **(d)** 0.5, **(e)** 1, **(f)** 1.5 and **(g)** 2 hours. **(h)** Mean nanowire length as a function of sonication time. **(i)** Histogram of nanowire diameters, D_{NW}, measured using SEM.

2.2. Silver Nanosheets (AgNS)

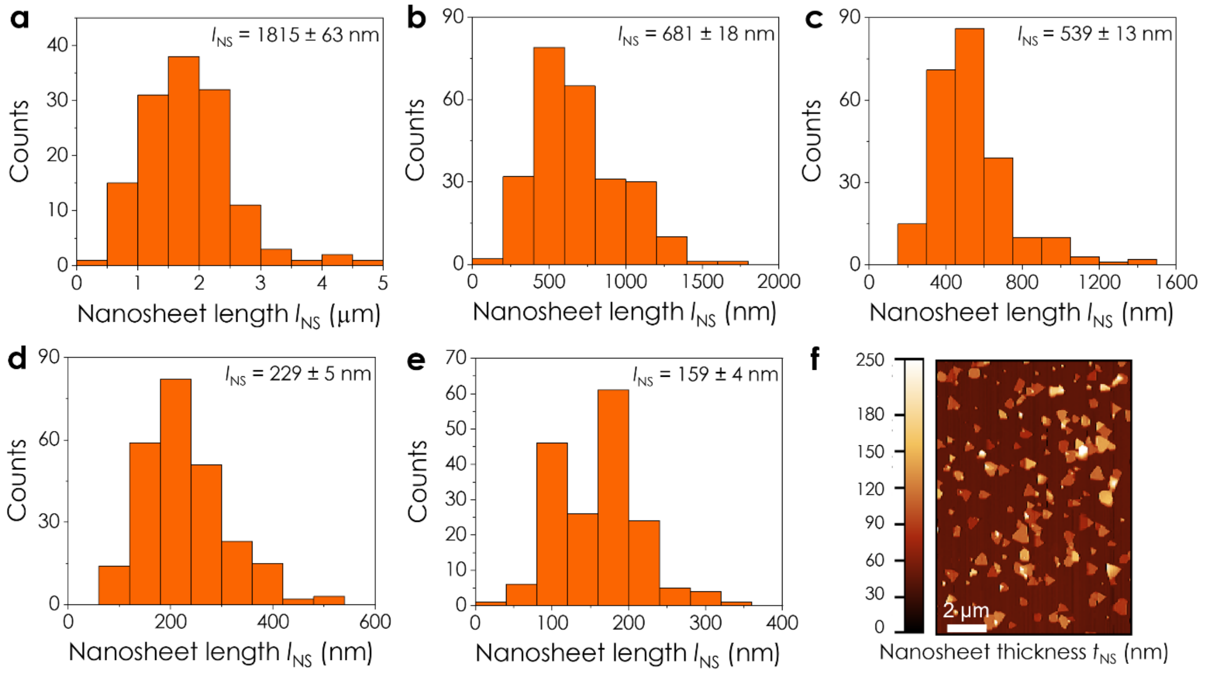

Supplementary figure 7: AFM histograms for silver nanosheet (AgNS) length. (a-e) Distributions of nanosheet length, l_{NS}, measured using AFM for the size-selected AgNS inks. The distributions are sorted by decreasing l_{NS}. **(f)** Representative AFM image for AgNSs with l_{NS} = 681 nm.

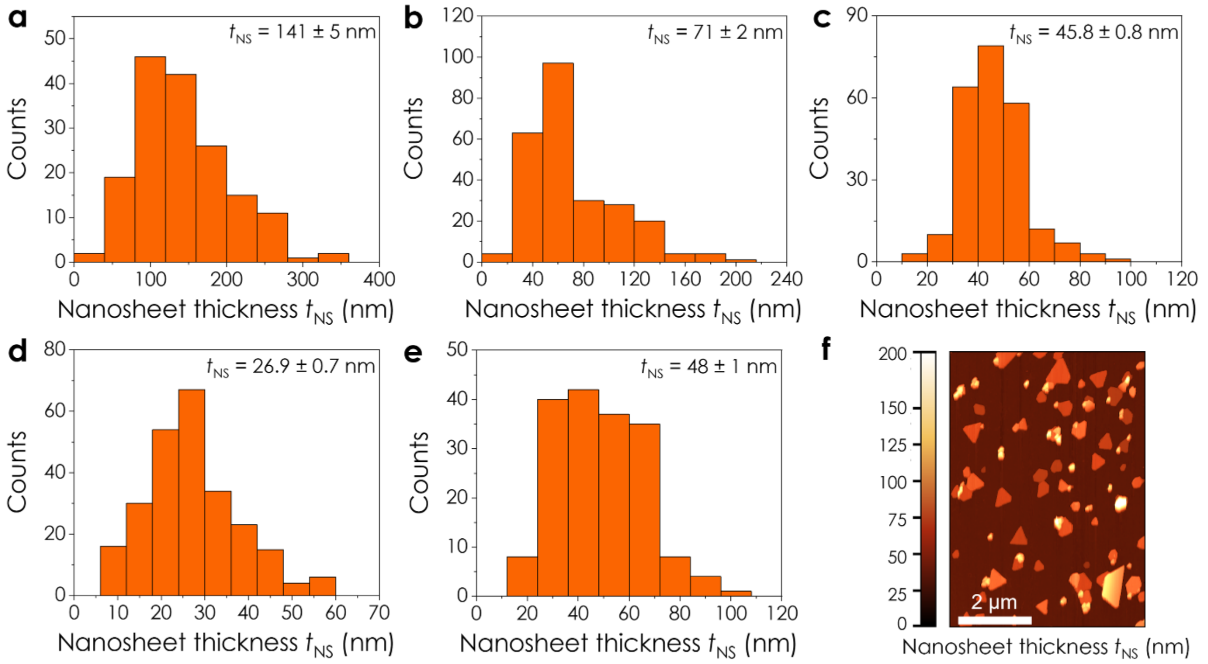

Supplementary figure 8: AFM histograms for silver nanosheet (AgNS) thickness. (a-e) Distributions of nanosheet thickness, t_{NS}, measured using AFM for the size-selected AgNS inks. The distributions are sorted by increasing centrifugation speed. **(f)** Representative AFM image for AgNSs with a nanosheet thickness t_{NS} = 43.8 nm.

2.3. Graphene

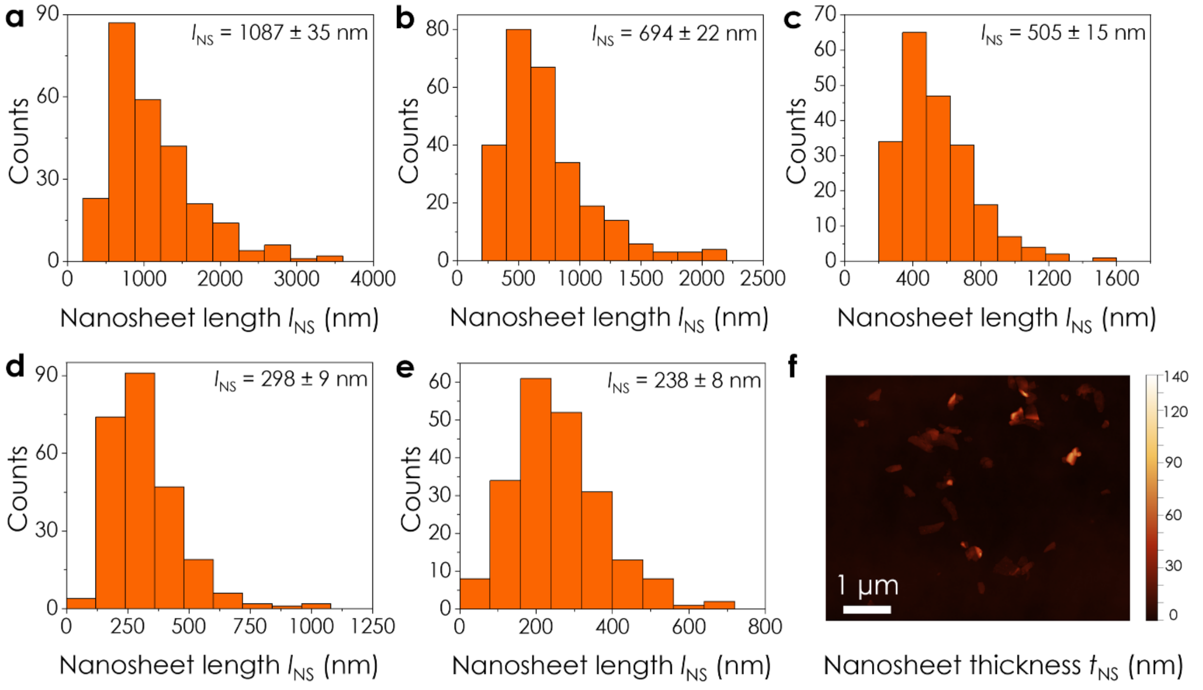

Supplementary figure 9: AFM histograms for graphene nanosheet length. (a-e) Distributions of nanosheet length, l_{NS}, measured using AFM for the size-selected graphene inks. The distributions are sorted by decreasing l_{NS}. **(f)** Representative AFM image for a graphene ink with l_{NS} = 298 nm.

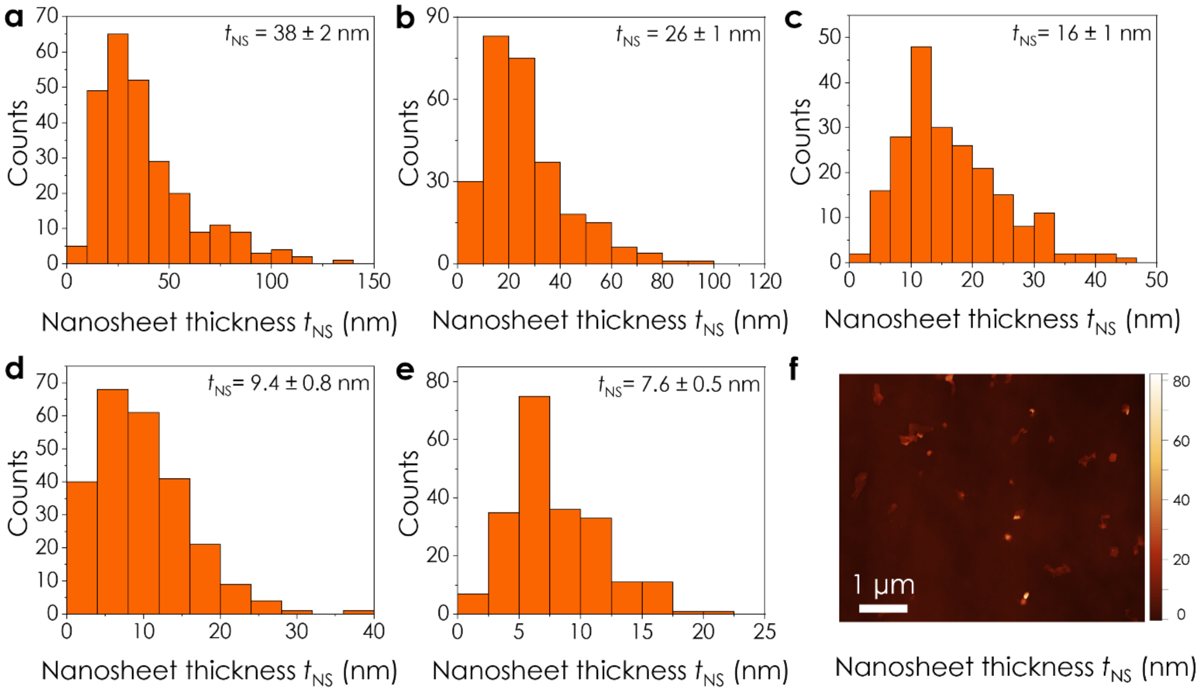

Supplementary figure 10: AFM histograms for graphene nanosheet thickness. (a-e) Distributions of nanosheet thickness, t_{NS}, measured using AFM for the size-selected graphene inks. The distributions are sorted by decreasing t_{NS}. **(f)** Representative AFM image for a graphene ink with t_{NS} = 7.6 nm.

2.4. WS_2

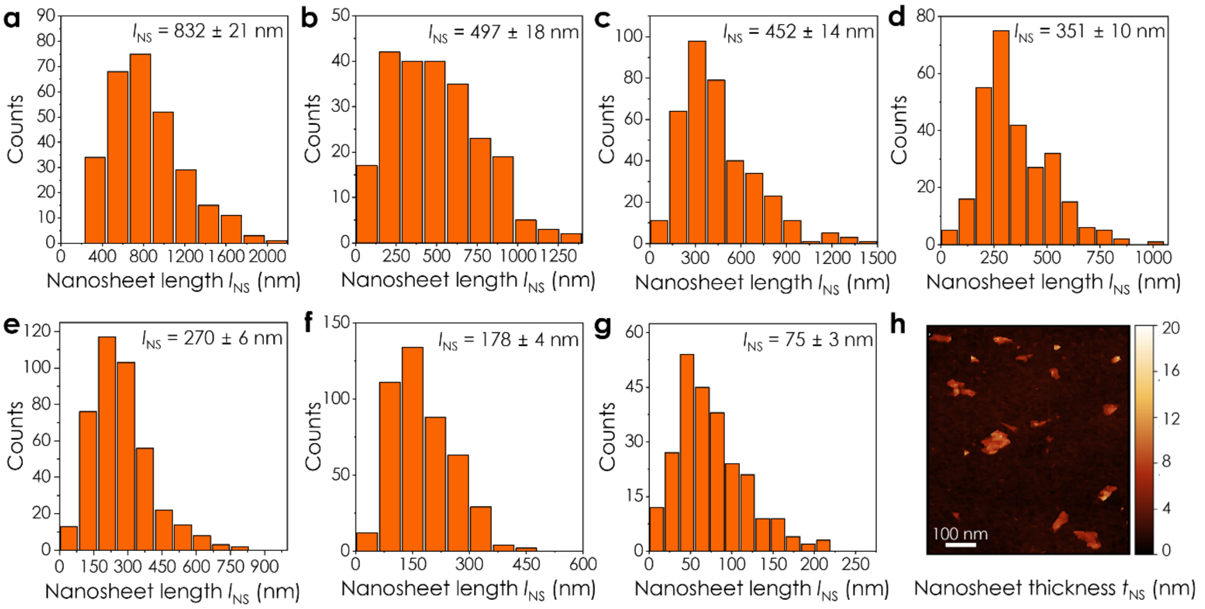

Supplementary figure 11: AFM histograms for WS_2 nanosheet length. (a-g) Distributions of nanosheet length, l_{NS}, measured using AFM for the size-selected WS_2 inks. The distributions are sorted by decreasing l_{NS}. **(h)** Representative AFM image for a WS_2 ink with l_{NS} = 75 nm.

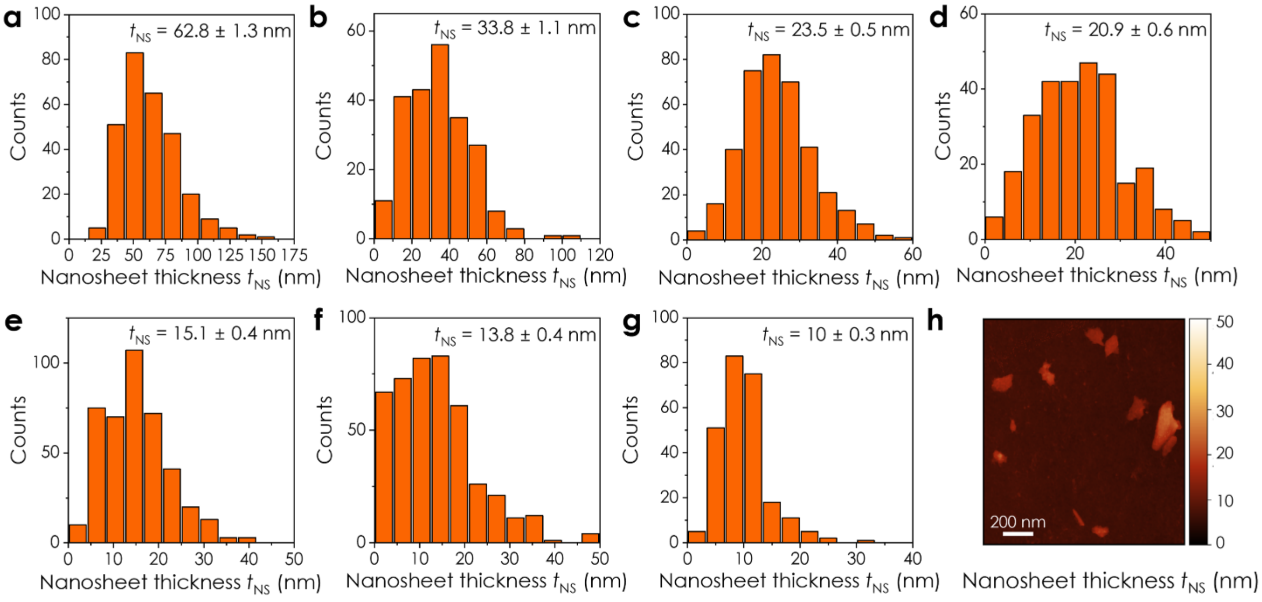

Supplementary figure 12: AFM histograms for WS_2 nanosheet thickness. (a-g) Distributions of nanosheet thickness, t_{NS}, measured using AFM for the size-selected WS_2 inks. The distributions are sorted by decreasing t_{NS}. **(h)** Representative AFM image for a WS_2 ink with t_{NS} = 23 nm.

2.5. WSe_2

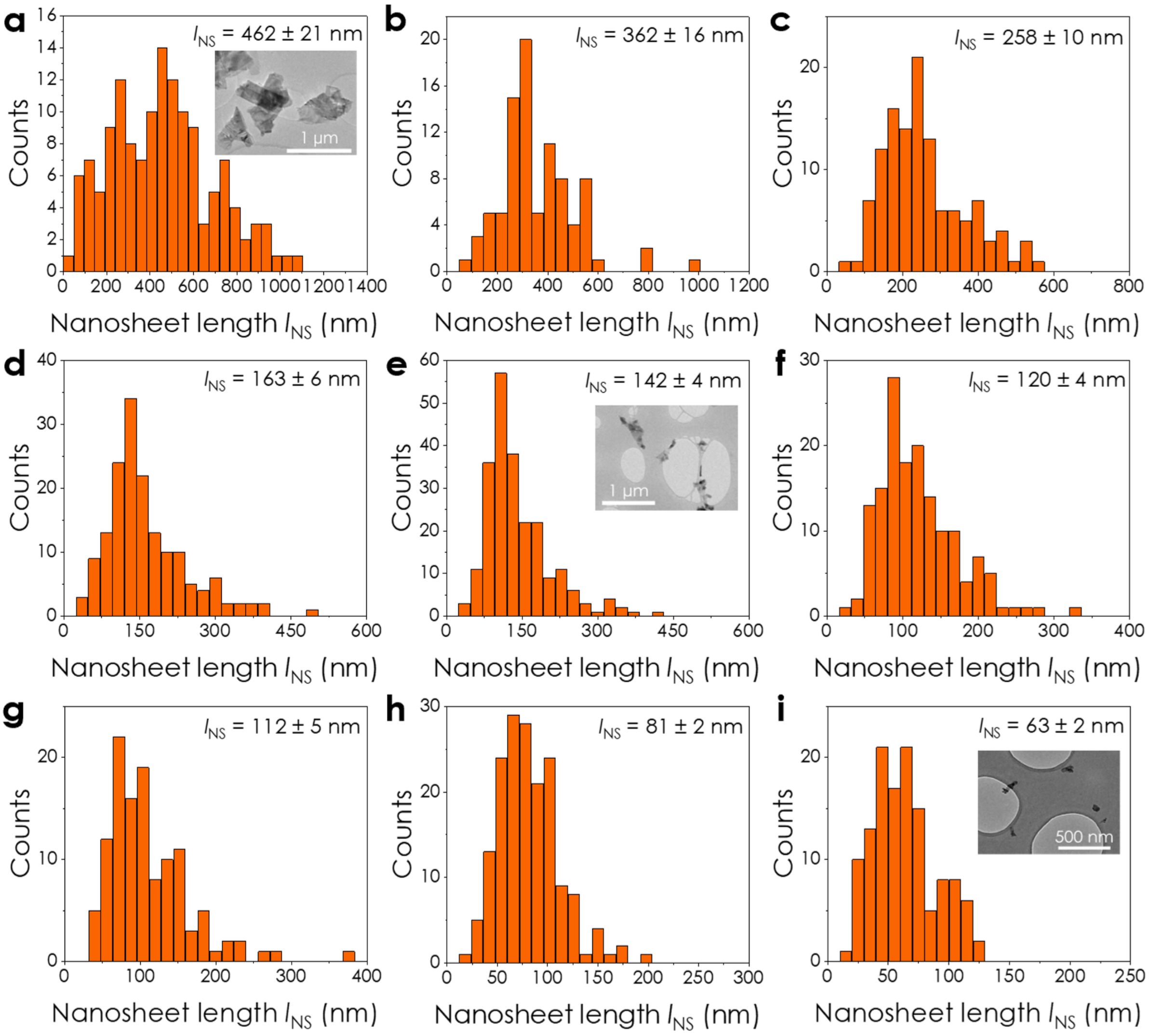

Supplementary figure 13: TEM histograms for WSe_2 nanosheet length. (a-i) Distributions of nanosheet length, l_{NS}, measured using TEM for the size-selected WSe_2 inks. The distributions are sorted by decreasing nanosheet length. The insets in **(a)**, **(e)** and **(i)** show representative TEM images.

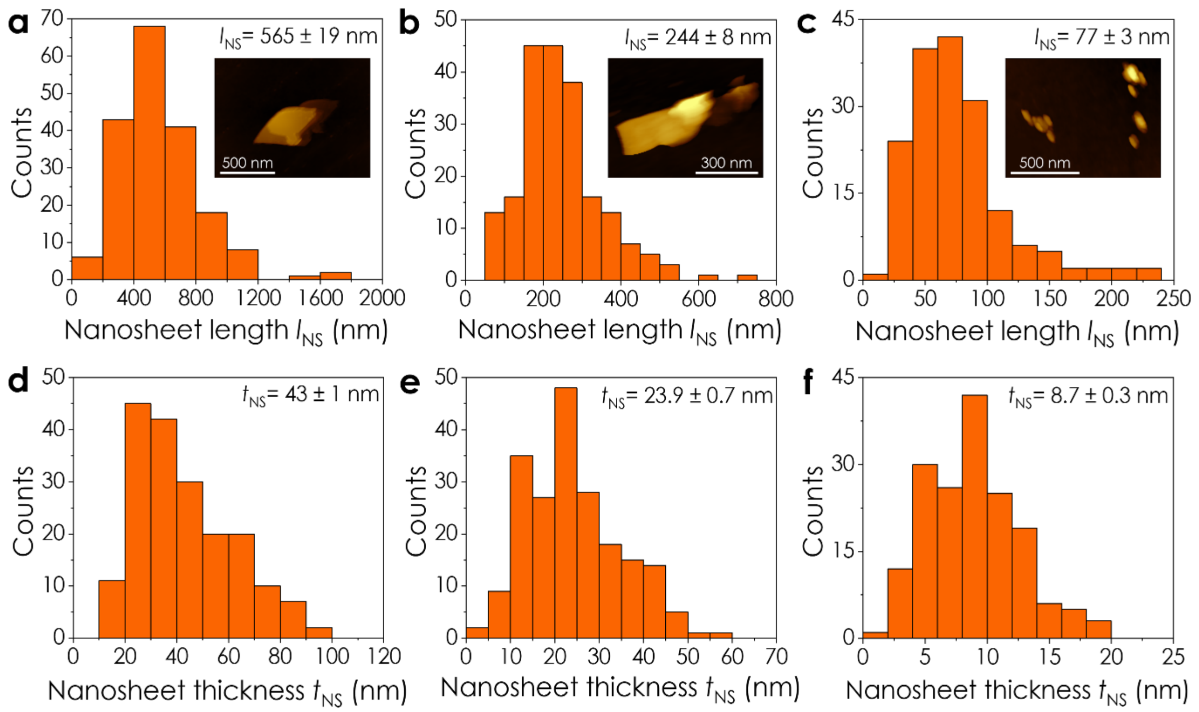

Supplementary figure 14: Distributions of WSe_2 nanosheet length, l_{NS}, and thickness, t_{NS}, measured by AFM. (a-c) Length distributions for the dispersions captured at 112× g, 699× g and 11180× g respectively. Inset: Representative AFM images for nanosheets from each size-selected fraction**. (d-f)** Thickness distributions for the dispersions captured at 112× g, 699× g and 11180× g respectively.

2.6 Comparison of microscopy and spectroscopy

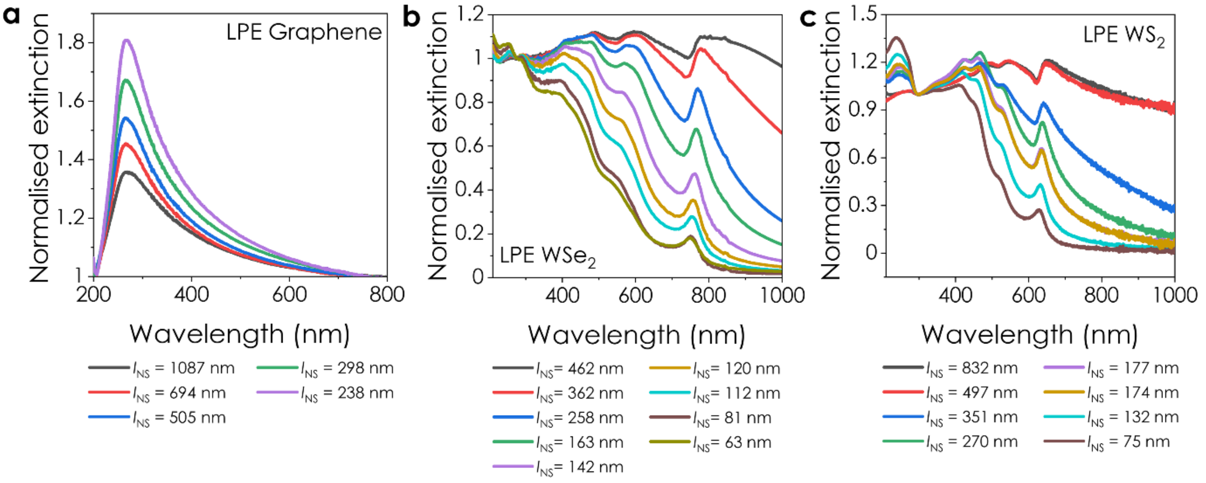

Supplementary figure 15: UV-vis extinction spectroscopy of size-selected liquid exfoliated nanosheets. Such spectra are known to be quite sensitive to nanosheet size[14, 15]. **(a)** Optical extinction spectra for the size-selected graphene inks normalised to the dimension-independent plateau at 750 nm. **(b)** Optical extinction spectra for the size-selected WSe_2 inks normalised to local minimum at 276 nm. **(c)** Optical extinction spectra for the size-selected WS_2 inks normalised to the local minimum at 294 nm.

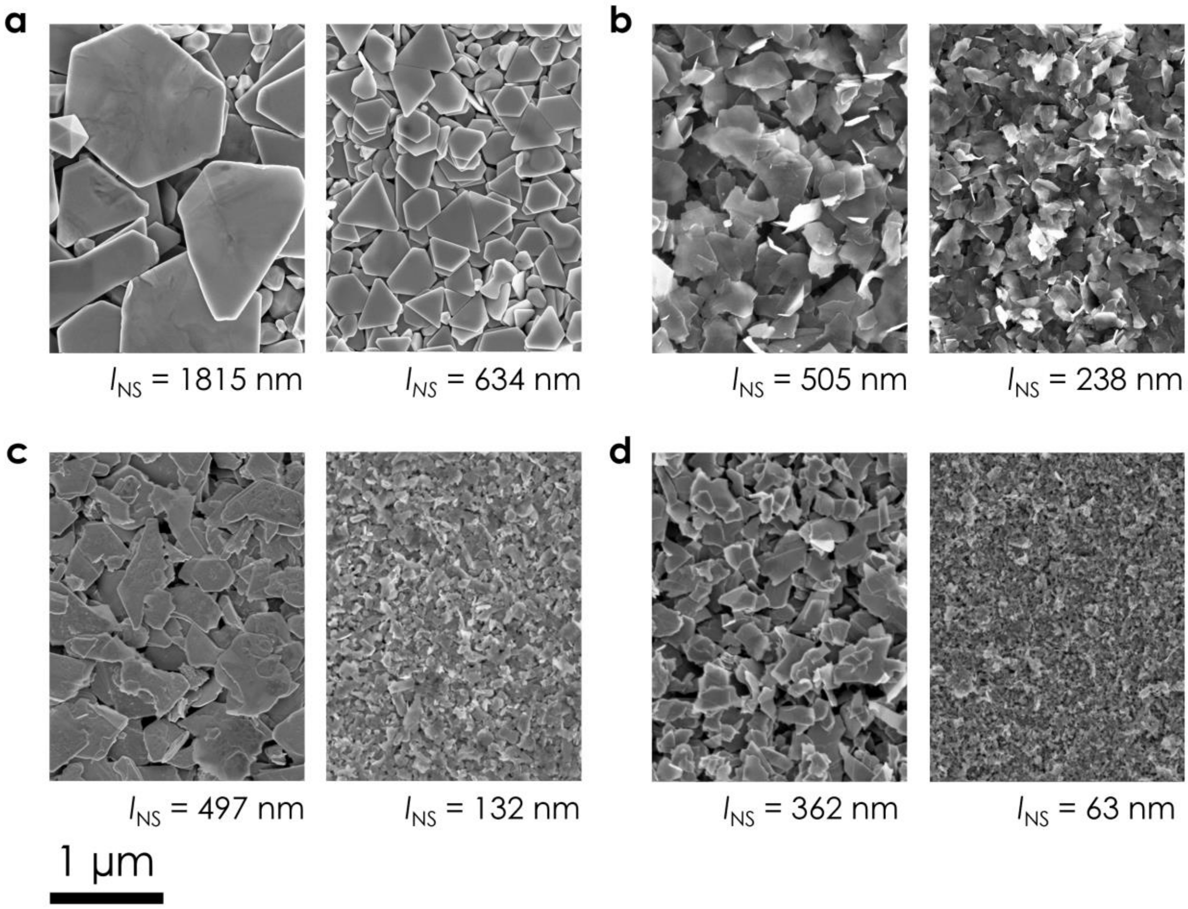

Supplementary figure 16: SEM characterisation of printed nanosheet networks. High-resolution surface SEM images (Inlens detector) of spray-cast **(a)** AgNSs, **(b)** graphene, **(c)** WS_2 and **(d)** WSe_2 networks. Two networks, each with a different nanosheet length, l_{NS}, are shown at the same scale for each material.

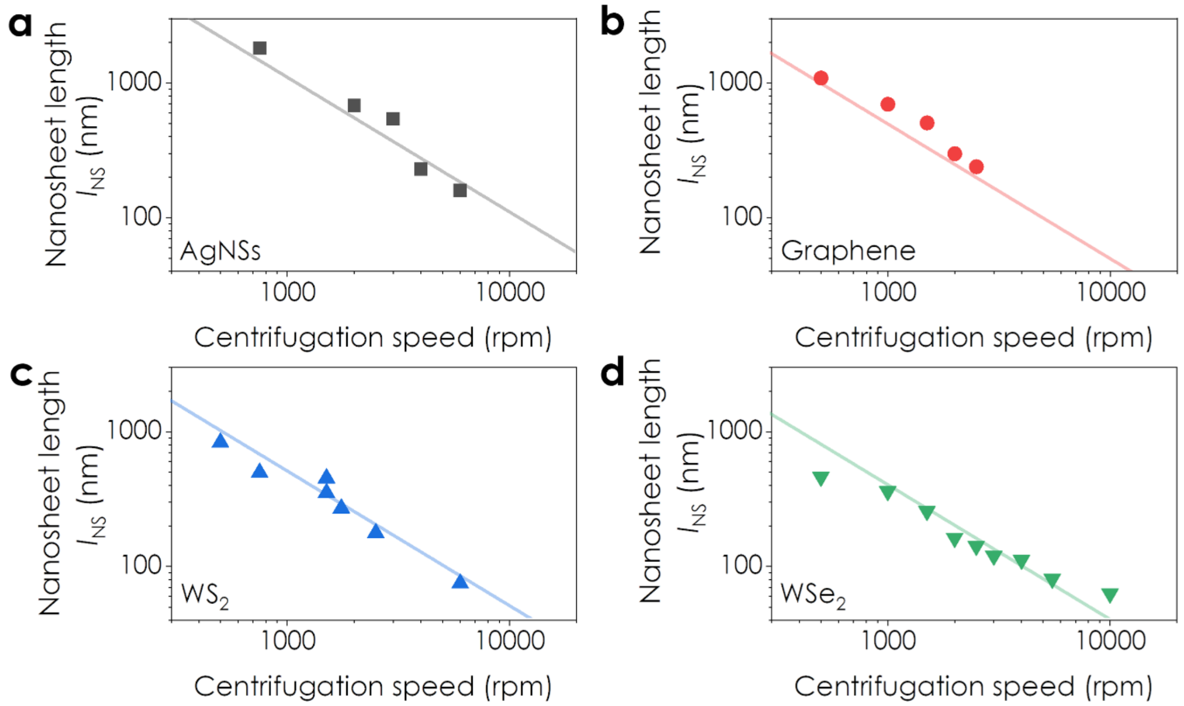

Supplementary figure 17: Nanosheet length as a function of centrifugation speed. Mean nanosheet length for various fractions plotted versus the centrifugation speed used to collect nanosheets as sediment for **(a)** AgNSs, **(b)** graphene, **(c)** WS_2 and **(d)** WSe_2. The solid lines represent the usually observed rpm dependence of $(rpm)^{-1}$. The data are presented as means ± standard error (SE) in the mean for the AgNSs (n = 135 - 251), graphene (n = 210 - 270), WS_2 (n = 226 - 443) and WSe_2 (n = 89 - 227).

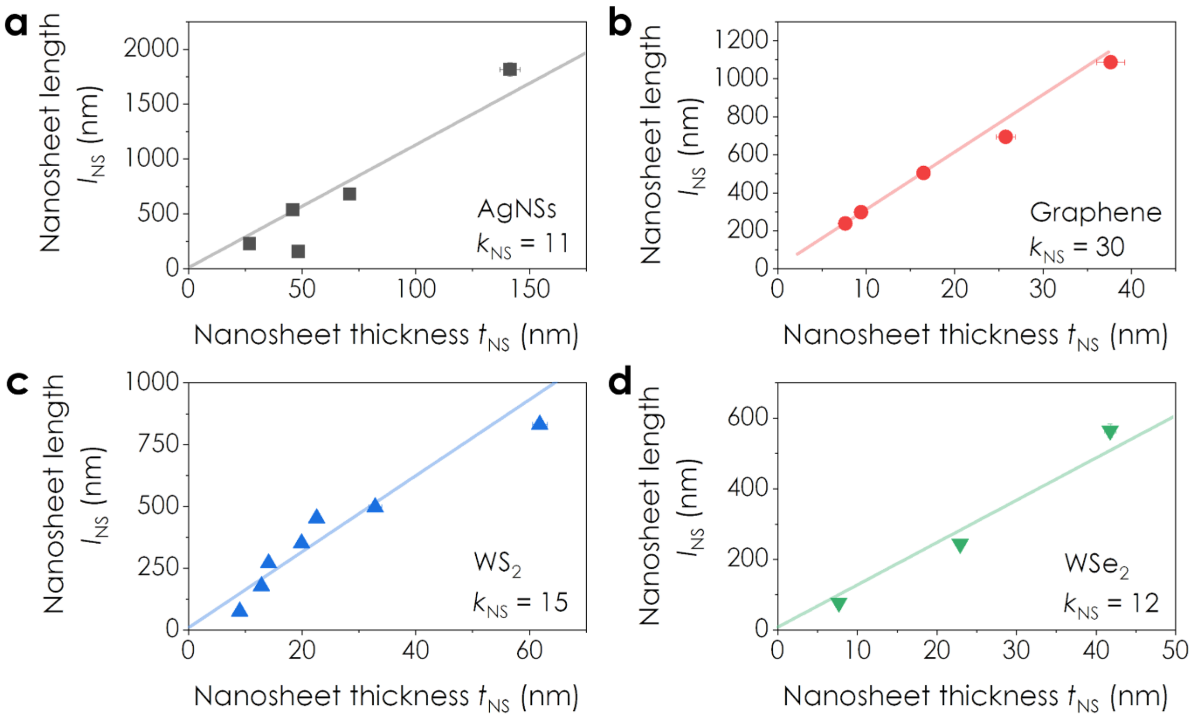

Supplementary figure 18: Length-thickness relationship for LPE nanosheets. Mean nanosheet length, l_{NS}, for various fractions plotted versus mean nanosheet thickness, t_{NS}, for **(a)** AgNSs, **(b)** graphene, **(c)** WS_2 and **(d)** WSe_2. The lines represented a linear relationship. The mean nanosheet aspect ratios, k_{NS}, are given for each material in the panels. The data are presented as means ± SE in the mean for the AgNSs (n = 135 - 251), graphene (n = 210 - 270), WS_2 (n = 226 - 443) and WSe_2 (n = 89 - 227).

2.7. Electrochemically exfoliated (EE) MoS_2

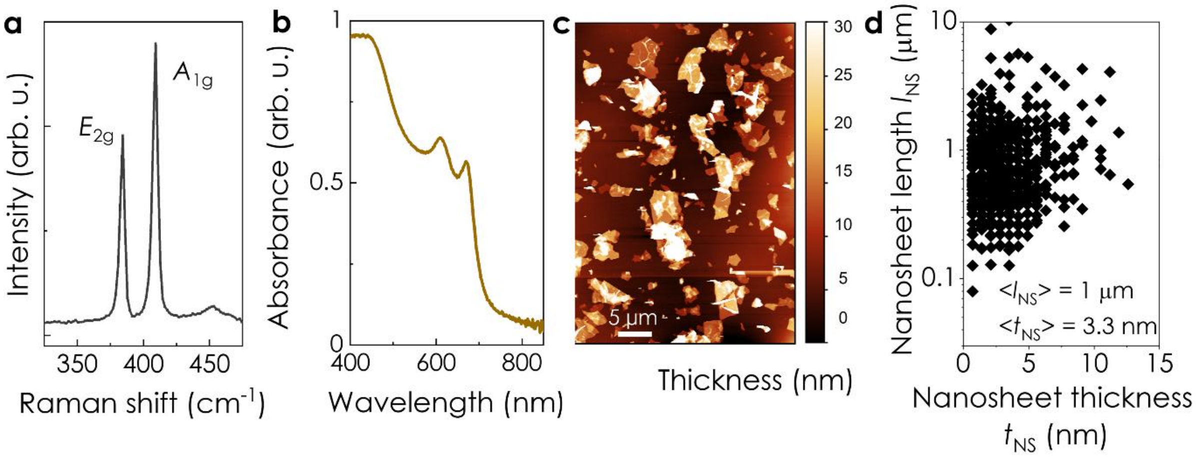

Supplementary figure 19: Basic characterisation of MoS_2 nanosheets produced by electrochemical exfoliation. Nanosheets were prepared and characterised in an identical manner to ref[16]. **(a)** Raman spectrum (532 nm) measured on a drop cast film. **(b)** Optical absorbance spectrum with the scattering contribution removed[17]. **(c)** Representative AFM image of the EE MoS_2 nanosheets. **(d)** AFM data for nanosheet length, l_{NS}, and thickness, t_{NS}. Here the apparent thickness was converted to layer number by dividing by 1.9 nm per monolayer[18] and then converted to real thickness assuming 0.7 nm per layer. See ref [16] for more details.

Supplementary note 3. Fitting DC resistivity data

Fitting methodology: When fitting the WSe_2 and WSe_2 semiconductor data with Eq. (3) in the main text (also Eqn. S2b), we generally replaced the nanosheet resistivity, ρ_{NS}, by $(en_{NS}\mu_{NS})^{-1}$. This does not change the number of fit parameters but replaces resistivity with mobility. This is useful as values for the nanoparticle mobility are generally known from the literature.

Given the number of parameters in Eq. (2) and Eq. (3) (main text), or Eqns. S2, S3 and S4 here, it is necessary to fix some of the parameters. For each of the fits shown in Fig. 3 in the main text, the fit parameters are given in this table. Fixed parameters are in bold.

	μ_{NP} (cm^2 V^{-1} s^{-1})	n_{NP} (m^{-3})	σ_{NP} (S m^{-1})	ρ_{NP} (Ω m)	k_{NS} or D_{NW}	R_J (Ω)	P_{Net}
Graphene	**2000**	$(1.8\pm0.6)\times10^{24}$	$(5.8\pm1.9)\times10^{4}$	$(1.7\pm0.5)\times10^{-5}$	**30**	3275±314	**0.45**
AgNSs	**-**	-	$(1.4\pm0.7)\times10^{7}$	$(7.2\pm3.9)\times10^{-8}$	**11**	5.2±0.7	**0.35**
WS_2	**60**	$(5.4\pm0.9)\times10^{20}$	0.52±0.08	1.9±0.3	**15**	$(2.4\pm0.23)\times10^{10}$	**0.5**
WSe_2	**90**	$(2.0\pm0.4)\times10^{21}$	2.9±0.6	0.35±0.07	**12**	$(6.1\pm0.7)\times10^{9}$	**0.5**
AgNWs	-	-	$(6.6\pm0.9)\times10^{7}$	$(1.5\pm0.2)\times10^{-8}$	**39 nm**	254±8	**0.85**

Supplementary table 1: Parameters used when fitting the DC network resistivity versus nanoparticle length. Fixed parameters are in bold. All other values were extracted from fits to Eq. (2) (AgNWs) and Eq. (3) (AgNSs, graphene, WS_2 and WSe_2). Nanosheet aspect ratio, k_{NS}, and AgNW diameter, D_{NW}, were measured using AFM and SEM respectively. Network porosity, P_{Net}, values were taken from Gabbett et al.[9]. WS_2 and WSe_2 mobility values were extracted from Kelly et al.[19], while the mobility of graphene was taken as the in-plane mobility of graphite[20]. Values for the nanoparticle resistivity, ρ_{NP}, and conductivity, σ_{NP}, were calculated from nanoparticle mobility, μ_{NP}, and carrier density, n_{NP}, values using $\sigma_{NP}^{-1} = \rho_{NP} = (n_{NP}e\mu_{NP})^{-1}$. For AgNWs and AgNSs, n_{NP} is very large, allowing the second square-bracketed terms in Eqs. (2) and (3) to be neglected. Then a simple linear fit can be used yielding ρ_{NP} and R_J, once P_{Net} and k_{NS} (or D_{NW}) are known.

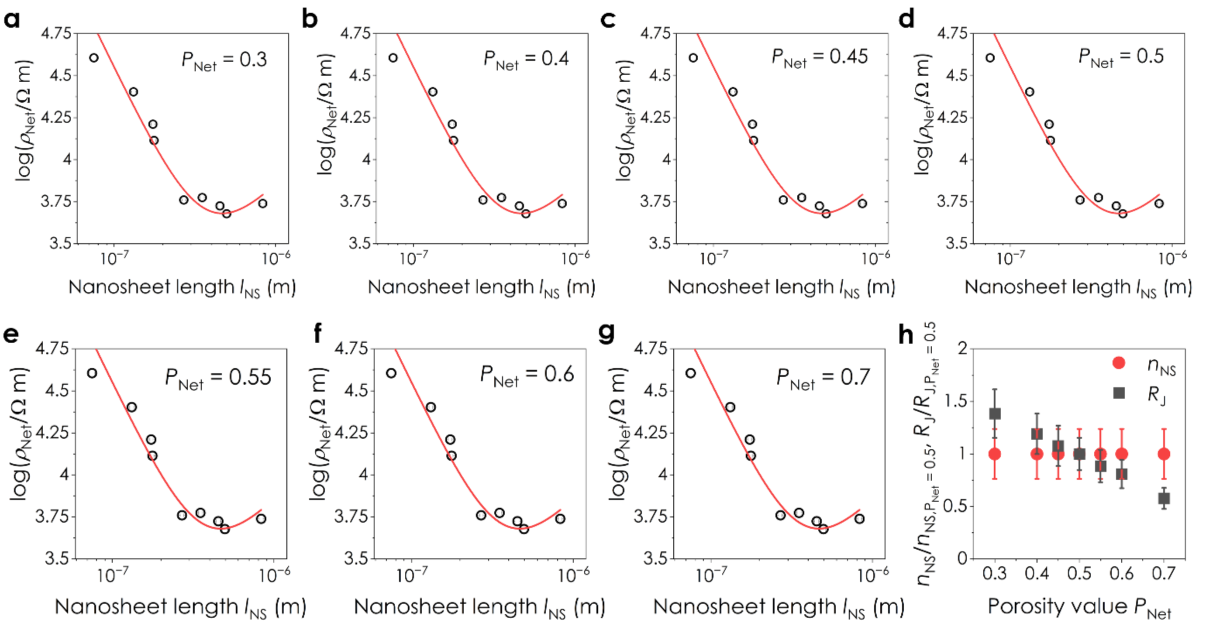

Supplementary figure 20: Fitting sensitivity analysis for network resistivity versus nanosheet length data measured on WS_2. We fit the data using Eq. (3) in the main text. During fitting, $\mu_{NS} = 60$ cm^2 V^{-1} s^{-1} and $k_{NS} = 15$ were fixed and the parameters n_{NS} and R_J were allowed to vary. **(a-g)** Different fits to the same data with the network porosity, P_{Net}, fixed at **(a)** 0.3, **(b)** 0.4, **(c)** 0.45, **(d)** 0.5, **(e)** 0.55, **(f)** 0.6 and **(g)** 0.7. **(h)** The resultant values of n_{NS} and R_J, normalised to their values found for the P_{Net} = 0.5 fit ($n_{Net,P_{Net}=0.5}$ and $R_{J,P_{Net}=0.5}$). This analysis shows n_{NS} to be extremely insensitive to the value of P_{Net} used in fitting. R_J was also relatively insensitive, showing a variation of ±40% as P_{Net} was varied from 0.3 to 0.7. The uncertainties in n_{NS} and R_J are the errors in the fits to Eq. (3) (main text) for the data shown in **(a-g)**.

Ruling out other effects: It is worth considering whether the processing regime could result in variations in nanosheet properties among the size-selected fractions. This includes differences in defect content that might contribute to any dependence of network conductivity on nanosheet size. In general, there is much evidence that sonication of 2D materials during the liquid phase exfoliation process introduces only minimal numbers of basal plane defects. For example, Raman measurements on size-selected liquid phase exfoliated graphene have shown the ratio of defect to graphitic band intensity (D:G) to scale perfectly linearly with inverse nanosheet size, with an intercept that is very similar to the D:G ratio for the starting graphite[21]. This implies that the defects seen in Raman are predominately edge defects and those basal plane defects that do exist are almost all from the starting graphite (i.e. not created by sonication). In addition, more detailed Raman studies[22] on similar samples, comparing D:G ratios with G-linewidths, concludes that LPE nanosheets have a relatively low basal plane defect density. It is also worth mentioning that sonication-exfoliated WS_2 nanosheets show

very narrow photoluminescence (PL) linewidths (~30 meV)[15], comparable to those found in mechanically exfoliated samples. As PL spectra tend to broaden in the presence of defects, this is a strong indicator that sonication is not adding large quantities of defects. Finally, PL measurements on liquid phase exfoliated MoS_2 nanosheets (exfoliated and size separated in a manner very similar to the graphene, WS_2 and WSe_2 used here) show that across several size-selected fractions, the PL intensity scales linearly with the population of monolayers in each fraction, indicating that there is no intrinsic difference between the nanosheets of different sizes (i.e. there is no observable difference in defect content among the different nanosheet sizes)[23].

Taken together, these observations imply that relatively few basal plane defects are created by sonication during liquid phase exfoliation and that there should be no significant difference between the defect content of size-selected fractions. This rules out induced defects as the source of the observed dependence of network resistivity on nanosheet size.

Supplementary note 4. Impedance basics

Impedance spectroscopy is an extremely powerful tool for extracting a vast quantity of information from a relatively quick measurement. However, working at high frequencies means a significant amount of consideration must go into both sample preparation and the measurement setup. The specific conditions of the sample, whether it is liquid or solid for example, will determine the optimal measuring environment. Impedance spectroscopy has yet to be performed in-depth on nanosheet networks so the aim of Supplementary notes 4-6 and 8-12 is to provide the reader with a sufficient basis to reproduce and build upon the work presented herein without the trial-and-error associated with a new technique.

Basic Introduction to Impedance Spectroscopy

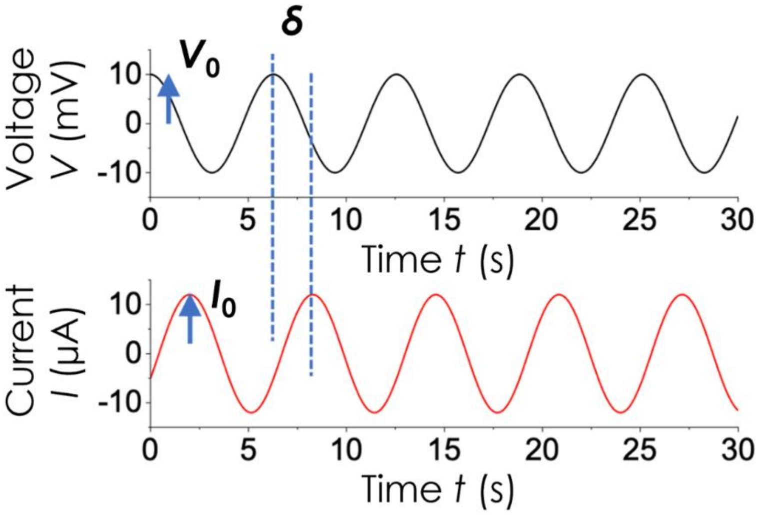

Supplementary figure 21: Impedance spectroscopy. Applied sinusoidal voltage with amplitude, V_0, and measured current, I_0, with a phase shift of δ.

In electrical impedance spectroscopy, a small sinusoidal voltage is applied to a sample at a given amplitude, V_0, and frequency, ω, as shown in Suppl. Fig. 21 such that

$$V(\omega) = V_0 e^{i\omega t}$$

The resulting current it produces is then phase-shifted by an amount, δ, such that

$$I(\omega) = I_0 e^{i(\omega t - \delta)}$$

This measurement is performed over a wide range of frequencies to produce an impedance spectrum. Following Ohm's Law, the impedance is given by

$$Z = \frac{V}{I} = \frac{V_0 e^{i\omega t}}{I_0 e^{i(\omega t - \delta)}} = \frac{V_0}{I_0} e^{i\delta}$$

By de Moivre's theorem, this is divided into real, Re(Z), and imaginary, Im(Z), components such that

$$Z = \frac{V_0}{I_0}\cos(\delta) + i\frac{V_0}{I_0}\sin(\delta)$$

which is usually written in terms of the modulus, $|Z|$, as

$$Z = |Z|\cos(\delta) + i|Z|\sin(\delta) = \mathrm{Re}(Z) + \mathrm{Im}(Z)$$

The directly measured variables are $|Z|$ and δ which can be converted to Re(Z) and Im(Z) using the relation above.

We note that Re(Z) and Im(Z) are also commonly written as Z' and Z'' respectively.

In practice, impedance spectroscopy involves measuring Re(Z) and Im(Z) as a function of angular frequency, ω, and analysing this data, usually by comparison to hypothesised equivalent circuits. For a more comprehensive description, see ref[24].

Supplementary note 5. Conversion of Z_{Net} to $Z_{NS\text{-}J}$: Mathematics

In Supplementary note 1 we derived an equation for the network conductivity which can be converted to represent the network resistivity, ρ_{Net}, for nanosheet networks as

$$\rho_{Net} \approx \frac{2t_{NS}}{(1-P_{Net})}\left[R_{NS}+R_{J}\right]\left[1+\frac{2}{n_{NS}t_{NS}l_{NS}^{2}}\right]$$

where P_{Net} is the network porosity, n_{NS}, t_{NS} and l_{NS} are the nanosheet carrier density, thickness and length. All values should be considered averages over the network. The mean resistance of a single (average) nanosheet-junction combination (R_{NS} + R_{J}) can be more generally represented by the equivalent impedance of the nanosheet-junction pair, $Z_{NS\text{-}J}$. In addition, the network impedance is related to the network resistivity via $Z_{Net} = \rho_{Net}L_{Ch} / A_{Net}$, where L_{Ch} is the channel length and A_{Net} is the cross-sectional area of the network. In the case of a 2D network, this is

$$Z_{Net} \approx \frac{L_{Ch}}{A_{Net}}\frac{2t_{NS}\kappa_{Net}}{(1-P_{Net})}Z_{NS\text{-}J}\left[1+\frac{2}{n_{NS}t_{NS}l_{NS}^{2}}\right]$$

This equation can be used to convert the real and imaginary network impedance spectra (i.e. as a function of angular frequency, ω, to spectra of the real and imaginary components of $Z_{NS\text{-}J}$ such that

$$Z_{NS\text{-}J} = \frac{A_{Net}}{L_{Ch}}\frac{(1-P_{Net})}{2t_{NS}\kappa_{Net}}Z_{Net}\left[1+\frac{2}{n_{NS}t_{NS}l_{NS}^{2}}\right]^{-1}$$

where the real or imaginary components are substituted for Z_{Net}. In terms of network resistivity, this becomes

$$Z_{NS\text{-}J} = \rho_{Net}^{*}\frac{(1-P_{Net})}{2t_{NS}\kappa_{Net}}\left[1+\frac{2}{n_{NS}t_{NS}l_{NS}^{2}}\right]^{-1} \qquad \text{Equation S7}$$

In the case of conductive nanosheets such as graphene and AgNSs, the square bracketed term will tend to 1 leading to:

$$Z_{NS\text{-}J} \approx \frac{A_{Net}(1-P_{Net})}{2L_{Ch}t_{NS}\kappa_{Net}}Z_{Net}$$

In the case of a 1D network

$$Z_{\mathrm{Net}} = Z_{\mathrm{NW\text{-}J}} \frac{L_{\mathrm{Ch}}}{A_{\mathrm{Net}}} \frac{\pi D_{\mathrm{NW}}^2 \kappa_{\mathrm{Net}}}{2l_{\mathrm{NW}}(1-P_{\mathrm{Net}})} \left[1+\frac{8}{n_{\mathrm{NW}} l_{\mathrm{NW}} \pi D_{\mathrm{NW}}^2}\right]$$

Therefore

$$Z_{\mathrm{NW\text{-}J}} = Z_{\mathrm{Net}} \frac{A_{\mathrm{Net}}}{L_{\mathrm{Ch}}} \frac{2l_{\mathrm{NW}}(1-P_{\mathrm{Net}})}{\pi D_{\mathrm{NW}}^2 \kappa_{\mathrm{Net}}} \left[1+\frac{8}{n_{\mathrm{NW}} l_{\mathrm{NW}} \pi D_{\mathrm{NW}}^2}\right]^{-1}$$

Again, for conductors this becomes

$$Z_{\mathrm{NW\text{-}J}} = Z_{\mathrm{Net}} \frac{A_{\mathrm{Net}}}{L_{\mathrm{Ch}}} \frac{2l_{\mathrm{NW}}(1-P_{\mathrm{Net}})}{\pi D_{\mathrm{NW}}^2 \kappa_{\mathrm{Net}}}$$

This implies that the network impedance must be multiplied by a numerical value (defined by Eqn. S7 for nanosheets), to obtain the impedance of a nanosheet-junction pair. We will discuss this conceptually in Supplementary note 6.

Supplementary note 6. Conversion of Z_{Net} to $Z_{NS\text{-}J}$: Concepts and an example

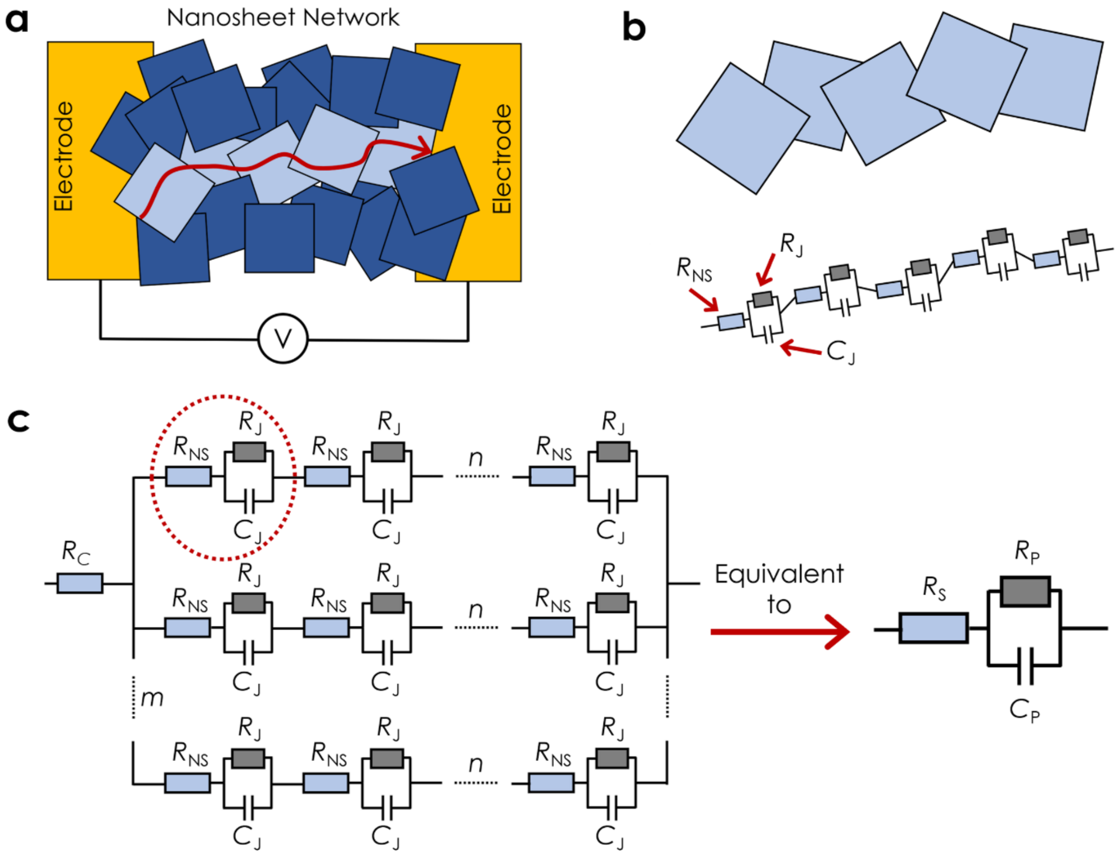

Supplementary figure 22: Conversion of Z_{Net} to $Z_{NS\text{-}J}$. (a) Schematic of a nanosheet network between two electrodes under an applied voltage, *V*. Current flows through many parallel current paths (red arrow). **(b)** Such a path is a linear chain of nanosheet-junction pairs. Each nanosheet-junction pair in the chain can be thought of a combination of a resistor representing the nanosheet (R_{NS}, blue) and a parallel combination of resistor (R_J, grey) and capacitor (C_J) representing the junction. **(c)** Schematic showing a nanosheet network to consist of an array of circuit elements, each representing individual nanosheet-junction pairs (red dashed circle). In this representation, there are *m* parallel current paths, each containing *n* NS-J elements in series. The overall electrode-network contact resistance, R_C, is also shown. This is equivalent to the Randles circuit on the right, which comprises a series resistor, R_S, and a parallel combination of a resistor, R_P, and a capacitor, C_J.

Impedance measurements of a nanosheet network (Z_{Net}) probe a very large number of nanosheet-junction (NS-J) pairs (Suppl. Fig. 22a). We can think of these NS-J pairs as arranged in linear current paths (Suppl. Fig. 22b). Each NS-J pair is represented by the circuit element within the red dashed circle in Suppl. Fig. 22c. The NS-J circuit element has impedance $Z_{NS\text{-}J}$. We can think of a network as a large array of NS-J circuit elements as shown in Suppl. Fig. 22c (schematic also includes the electrode-network contact resistance, R_C). In this simplest

case, we represent the network as consisting of many (say, m) parallel conductive paths, each one containing a number (say, n) of nanosheet junction pairs in series.

In this simplest case and where all nanosheet-junction pairs are the same, the impedance of the array representing the network is then $Z_{\mathrm{Net}} = \frac{n}{m} Z_{\mathrm{NS\text{-}J}}$. Although this is the simplest possible case, it shows that we expect $Z_{\mathrm{NS\text{-}J}}$ to be related to Z_{Net} by a multiplicative factor in all cases. For real networks, this multiplicative factor is given by Eqn. S7.

This multiplicative relationship means that the array representing the network can be represented by a simple Randles circuit of the same form as the NS-J circuit element (Suppl. Fig. S22c). Here, in the simplest case, we expect $R_{\mathrm{P}} = R_{\mathrm{J}} n/m$, while R_{S} has contributions from both nanosheets and the contact resistance such that $R_{\mathrm{S}} = R_{\mathrm{C}} + R_{\mathrm{NS}} n/m$. If the contact resistance is minimised, we have $R_{\mathrm{S}} \approx R_{\mathrm{NS}} n/m$. The network impedance can be converted to the NS-J impedance using Eqn. S7, which can be written as

$$Z_{\mathrm{NS\text{-}J}} = Z_{\mathrm{Net}} \frac{A_{\mathrm{Net}}}{L_{\mathrm{Ch}}} \frac{(1-P_{\mathrm{Net}})}{2t_{\mathrm{NS}}\kappa_{\mathrm{Net}}} \left[1 + \frac{2}{n_{\mathrm{NS}} t_{\mathrm{NS}} l_{\mathrm{NS}}^2}\right]^{-1} = \rho_{\mathrm{Net}}^{*} \frac{(1-P_{\mathrm{Net}})}{2t_{\mathrm{NS}}\kappa_{\mathrm{Net}}} \left[1 + \frac{2}{n_{\mathrm{NS}} t_{\mathrm{NS}} l_{\mathrm{NS}}^2}\right]^{-1}$$

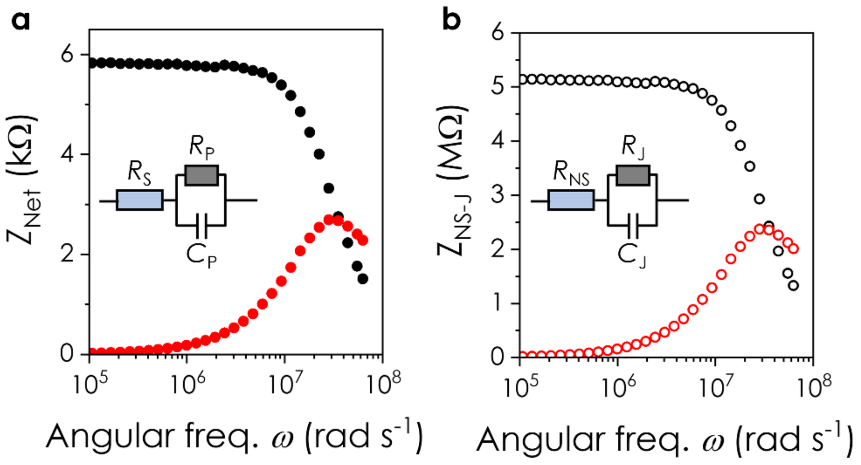

Supplementary figure 23: Converting the network impedance, Z_{Net}, to the impedance of a nanosheet-junction pair, $Z_{\mathrm{NS\text{-}J}}$. (a) The real (black) and imaginary (red) impedance spectra for a network of electrochemically exfoliated MoS_2 nanosheets. **(b)** The same spectra after conversion to represent a single nanosheet-junction pair. This conversion was performed using W_{Ch} = 19.4 mm, t = 15 nm, L_{Ch} = 50 µm, t_{NS} = 3.3 nm, l_{NS} = 1 µm, κ_{Net} = 1, P_{Net} = 0, n_{NS} = 4×10^{23} m^{-3}. For both sets of spectra, the equivalent circuits are shown as insets.

To convert the network impedance spectra to that of a nanosheet-junction pair as shown in Supplementary figure 23, the following parameters must be known: the channel length, L_{Ch},

the cross sectional area, A_{Net} (=$W_{Net}\times t$, where W_{Net} is the channel width and t is the network thickness); the mean nanosheet thickness, t_{NS}, and mean nanosheet length, l_{NS} (the latter two are typically measured by AFM[12]). The in-plane tortuosity factor, κ_{Net}, is very close to 1 for electrochemically exfoliated nanosheets and may be neglected[9]. Likewise, for electrochemically exfoliated networks produced by Langmuir-Schaefer depsoition, the porosity, P_{Net}, is very small and may be neglected.

One issue is that the carrier density, n_{NS}, must be known for the conversion, unless $n_{NS} >> (t_{NS} l_{NS}^2 / 2)^{-1}$ which typically occurs for $n_{NS}>>10^{21}$ m^{-3} for EE nanosheets. Typically, n_{NS} is measured via field-effect mobility measurements on the network. The resultant μ_{Net} values are then combined with the measured network conductivity to get n_{NS} (by taking the approximation that $n_{NS} \approx n_{Net}$). We note that converting Z_{Net} to $Z_{NS\text{-}J}$ involves multiplying the real and imaginary impedance spectra of the network by the same multiplicative factor. As a result, the vertical scale of the spectra changes but not their shape. For example, the peak in the Im(Z) spectra in Suppl. Fig. 23 is at the same angular frequency in each case. For Randles circuits, this peak frequency is given by the inverse product of the parallel resistance and capacitances such that $\omega_{peak} = \left(R_P C_P\right)^{-1} = \left(R_J C_J\right)^{-1}$

Since the simplest case predicts $R_P = R_J n / m$, this implies $C_P = C_J m / n$ as expected given the laws for combining series and parallel capacitors.

It is worth noting that above, we referred to a method to relate Z_{Net} to $Z_{NS\text{-}J}$ (Eqn. S7) and a "simplest case" where $Z_{Net} = Z_{NS\text{-}J} n / m$. These can be inter-related. The parameter n is effectively the number of nanosheet-junction pairs in a current path such that $n = \kappa_{Net} L_{Ch} / (l_{NS} / 2)$, where κ_{Net} accounts for the fact that the path is probably not straight and the factor of 2 is to account for the fact that on average each charge goes through only half the nanosheet length. The parameter m is the number of current paths in parallel such that $m = A_{Net}(l_{NS} t_{NS} / (1-P_{Net}))^{-1}$ where the factor $(1-P_{Net})$ accounts for the free space between nanosheets. Combining gives

$$Z_{NS\text{-}J} = Z_{Net}\frac{m}{n} = Z_{Net}\frac{A_{Net} / \left[l_{NS} t_{NS} / (1-P_{Net})\right]}{\kappa_{Net} L_{Ch} / (l_{NS} / 2)} = Z_{Net}\frac{A_{Net}}{L_{Ch}}\frac{(1-P_{Net})}{2\kappa_{Net} t_{NS}}$$

This is very similar to Eqn. S7, although it is missing the final term which is needed to properly account for the effect of voltage drops across both nanosheet and junction.

Supplementary note 7. Transistor measurements on a nanosheet network

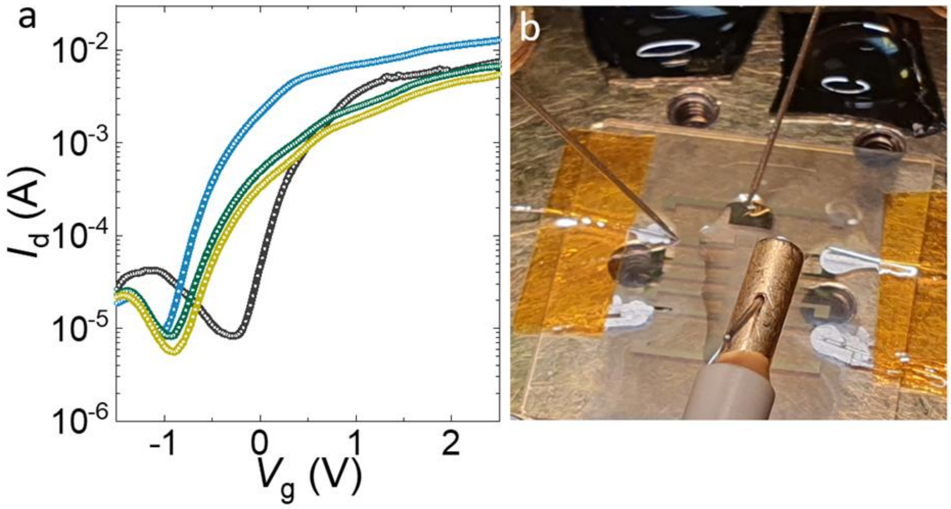

Supplementary figure 24: TFT measurements on a 1-LS-layer MoS_2 network. (a) Transfer characteristic of the MoS_2 transistors where drain current, I_D, is measured as a function of the applied gate voltage, V_g. **(b)** Experimental setup of the electrochemically gated devices showing EMIM-TFSI which has been drop-casted onto the surface of the MoS_2 networks. Gold-coated probe tips are also shown in contact with the source, drain and gate electrodes.

The mobility is extracted following a procedure previously reported[16]. In that paper, the areal capacity for a 25 nm thick film of the same EE MoS_2 nanosheets and same electrolyte (EMIM-TFSI) as used here was found (at the appropriate scan rate) to be 3.1 µF cm^{-2}. Correcting for the fact that in the current work, the film thickness was 15 nm yields a capacitance value of 2 µF cm^{-2} is used for our transistors. Averaging over four transistors find a peak mobility of 6.6 ± 0.6 cm^2 V^{-1} s^{-1} with an on/off ratio of $\approx 10^3$.

Supplementary note 8. Measuring impedance: Substrate capacitance

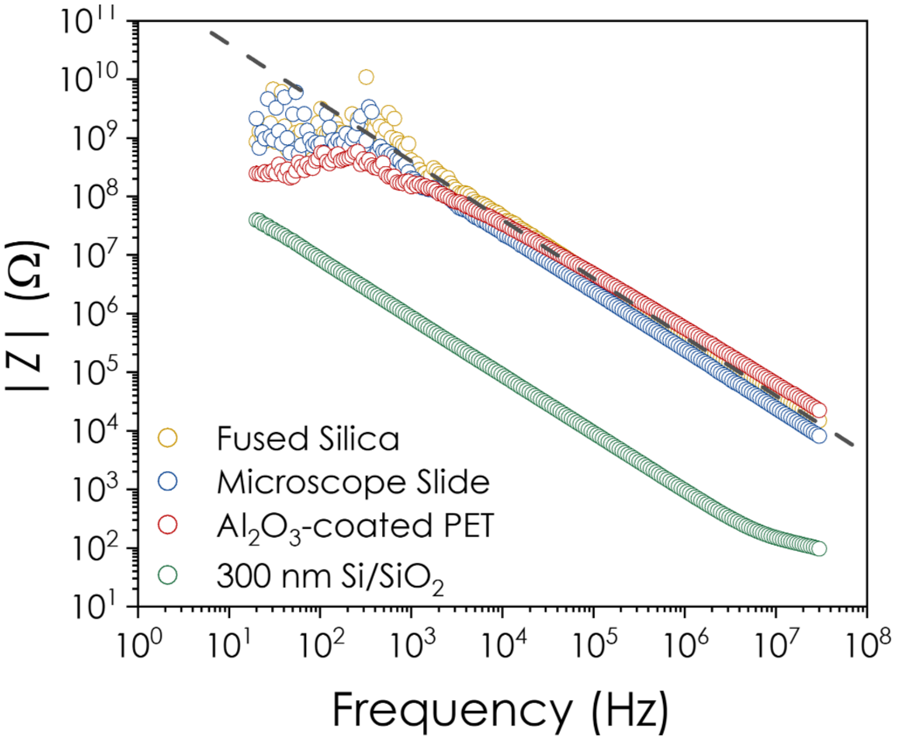

Supplementary figure 25: Background impedance spectra for different substrates. Impedance spectra for bare Ti:Au electrodes (L_{Ch} = 50 µm, W_{Ch} = 19.4 mm) on various substrates including fused silica, a glass microscope slide, Al_2O_3-coated PET and a silicon wafer (300 nm thick SiO_2 layer).

Suppl. Fig. 25 shows room temperature impedance spectra measured for Ti/Au electrodes, but no sample, on various substrates. These electrodes are identical to those used in Fig. 4 (main text) and were evaporated onto each of the substrates indicated in the legend. The dashed line is a plot of a capacitance of 3×10^{-13} F which is the capacitance for this electrode geometry with a fused silica substrate. The fused silica substrate behaves close to this ideal behaviour, as does the microscope slide. We note the Al_2O_3-coated PET appears to show a deviation away from the line of purely capacitive behaviour at low frequency, which is typically indicative of a parallel resistor. This may be caused by a small leakage current flowing through the substrate. The Si/SiO_2 substate shows a much reduced background impedance. This is probably due to coupling of the electrodes to the resistance of the Si via capacitances associated with metal/SiO_2/Si. This makes Si/SiO_2 substates compeltely unsuitable for impedance work.

Supplementary note 9. Measuring impedance: Influence of substrate capacitance

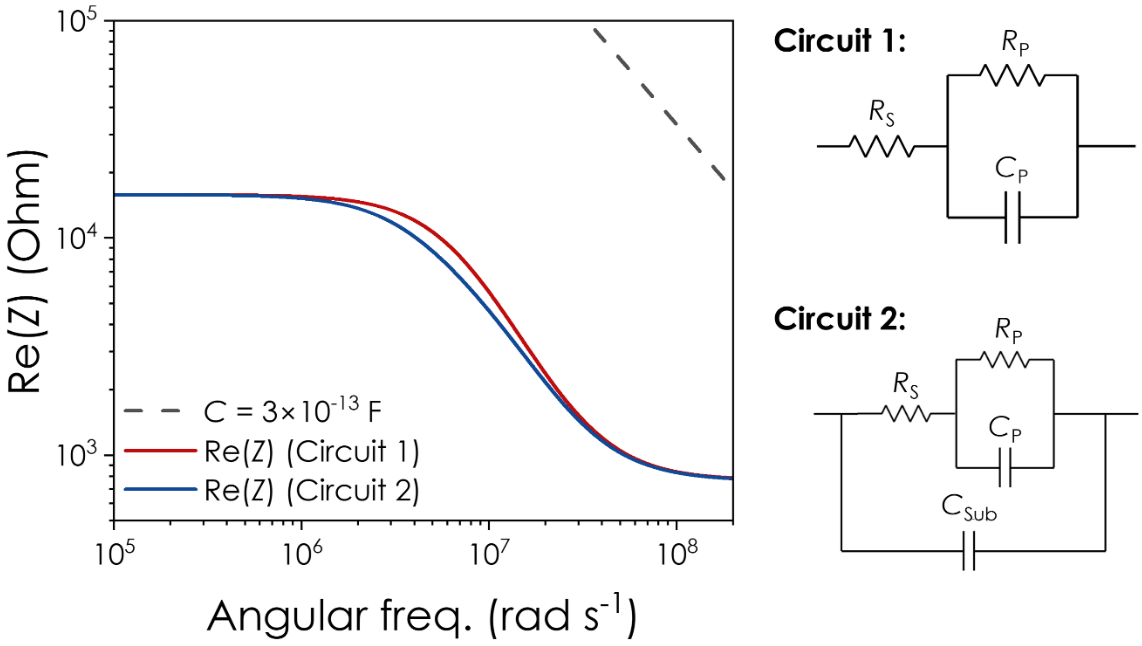

Supplementary figure 26: Effect of substrate capacitance on the Re(*Z*) spectrum. Real component of the impedance spectrum, Re(*Z*), plotted for the two circuit types shown using data extracted from an EE MoS_2 network. These show typical Randles circuits (such as those measured for networks) with series resistance, R_S, and parallel resistance and capacitance, R_P and C_P (Circuit 1). Circuit 2 also includes a substrate capacitance, C_{Sub}, in parallel.

The substrate in these systems can act as a parallel capacitor to the nanosheet network and its effect on fitting therefore needs to be evaluated in the case it impacts the value of parameters extracted during equivalent circuit (Suppl. Fig. 26) analysis, such as R_S. In Suppl. Fig. 26, we model a circuit using data extracted from a measured MoS_2 network (R_S = 767 Ohm, R_P = 15,061 Ohm and $C_P = 9.6\times10^{-12}$ F) in two forms: Circuit 1, which is a simple Randles circuit (i.e. $n = 1$ in Eq. (5) in the main text) representing the nanosheet network, and Circuit 2, which is a simple Randles in parallel with a capacitor representing the substrate. The dashed line indicates the background capacitance of the fused silica substrate. The real part of the impedance for Circuit 1 is given by

$$\mathrm{Re}(Z) = R_S + \frac{R_P}{1 + (\omega R_P C_P)^2}$$

And for Circuit 2 is given by

$$\mathrm{Re}(Z) = \frac{R_{\mathrm{S}} + \dfrac{R_P}{1+(\omega R_{\mathrm{P}} C_{\mathrm{P}})^2}}{\left(\omega C_{\mathrm{Sub}} R_{\mathrm{S}} + \dfrac{\omega C_{\mathrm{Sub}} R_{\mathrm{P}}}{1+(\omega R_{\mathrm{P}} C_{\mathrm{P}})^2}\right)^2 + \left(\dfrac{\omega^2 C_{\mathrm{Sub}} R_{\mathrm{P}}^2 C_{\mathrm{P}}}{1+(\omega R_{\mathrm{P}} C_{\mathrm{P}})^2} + 1\right)^2}$$

By plotting these two equations with the values above, we see that the value of the high and low frequency plateaus are unaffected by the substrate capacitance (Suppl. Fig. 26). This means fit values of R_{S} and R_{P} are unaffected by such values of the substrate capacitance. However, the substrate capacitance may induce a small systematic error in the parallel capacitance, C_{P}.

Supplementary note 10. Measuring impedance: Background artefacts

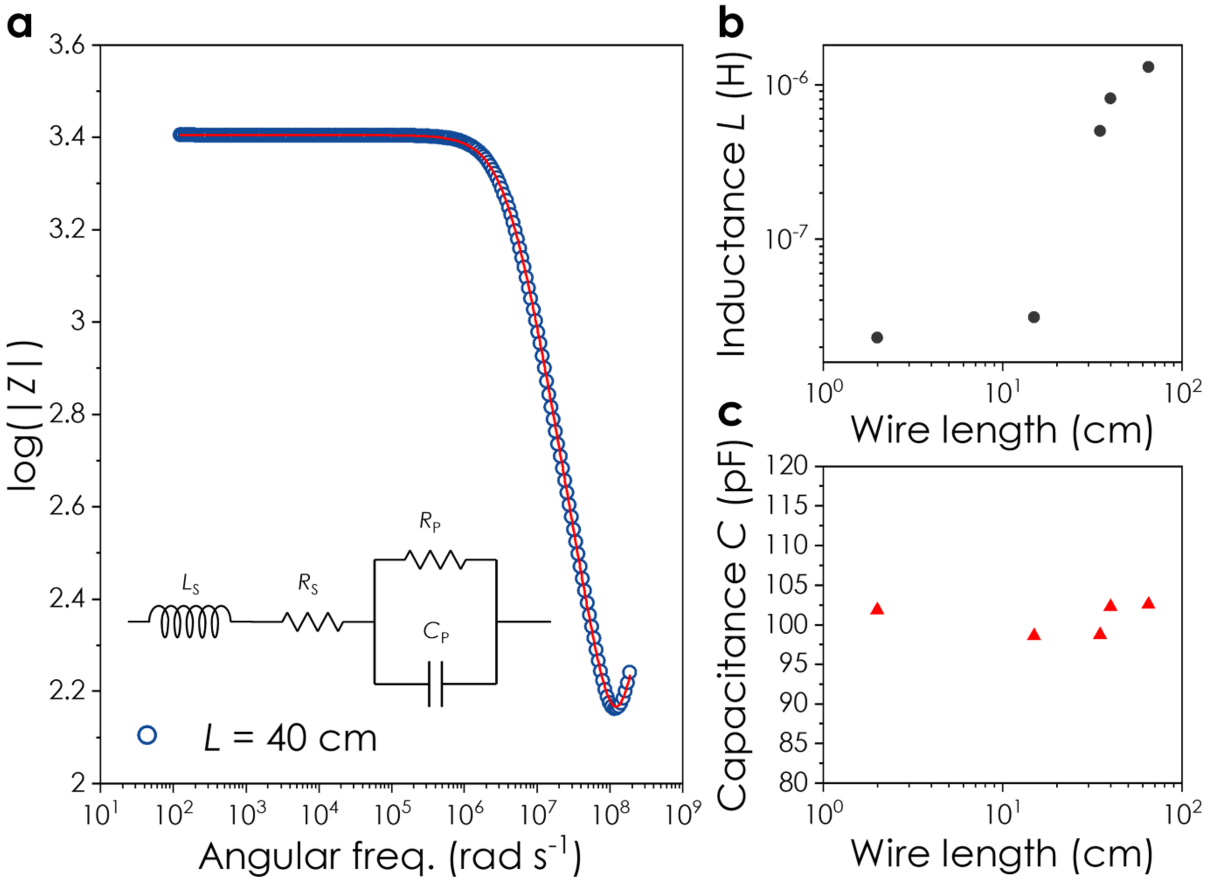

Supplementary figure 27: Estimation of the effect of wiring on the background impedance spectrum. (a) Representative impedance spectrum from a four-layer MoS_2 device with a 40 cm wire length. **(b)** Measured inductance, *L*, as a function of wire length. **(c)** Measured parallel capacitance as a function of wire length.

The extraction of parameters, especially R_S, relies on the high frequency end of the spectrum containing contributions from only the Randles circuit (i.e. the nanosheet network). However, this part of the spectrum is also highly sensitive to inductive artefacts associated with the measurement setup such as the wiring. To assess this, wires of various lengths were attached from a four-layer MoS_2 device to the impedance analyser to determine the role of wire length in the inductance of the impedance spectra. Suppl. Fig. 27a shows a representative spectrum for a 40 cm wire length where a clear upturn at high frequency is caused by the inductance between the wires. These spectra were fit to the circuit inset in Suppl. Fig. 27a using the imperfect capacitor model which is given by the equation

$$|Z|^2 = \left[R_S + R_P \left(1 + \frac{(\omega R_P C_P)^n \cos\left(\frac{n\pi}{2}\right)}{1 + (\omega R_P C_P)^{2n} + 2\cos\left(\frac{n\pi}{2}\right)(\omega R_P C_P)^n} \right) \right]^2 + \left[\omega L_S - \frac{R_P \sin\left(\frac{n\pi}{2}\right)(\omega R_P C_P)^n}{1 + (\omega R_P C_P)^{2n} + 2\cos\left(\frac{n\pi}{2}\right)(\omega R_P C_P)^n} \right]^2$$

where L_S represents the series inductance caused by the wires, R_S is the series resistance and R_P and C_P are the parallel resistance and capacitance.

These values are found by fitting the network impedance spectrum without converting to Z_{NS-J}. If the spectrum was converted to Z_{NS-J}, the series resistance would represent the nanosheet while the parallel resistance and capacitance would represent the junction. Suppl. Fig. 27b shows the inductance, L_S, extracted from the fits for each wire length. The inductance caused by the wiring is < 10^{-7} H for lengths below 15 cm. Suppl. Fig. 27c shows the parallel capacitance as a function of wire length. The value is approximately constant indicating that for wire lengths up to 65 cm, the measurement of C_P is negligibly affected by stray capacitance between the wires. However, it is good practise to keep loose wires to a minimum.

Supplementary note 11. Measuring impedance: Contact resistance

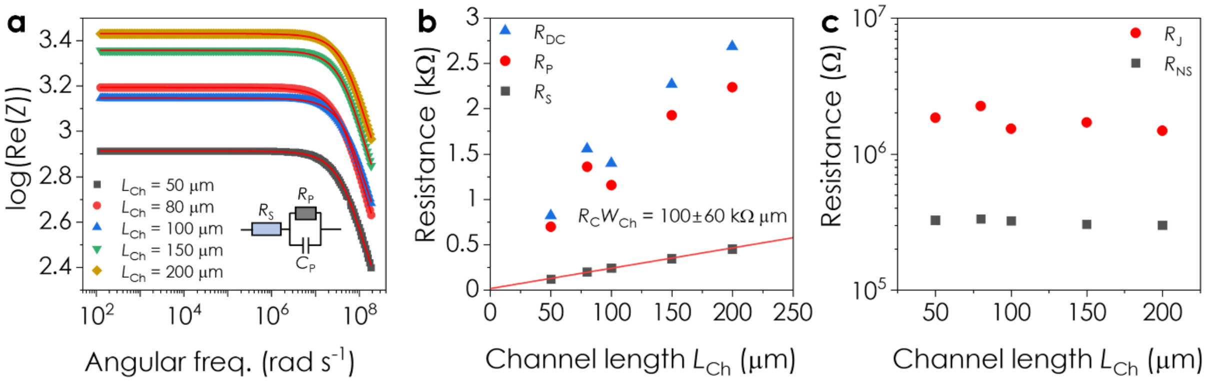

Supplementary figure 28: Measurement of contact resistance. (a) Real impedance spectra measured at different channel lengths for three-layer Langmuir-Schaefer deposited MoS_2 networks. Evaporated Ti:Au bottom electrodes were used with channel lengths of 50, 80, 100, 150, and 200 µm. Measured network impedance was not converted to $Z_{NS\text{-}J}$. Under these circumarances, fitting to a Randles circuit yields the series, R_S, and parallel resistrances, R_P, associated with the network rather than R_{NS} and R_J (Inset). **(b)** A plot of the total DC resistance (blue triangles), R_S (black squares) and R_P (red circles) versus channel length, L_{Ch}. Such a plot allows us to use the transfer length method (TLM) to determine the contact resistance, R_C, associated with the series resistance via $R_S = 2R_C + \rho_{NS} L_{Ch} / W_{Ch} t$, where ρ_{NS} is the nanosheet resisitvity, L_{Ch} is the channel length, and W_{Ch} and t are the channel width and network thickness. The nanosheet resistivity appears in this version of the TLM equation because the series resistance contains contributions from the nanosheet resistance whereas the parallel resistance contains contributions from the junction resistance. **(c)** Nanosheet and junction resistance, R_{NS} and R_J, plotted versus channel length.

Our method of using impedance spectroscopy to differentiate junction, R_J, and nanosheet resistance, R_{NS}, involves extracting the nanosheet resistance from the series resistance, R_S, which is observed at high frequency in the impedance spectrum. One important issue is that any contact resistance, R_C, associated with the interface between the nanosheet network and the electrodes would appear as a contribution to the series resistance. The total series resistance is the sum of the contact resistance and the resistance associated with the nanosheets (but not junctions) in the network (see Suppl. Fig. 28a, at high frequency where Z=R_S, the impedance of the capacitors becomes zero, shorting out all of the junction resistances). Conversely, the parallel resistance is the resistance associated with the junctions, but not nanosheets, in the network. Thus, it is important to prepare devices in a way that minimizes contact resistance and to show that contract resistance is not a significant contribution to the series resistance.

To test this, we measured impedance spectra on MoS_2 networks which had been contacted with five sets of electrodes, each with different channel length, L_{Ch}. The resultant impedance spectra are shown in Suppl. Fig. 28a. These spectra were fitted to a Randles circuit (Suppl. Fig. 28a, inset) from which the parallel, R_P, and series resistances, R_S, were extracted. These are plotted versus channel length in Suppl. Fig. 28b. The series resistance appears linear with a very small intercept (i.e. $2R_C$) relative to the R_S values. This shows that contact resistance is minimal in this system. The actual contact resistance is ≈ 100 kΩ µm, somewhat lower than the value of ≈ 400 kΩ µm reported by Chen et al. for similar networks[25]. We observe that the parallel resistance values are more scattered, which we attribute to spatial variations in network morphology. This results in larger values of junction resistance in some areas of a given thin film. In turn, this means that the DC resistances are also scattered. In principle, this means that impedance measurements of R_S are the best way to measure contact resistance in inhomogeneous systems. Finally, we found $Z_{NS\text{-}J}$ to be largely independent of channel length (Suppl. Fig. 28c), which allows us to rule out the effects of contact resistance. Both R_{NS} and R_J are seen to be indepenent of channel length in the range of L_{Ch} = 50 – 200 µm considered in this work (Suppl. Fig. 28c).

Supplementary note 12. Impedance: Fitting to equivalent circuits

Randles circuits

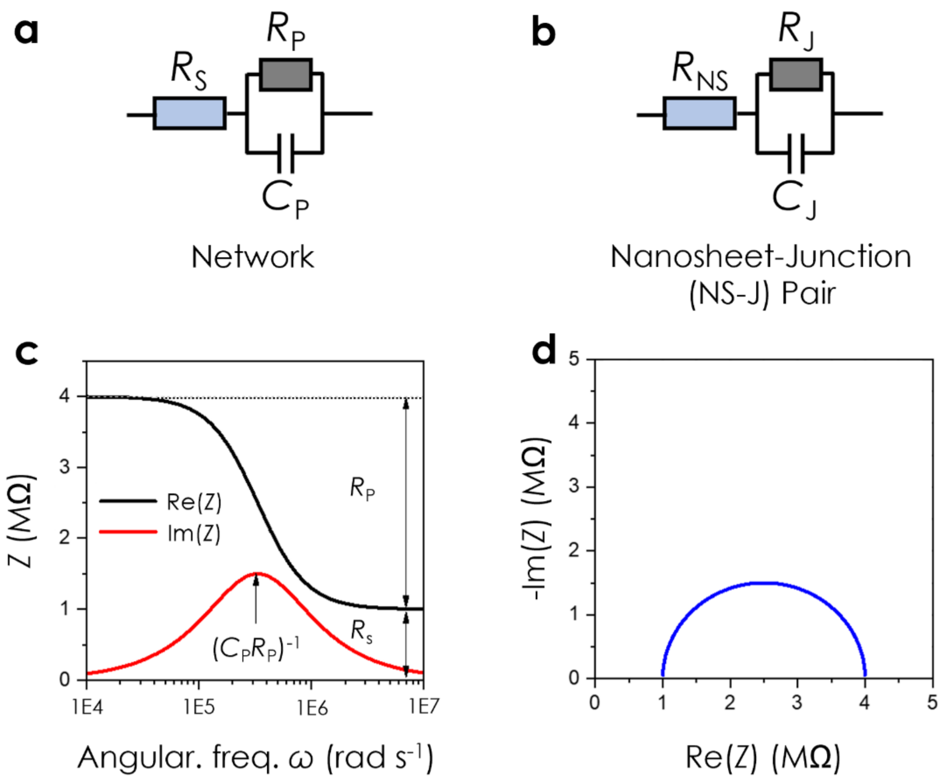

Supplementary figure 29: Randles equivalent circuits. Such circuits consist of a resistor in series, R_S, with a parallel resistor, R_P, and capacitor, C_P, combination. **(a)** This circuit is labelled in a general way that might represent any sample, for example a nanosheet network. **(b)** This circuit represents a nanosheet-junction pair. **(c)** Plots of real and imaginary impedance versus ω for a Randles circuit assuming $R_S = 1$ MΩ, $R_P = 3$ MΩ, $C_P = 1\times10^{-12}$ F. This diagram also shows how R_S and R_P (or R_{NS} and R_J for data representing a single NS-J pair) can be read off the graph. It also shows that the peak in Im(Z) occurs at $\omega_p = (C_p R_p)^{-1}$. **(d)** Cole-Cole plot showing imaginary, Im(Z), versus real, Re(Z), impedance for the same circuit.

In general, once one has measured real and imaginary impedance spectra, one can extract information by modelling the data as an appropriate equivalent circuit (Suppl. Fig. 29a-b). Usually, one fits the measured (here representing the network) impedance spectra using an equivalent circuit model such as that in Suppl. Fig. 29a. This equivalent circuit, often referred to as a Randles circuit in electrochemistry, is very commonly used in impedance spectroscopy. Its properties are summarised in Suppl. Fig. 29c-d.

However, a major advance of this work is the ability to convert the measured spectra (representing the network) to spectra representing the microscopic response, i.e. the response

of a single (average) nanosheet-junction (NS-J) pair. The NS-J impedance spectra can be analysed using the equations associated with the equivalent circuit shown in Suppl. Fig. 29b.

It is straightforward to show that the impedance associated with the circuit in Suppl. Fig. 29b has real and imaginary components given by[24]

$$\operatorname{Re} Z_{\text{NS-J}}(\omega) = R_{\text{NS}} + \frac{R_{\text{J}}}{1+\omega^2 R_{\text{J}}^2 C_{\text{J}}^2}$$

and

$$-\operatorname{Im} Z_{\text{NS-J}}(\omega) = \frac{\omega R_{\text{J}}^2 C_{\text{J}}}{1+\omega^2 R_{\text{J}}^2 C_{\text{J}}^2}$$

A specific example of the real and imaginary impedance of a Randles circuit plotted versus ω using these equations is shown in Suppl. Fig. 29c. These are sometimes called Bode plots. The hallmark of this circuit is that, when -Im($Z_{\text{NS-J}}$) is plotted versus Re($Z_{\text{NS-J}}$), the data forms a semicircle whose centre is on the Re($Z_{\text{NS-J}}$) axis at the coordinates (R_{NS}+R_{J}/2, 0). This -Im($Z_{\text{NS-J}}$) versus Re($Z_{\text{NS-J}}$) data is described by

$$[\operatorname{Im} Z_{\text{NS-J}}(\omega)]^2 = \left[R_{\text{J}} + R_{\text{NS}} - \operatorname{Re} Z_{\text{NS-J}}(\omega)\right]\left[\operatorname{Re} Z_{\text{NS-J}}(\omega) - R_{\text{NS}}\right]$$

which, it can be shown, is the equation of circle centred at (R_{NS}+R_{J}/2, 0). Such a graph is usually referred as a Nyquist or Cole-Cole plot. An example is shown in Suppl. Fig. 29d.

Circuits with distributions of C_J and R_J

Real networks consist of many nanosheet-junction pairs connected in series and in parallel (Suppl. Fig. 22). However, for a circuit like that shown in Suppl. Fig. 30 where all resistances and capacitances are the same, the overall impedance is just directly proportional to the impedance of any one RC circuit element (see above). This means the impedance spectra of such a circuit should be well described by the equations given above, slightly modified by some multiplicative factor as described in Supplementary note 6.

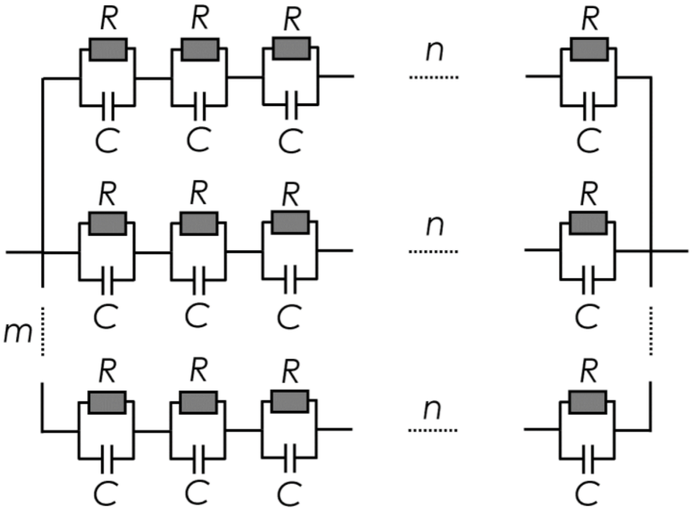

Supplementary figure 30: A network of identical parallel and series resistor, *R*, and capacitor, *C*, elements. In this representation, there are *m* parallel current paths, each containing *n* RC elements in series.

However, if the various circuit elements in this circuit have different values of *R* and *C*, the overall circuit will have impedance spectra that are somewhat broadened with respect to those of the individual circuit elements. Such spectra can be described by replacing the capacitors in Suppl. Fig. 29a-b with a non-ideal capacitive component known as a constant phase element.

Constant phase elements

It is quite common, particularly in electrochemistry, to find Nyquist plots which yield a depressed semicircle (i.e. one where the centre is below the Re(*Z*) axis), rather than the expected semicircle centred on-axis. The depressed nature of the semicircle is usually attributed to inhomogeneities somewhere in the system. Specifically, these inhomogeneities relate to the presence of a broad distribution of the resistances and capacitances of the individual circuit elements representing, in our case the individual nanosheet-junction pairs. Empirically, such depressed (i.e. broadened) data is usually fitted by replacing the capacitor in the circuit in Suppl. Fig. 30 by a constant phase element (CPE). A CPE is a generalised capacitor, originally hypothesised to empirically deal with the depressed semicircles observed in real data. The impedance of a CPE is usually expressed as

$$Z_{\mathrm{CPE}} = \frac{1}{Y_0 (i\omega)^n}$$

where Y_0 and n are constants. When the CPE is in parallel with a resistor, R, the capacitance associated with the CPE is given by $C=(Y_0R)^{1/n}/R$. This means the impedance of the combined parallel CPE-resistance (the so-called Zarc circuit) can be written as

$$Z_{\text{CPE-R}}=\frac{R}{1+(i\omega RC)^n}$$

If the capacitor in Suppl. Fig. 29b is replaced by a CPE with equivalent capacitance, C_J, the circuit shown in Suppl. Fig. 31 is obtained.

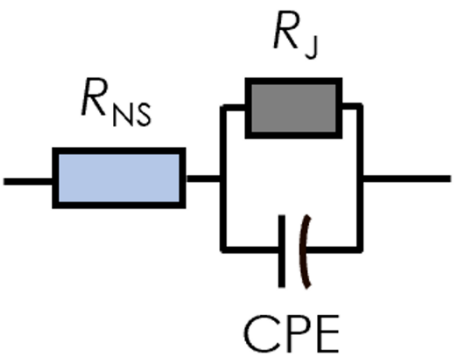

Supplementary figure 31: **A Zarc equivalent circuit.** For a nanosheet-junction pair this comprises a series resistor, R_{NS}, and a parallel combination of a resistor, R_J, and a constant phase element (CPE) in place of an ideal capacitor.

The impedance associated with this circuit is[26]

$$Z_{\text{NS-J}}=R_{\text{NS}}+\frac{R_{\text{J}}}{1+(i\omega R_{\text{J}}C_{\text{J}})^n}$$

This equation has real and imaginary components[26]

$$\operatorname{Re}Z_{\text{NS-J}}(\omega)=R_{\text{NS}}+\frac{R_{\text{J}}\left[1+(\omega R_{\text{J}}C_{\text{J}})^n\cos(n\pi/2)\right]}{1+2(\omega R_{\text{J}}C_{\text{J}})^n\cos(n\pi/2)+(\omega R_{\text{J}}C_{\text{J}})^{2n}} \quad \text{Equation S8}$$

and

$$-\operatorname{Im}Z_{\text{NS-J}}(\omega)=\frac{R_{\text{J}}(\omega R_{\text{J}}C_{\text{J}})^n\sin(n\pi/2)}{1+2(\omega R_{\text{J}}C_{\text{J}})^n\cos(n\pi/2)+(\omega R_{\text{J}}C_{\text{J}})^{2n}} \quad \text{Equation S9}$$

such that data in a Cole-Cole plot is described by

$$-\operatorname{Im} Z_{\text{NS-J}}(\omega) = \left[\left(\frac{R_{\text{J}}/2}{\sin(n\pi/2)}\right)^2 - \left(\operatorname{Re} Z_{\text{NS-J}}(\omega) - \left(R_{\text{NS}} + R_{\text{J}}/2\right)\right)^2\right]^{1/2} - \frac{R_{\text{J}}}{2}\frac{\cos(n\pi/2)}{\sin(n\pi/2)}$$

When $n = 1$, these equations simplify into the equations given above for the Randles circuit. For inhomogeneous systems such as nanosheet networks, these three equations can be used to fit graphs of Re($Z_{\text{NS-J}}$) vs. ω, -Im($Z_{\text{NS-J}}$) vs. ω and -Im($Z_{\text{NS-J}}$) vs. Re($Z_{\text{NS-J}}$) returning values of R_{NS}, R_{J}, C_{J} and n as fit parameters.

Physical interpretation of the constant phase element

While the physical interpretation of the CPE has been much debated, it is generally accepted that the presence of a CPE is usually required in the equivalent circuit when disorder is present. Particular attention has focused on systems where the equivalent circuit might best be described as a series linear array of N parallel RC elements such as that shown in Suppl. Fig. 32 (see Boukamp[27]).

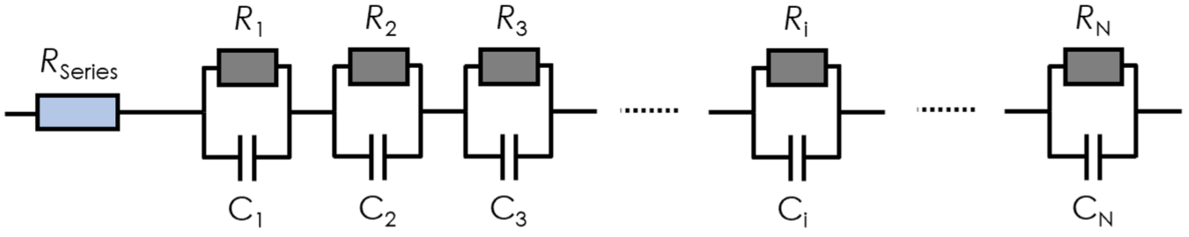

Supplementary figure 32: A linear array of resistor-capacitor elements in series with a series resistance.

The impedance of such a system is given by

$$Z = R_{\text{Series}} + \sum_{i=1}^{N} \frac{R_{\text{i}}}{1 + i\omega R_{\text{i}} C_{\text{i}}} = R_{\text{Series}} + \sum_{i=1}^{N} \frac{R_{\text{i}}}{1 + i\omega \tau_{\text{i}}}$$

where $\tau_{\text{i}} = R_{\text{i}} C_{\text{i}}$. If N is large, this sum can be converted to a distribution assuming the τ values follow the distribution $\gamma(\tau)$

$$Z = R_{\text{Series}} + R_{\text{P}} \int_0^{\infty} \frac{\gamma(\tau) d\tau}{1 + i\omega\tau}$$

where R_P is the polarisation resistance. In practice, it is often more useful to express the distribution as $G(\tau) = \tau \cdot \gamma(\tau)$ where $\int_{-\infty}^{\infty} G(\tau) d \ln\tau = 1$ such that[27]

$$Z = R_{\text{Series}} + R_{\text{P}} \int_{0}^{\infty} \frac{G(\tau) d \ln\tau}{1 + i\omega\tau}$$

When the product of resistance and capacitance for an individual RC unit in the array, $\tau_{\text{i}} = R_{\text{i}} C_{\text{i}}$, follows a log-normal distribution over the array as a whole, then the impedance response of such a circuit is the same as that of the circuit in Suppl. Fig. 31. Thus, the requirement to use a CPE rather than a capacitor in an equivalent circuit comes from the fact that the impedance spectra of some real systems reflect the average over many parallel RC combinations rather than a single RC circuit. Boukamp[27] has shown that the exponent, *n*, in the equation for the impedance of a CPE is a measure of the width of the distribution of R_iC_i values in the circuit in Suppl. Fig. 32. In this case, the distribution, *G*, follows a relatively simple equation

$$G(R_{\text{J}}C_{\text{J}}) = \frac{\sin(n\pi)}{2\pi\left(\cosh\left[n \ln\left(\langle R_{\text{J}}C_{\text{J}}\rangle / R_{\text{J}}C_{\text{J}}\right)\right] + \cos(n\pi)\right)}$$

As *n* decreases from 1, the distribution width increases strongly as shown below in Suppl. Fig. 33.

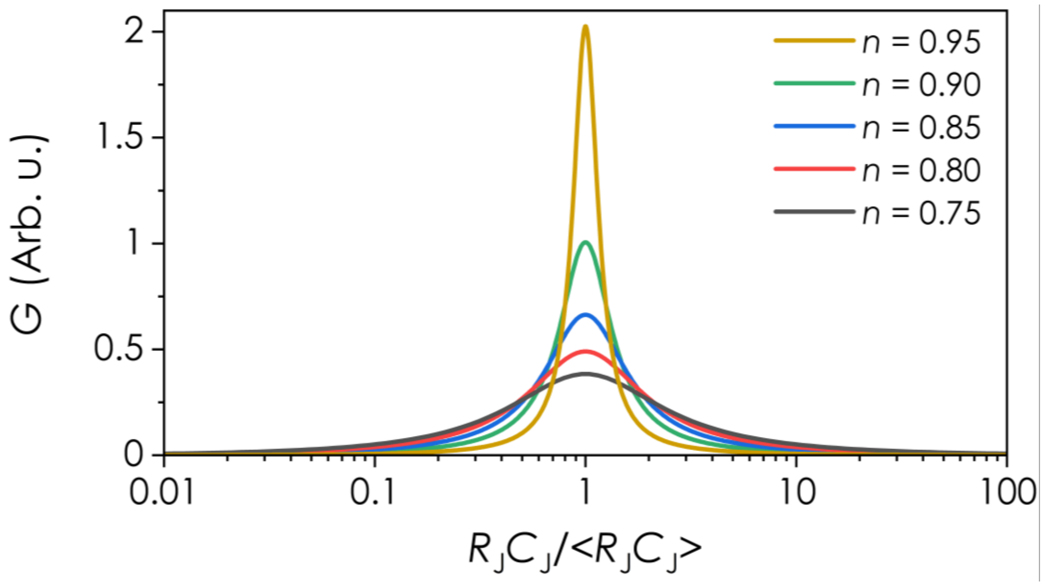

Supplementary figure 33: Theoretical distributions of R_JC_J within the network plotted for different values of *n*.

Supplementary note 13. Impedance: Fitting methodologies

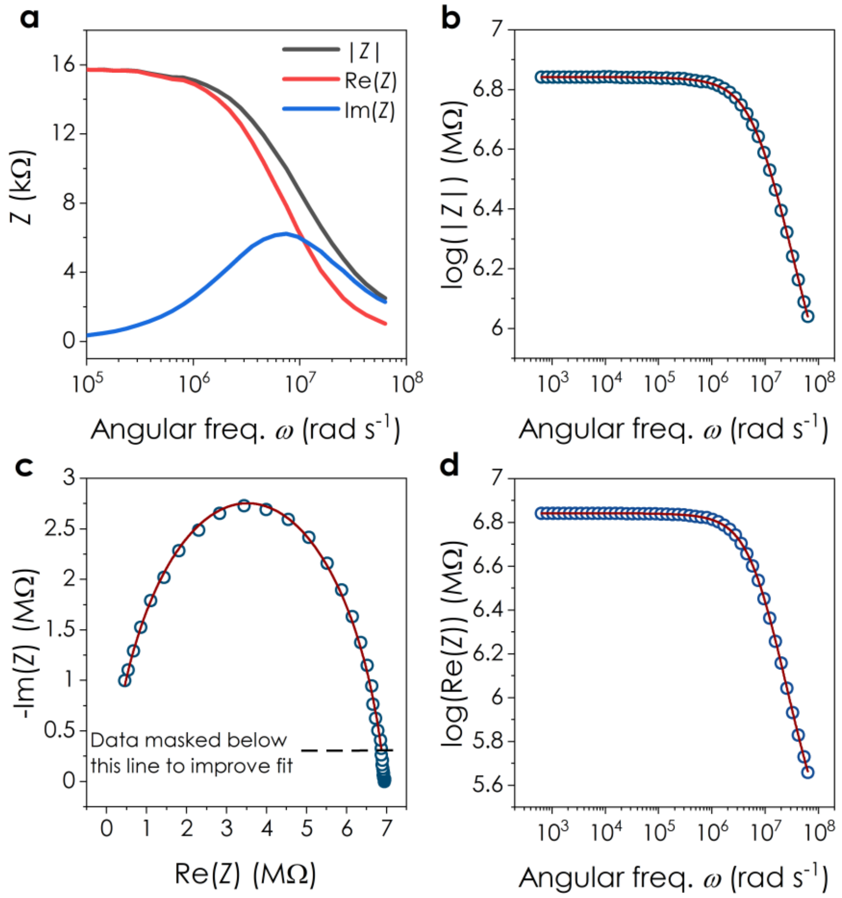

Supplementary figure 34: Fitting various spectra to extract R_{NS} from a two-layer MoS_2 sample. (a) Plots of |Z|, Re(Z) and -Im(Z) as a function of angular frequency, ω. **(b)** Bode plot of |Z| vs. ω. **(c)** Cole-Cole plot of -Im(Z) vs. Re(Z). **(d)** Bode plot of Re(Z) vs. ω. Here **(a)** represents network data, while **(b-d)** are converted to NS-J.

There are several methods for fitting impedance spectra to extract the value of R_J and R_{NS} which is the characteristic nanosheet resistance for a given network. This value can be extracted from the Bode plot (either of |Z| or Re(Z) vs. angular frequency, ω) or the Cole-Cole plot (-Im(Z) vs Re(Z)), however, these fits are not all equally accurate. Suppl. Fig. 34a shows |Z|, Re(Z) and -Im(Z) plotted for a two-layer MoS_2 sample. The |Z| spectrum is dominated by Re(Z) at low frequencies and by -Im(Z) at intermediate frequencies, as expected for a Randles circuit. If R_S is therefore extracted from a fit of such a |Z| spectrum, this will result in an overestimation of the high frequency plateau given the cut-off at the maximum measurable frequency (30 MHz) and, in some cases, may not find the high frequency plateau at all.

Suppl. Fig 34b-d shows the Bode plot of |Z| vs. ω, the Cole-Cole plot of -Im(Z) vs. Re(Z), and the Bode plot of Re(Z) vs. ω, respectively. Note that Suppl. Fig. 34b&d have the y-

axis plotted as the log of the impedance, which is done to facilitate fitting over multiple orders of magnitude. Table S2 shows the fit parameters from each graph where R_J and R_{NS} have their usual meaning and R^2 is the coefficient of determination.

	R_J (Ω)	R_{NS} (Ω)	R^2
$\|Z\|$	6.6×10^6 ± 1.5×10^4	3.4×10^5 ± 0.14×10^5	0.999
Cole-Cole	6.9×10^6 ± 2.5×10^4	9.2×10^4 ± 1.9×10^4	0.997
Re(Z)	6.9×10^6 ± 8.9×10^3	9.7×10^4 ± 0.5×10^4	0.999

Table S2: Fit parameters for the various plotting methods to extract R_J and R_{NS} from impedance spectra. The values for the junction resistance, R_J, and nanosheet resistance, R_{NS}, were extracted by fitting the Bode plots of $|Z|$ and Re(Z) and the Cole-Cole plot of -Im(Z) vs. Re(Z) in Suppl. Fig. 34.

The values of R_J for each method are reasonably close together although when R_J is extracted from $|Z|$ (Suppl. Fig. 34b), it has a slightly lower value than the other two methods because the value for R_{NS} is overestimated due to the dominance of Im(Z) at the high frequency limit. Fitting the Cole-Cole (Suppl. Fig. 34c) plot gives values for R_{NS} that are similar, however, to improve the accuracy of a fit on such a graph, the low frequency data points on the right-hand-side must be masked off to the point where the curvature begins. As a result, the fit on the Cole-Cole has a higher error in R_{NS} and a slightly lower R^2 value. We therefore use fits from Re(Z) (Suppl. Fig. 34d) in this work to extract R_J and R_{NS} from the impedance data.

Supplementary note 14. Room-temperature impedance of MoS_2

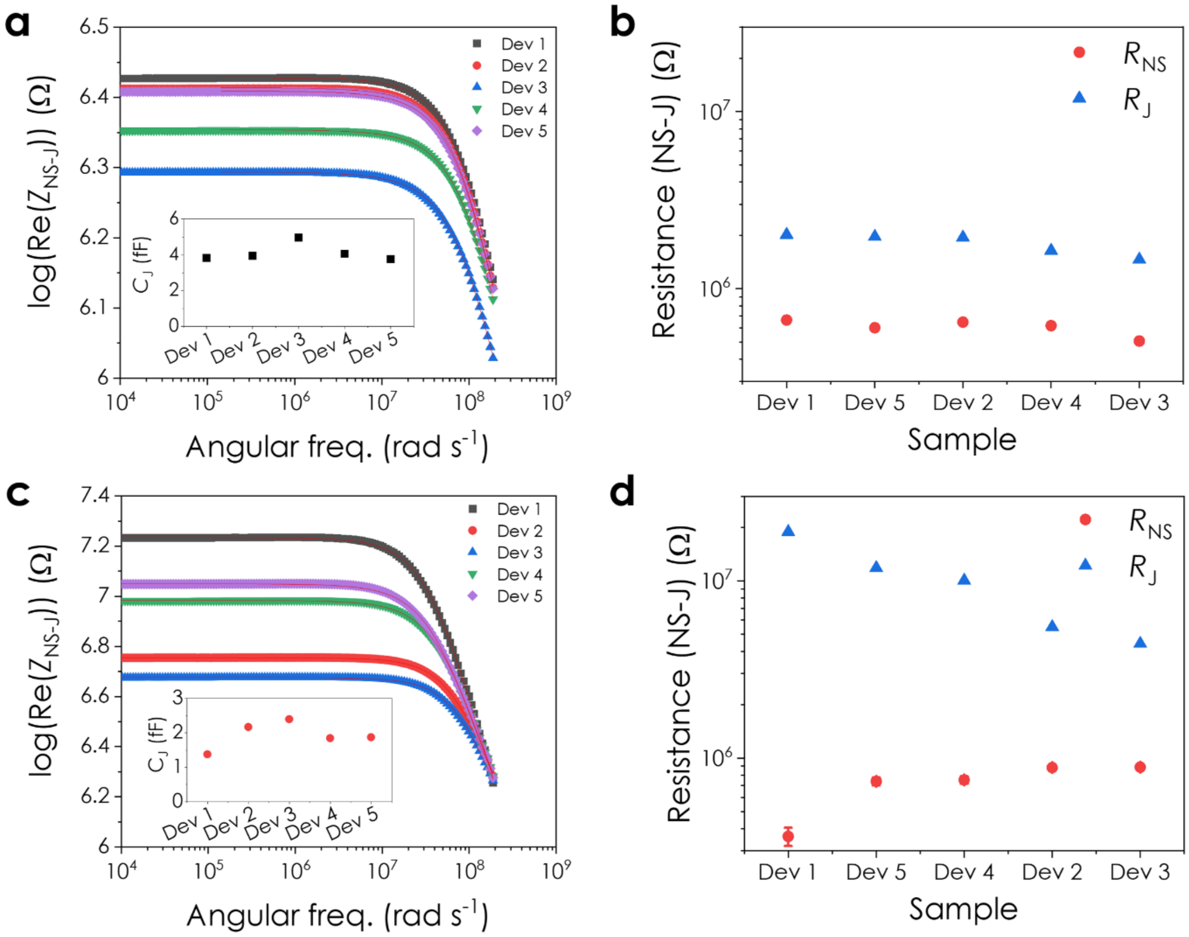

Supplementary figure 35: Variations across a given network. (a) Impedance spectra of five devices on a given electrochemically exfoliated MoS_2 network on the same substrate. Here the mean network thickness was t = 15 nm while the channel dimensions were L_{Ch} = 50 µm and W_{Ch} = 19.4 mm. The nanosheets were 3.3 nm thick on average. Junction capacitance, C_J, is given in the inset. **(b)** Nanosheet and junction resistances extracted from the data in **(a)**. **(c-d)** Equivalent data for a nominally identical, but poorer quality network. The uncertainties in (R_{NS}, R_J) in **(b)** and **(d)** are ± the errors in the fits to the Re(Z_{NS-J}) spectra in **(a)** and **(c)** respectively.

We have measured impedance spectra on multiple networks fabricated from electrochemically exfoliated MoS_2 (see also Suppl. Fig. 28). All networks were made using Langmuir-Schaefer deposition. Each network typically has five sets of electrodes and so five devices. The devices are spaced in different locations over a roughly 1 cm^2 area. Suppl. Fig. 35 shows impedance data for two different networks where the values of R_{NS} obtained are similar in both cases. This is expected as the nanosheets making up the networks were created under the same conditions. In Suppl. Fig. 35b, we find the R_J values to be consistent across devices. This implies that this network is spatially uniform with local morphology that does not vary significantly throughout

the network. As a result, the spatial variation in the local mean junction resistance is low. However, the R_J data in Suppl. Fig. 35d is different. There is a much larger variation over the five devices implying that this particular network is much less spatially uniform than the one in Suppl. Fig. 35b. This implies that our deposition method still needs to be perfected.

Suppl. Fig. 36 shows an example of another MoS_2 network where the devices show very different R_{NS}/R_J values. Comparing Device 1 (Suppl. Fig. 36a) to Device 5 (Suppl. Fig. 36b), we find a far higher value of R_{NS}/R_J in Device 5, much larger than any other device in the network. Optical microscopy identified an extremely large nanosheet spanning the channel and bridging the electrodes in this device. This nanosheet allowed a locally high current to pass through it without experiencing any junctions which results in a lower junction resistance.

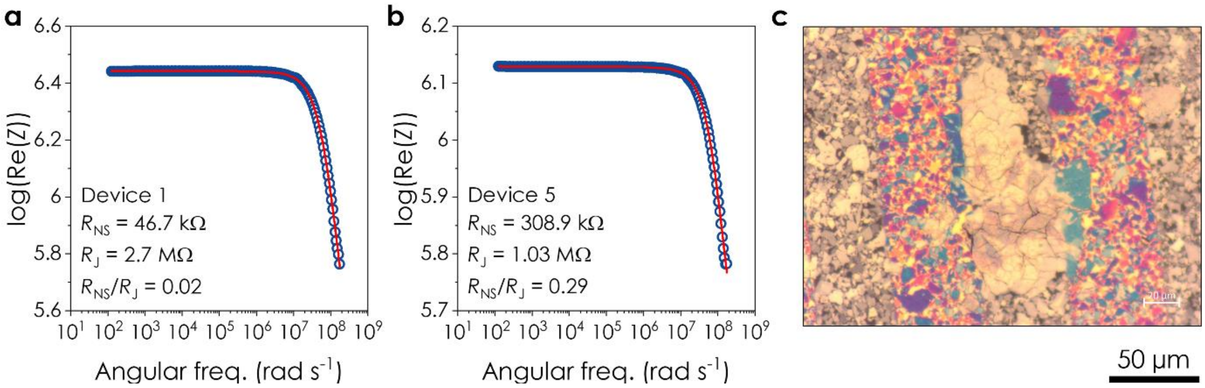

Supplementary figure 36: Effect of a single nanosheet bridging the electrodes. (a) The Re(Z) spectrum of the first set of electrodes where fitting yields R_{NS}/R_J = 0.02, indicating the network in this region is dominated by the junction resistance. **(b)** The Re(Z) spectrum from the fifth set of electrodes where fitting yields R_{NS}/R_J = 0.29 indicating the network in this region is far less junction-dominated. The reason for this is shown in **(c)** where a single large nanosheet can be seen bridging the gap between the electrodes. The distance between the first and fifth set of electrodes is approximately 5 mm.

Supplementary note 15. Extracting nanosheet resistivity directly from the network impedance

The circuit diagrams shown in Suppl. Figs. 29a-b show that that both the network as a whole and the NS-J pair can be described by Randles circuits. This means that we can consider certain frequency limits. As the frequency approaches zero, the impedance of the capacitor approaches infinity while as the frequency approaches infinity, the impedance of the capacitor approaches zero[24]. This means

$$\begin{aligned}
&\lim(\mathrm{Re}(Z_{\mathrm{Net}}))_{\omega\to 0} = R_{\mathrm{P}} + R_{\mathrm{S}} \\
&\lim(\mathrm{Re}(Z_{\mathrm{Net}}))_{\omega\to \infty} = R_{\mathrm{S}} \\
&\lim(\mathrm{Re}(Z_{\text{NS-J}}))_{\omega\to 0} = R_{\mathrm{J}} + R_{\mathrm{NS}} \\
&\lim(\mathrm{Re}(Z_{\text{NS-J}}))_{\omega\to \infty} = R_{\mathrm{NS}}
\end{aligned}$$

Keeping in mind the equation linking $Z_{\text{NS-J}}$ to Z_{Net} as

$$Z_{\text{NS-J}} = Z_{\mathrm{Net}} \frac{A_{\mathrm{Net}}}{L_{\mathrm{Ch}}} \frac{(1-P_{\mathrm{Net}})}{2t_{\mathrm{NS}}\kappa_{\mathrm{Net}}} \left[1 + \frac{2}{n_{\mathrm{NS}} t_{\mathrm{NS}} l_{\mathrm{NS}}^2}\right]^{-1}$$

We can write

$$R_{\mathrm{NS}} = (\mathrm{Re}(Z_{\text{NS-J}}))_{\omega\to\infty} \approx \frac{A_{\mathrm{Net}}(1-P_{\mathrm{Net}})}{2L_{\mathrm{Ch}} t_{\mathrm{NS}}\kappa_{\mathrm{Net}}} \left[1 + \frac{2}{n_{\mathrm{NS}} t_{\mathrm{NS}} l_{\mathrm{NS}}^2}\right]^{-1} (\mathrm{Re}(Z_{\mathrm{Net}}))_{\omega\to\infty}$$

And

$$R_{\mathrm{J}} + R_{\mathrm{NS}} = (\mathrm{Re}(Z_{\text{NS-J}}))_{\omega\to 0} \approx \frac{A_{\mathrm{Net}}(1-P_{\mathrm{Net}})}{2L_{\mathrm{Ch}} t_{\mathrm{NS}}\kappa_{\mathrm{Net}}} \left[1 + \frac{2}{n_{\mathrm{NS}} t_{\mathrm{NS}} l_{\mathrm{NS}}^2}\right]^{-1} (\mathrm{Re}(Z_{\mathrm{Net}}))_{\omega\to 0}$$

Also

$$\rho_{\mathrm{NS}} = R_{\mathrm{NS}} \times 2t_{\mathrm{NS}} \approx \frac{A_{\mathrm{Net}}(1-P_{\mathrm{Net}})}{L_{\mathrm{Ch}}\kappa_{\mathrm{Net}}} (\mathrm{Re}(Z_{\mathrm{Net}}))_{\omega\to\infty}$$

and

$$\rho_{\mathrm{Net}} = \frac{A_{\mathrm{Net}}}{L_{\mathrm{Net}}} (Z_{\mathrm{Net}})_{\omega\to 0}$$

These equations allow us to extract both nanosheet and junction resistances as well as network and nanosheet resistivity directly from network impedance spectra (so long as the high frequency plateau is clearly visible).

Supplementary note 16. Terahertz spectroscopy

Since all standard (not including the impedance technique demonstrated in this work) electrical measurements on networks are limited by junction resistance, it is not straightforward to use them to extract nanosheet parameters such as nanosheet mobility. However, it would be advantageous to measure properties of the individual nanosheets in order to compare them to those values extracted from impedance spectroscopy. To achieve this we use terahertz spectroscopy[28, 29].

Suppl. Fig. 37 shows the results of OPTP measurements on a one-LS-layer thick network of electrochemically exfoliated MoS_2 nanosheets. The observed mobility signal $S(\tau)=\Phi_e(\tau)\mu_e+\Phi_h(\tau)\mu_h$ represents the quantum-yield-weighted sum of electron and hole mobilities, averaged over the frequencies in the THz waveform at time τ after photoexcitation (see the first equation in the THz Spectroscopy section in the Methods, main text). The samples were photoexcited with a photon energy of 3.1 eV and an absorbed photon fluence of 2.7×10^{12} photons cm^{-2}, which was found to be sufficiently low to avoid higher-order recombination of electrons and holes. Photoexcitation at 3.1 eV leads to formation of electron-hole pairs with initial excess energy of 1.8 eV above the indirect band gap of MoS_2, which is 1.3 eV[30].

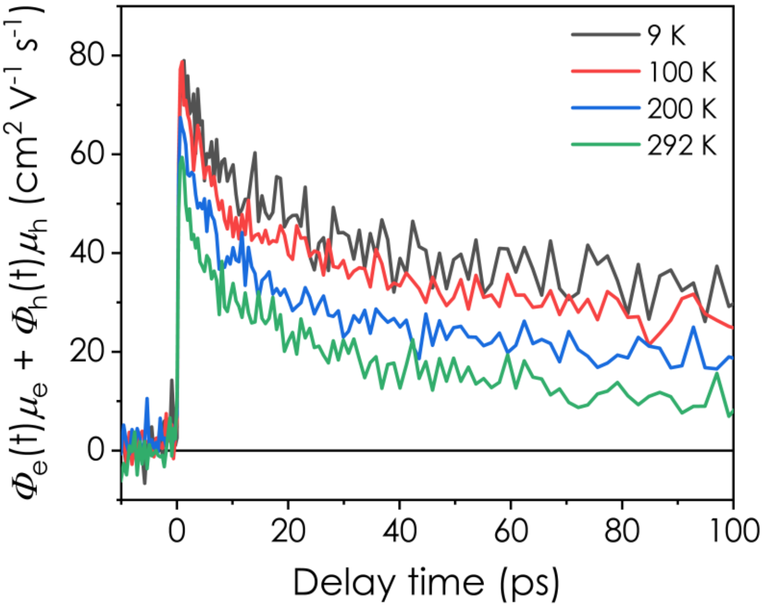

Supplementary figure 37: Temperature dependent decay kinetics of the observed mobility signal obtained from OPTP measurements on MoS_2. Plot of the quantum-yield-weighted sum of electron and hole mobilities, averaged over the frequencies in the THz waveform at time τ after photoexcitation.

The initially hot charges lose their excess energy via phonon emission and can subsequently decay by recombination to neutral excitons or trapping at defects. Note that excitons and

trapped charges do not contribute to $S(\tau)$. As discussed previously[19], after 5 ps the observed mobility can be attributed to relaxed charges at the band edges. At τ = 5 ps, the observed mobility at a temperature of 292 K is 40 ± 2 cm^2 V^{-1} s^{-1} (Suppl. Fig. 37). The mobility increases as the temperature goes down and reaches a value of 70 ± 2 cm^2 V^{-1} s^{-1} at 9 K. The decay of charges during time becomes faster with an increasing temperature. This suggests thermally activated trapping or electron-hole recombination.

Suppl. Fig. 38 shows the frequency-dependent quantum-yield-weighted sum of the electron and hole mobilities at different temperatures, obtained from TRTS measurements at τ = 5 ps. At a frequency of 1 THz, the observed mobilities are 40 ± 2 cm^2 V^{-1} s^{-1} and 70 ± 2 cm^2 V^{-1} s^{-1} at 292 K and 9 K, respectively. The mobilities at 1 THz are similar to those averaged over frequency, as obtained from OPTP measurements.

This can be understood by considering the amplitude of the THz field is at a maximum at 1 THz and in addition the mobility does not vary strongly with frequency, see Suppl. Fig. 38. However, note that the mobility decreases somewhat below 0.8 THz. Such behaviour can be due to charge scattering at barriers, which is more important at lower frequency (see Suppl. Fig. 38)[31, 32]. The decrease in the observed mobility with temperature must then be due to a dominating effect of enhanced electron-phonon scattering at higher temperature. The combination of scattering at barriers and phonons then results in the thermal deactivation of the mobility with temperature.

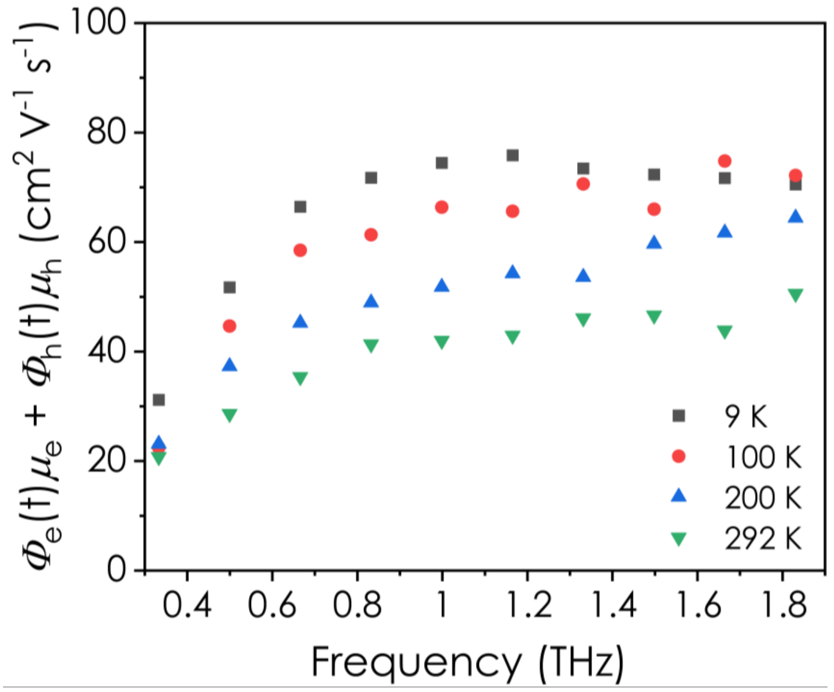

Supplementary figure 38: Frequency dependence of the observed mobility for different temperatures. The frequency-dependent quantum-yield-weighted sum of the electron and hole mobilities at different temperatures were obtained from TRTS measurements at 5 ps after photoexcitation of the sample.

Supplementary note 17. Transistor measurements on an individual nanosheet

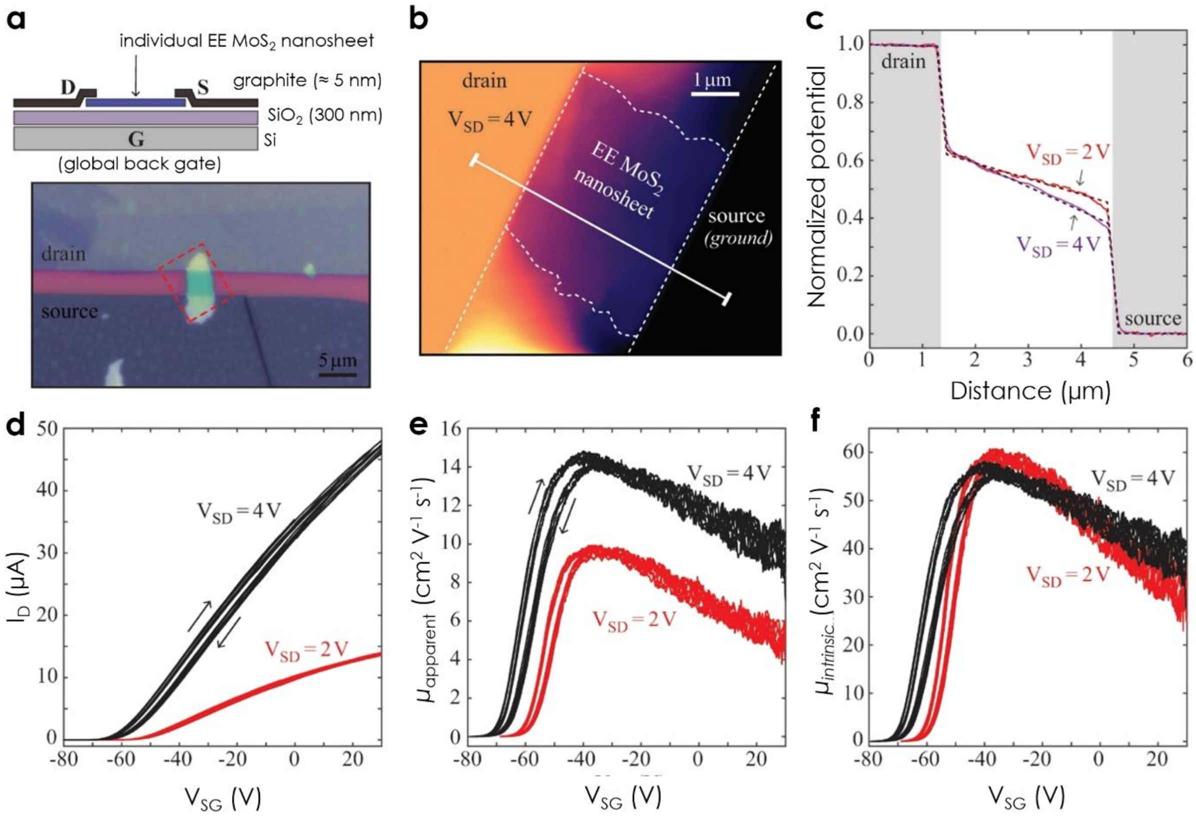

Supplementary figure 39: Field effect mobility of individual EE MoS_2 nanosheets. (a) Cross-sectional schematic (top) and an optical microscopy image (bottom) of an individual nanosheet device. EE MoS_2 nanosheets were dispersed on a SiO_2 chip and selected individual sheets were contacted by two graphite electrodes, transferred via PDMS stamps. **(b)** In-operando KPFM potential map (z-scale 7 V) of the device area marked by the dashed red rectangle in **(a)**. Dashed white lines in **(b)** mark the edges of the electrodes and the nanosheet. **(c)** Potential drop cross-sections (solid line in **(b)**) across the individual nanosheet device for two applied source-drain voltages, V_{SD}. The potential values are normalized to the applied bias and fitted by linear drops. Fitted values reveal a 16.5 % potential drop across the channel for V_{SD} = 2 V, and 25.2 % for V_{SD} = 4 V. High barriers at both source and drain electrodes are associated with the residuals from the EE process that are trapped between the nanosheet and the electrodes. **(d)** Electrical output curves of the device, presenting five subsequent forward and backward sweeps (arrows indicate sweeping direction). Minor hysteresis can be related to the device annealing and the presence of the residuals from the EE process at the gate interface. **(e)** Apparent linear electron mobility, $\mu_{apparent}$, calculated from **(d)**. **(f)** Estimated intrinsic linear electron mobility, $\mu_{intrinsic}$, after the applied V_{SD} values were corrected in accordance with the measured potential drops in **(c)**.

Supplementary note 18. Impedance of networks of 2D materials beyond MoS_2

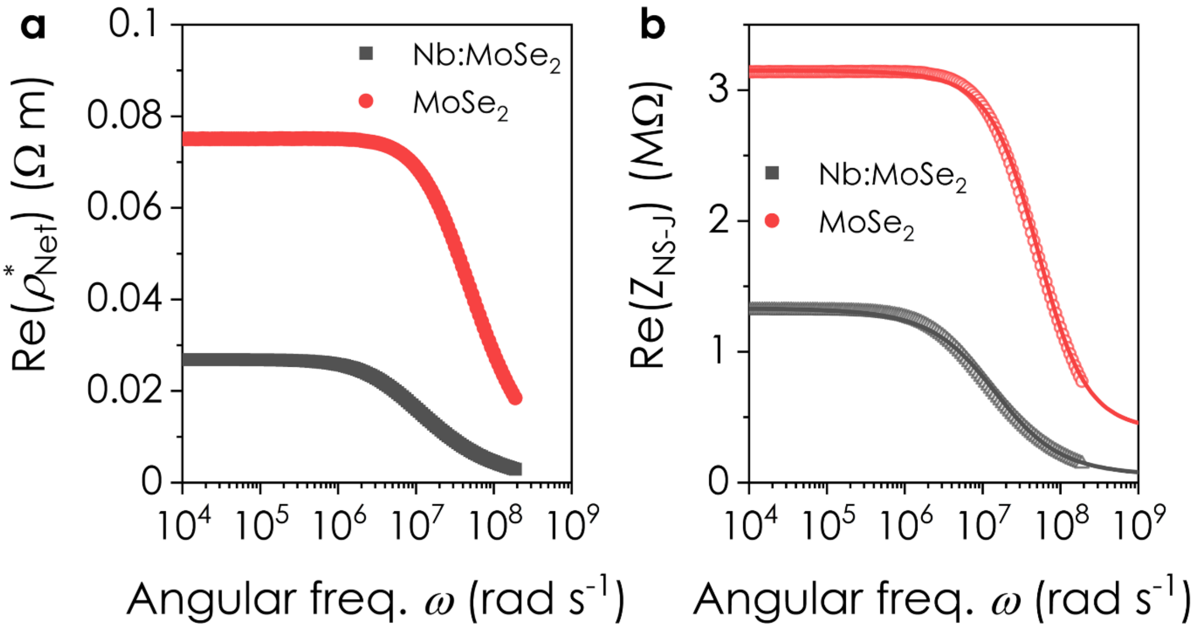

Supplementary figure 40: Impedance of LS-deposited networks of electrochemically exfoliated $MoSe_2$ and Nb-doped $MoSe_2$. (a) Real part of the complex network resistivity, Re(ρ^*_{Net}), plotted as a function of angular frequency, ω, for networks of EE $MoSe_2$ and niobium-doped EE $MoSe_2$ nanosheets. **(b)** Real component of the impedance of a nanosheet-junction pair, Re($Z_{NS\text{-}J}$), plotted vs. ω for both networks.

To demonstrate that impedance spectroscopy can be applied to networks of electrochemically exfoliated nanosheets beyond MoS_2, we fabricated 45 nm thick networks of electrochemically exfoliated $MoSe_2$ and Nb-doped $MoSe_2$ (~3% Nb, see Methods) using three layer-by-layer LS depositions in a manner very similar to that used to prepare the MoS_2 film used for impedance in the main manuscript. Suppl. Figs. 40a-b show the real part of the network resistivity, ρ^*_{Net}, and the converted $Z_{NS\text{-}J}$, respectively. We find spectra that clearly demonstrate a reduction in both R_{NS} and R_J upon doping of the nanosheets. A full analysis of these spectra, and those for other materials, will be published as future work.

Supplementary note 19. Fitting temperature-dependent impedance of MoS_2

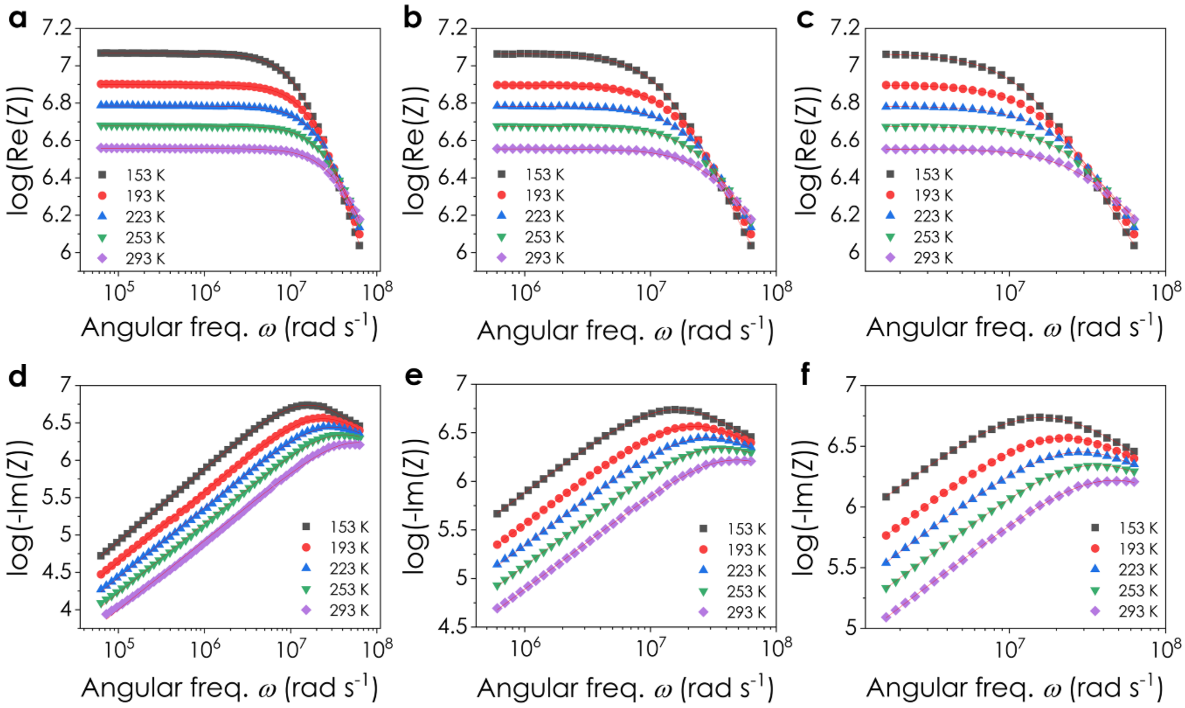

Supplementary figure 41: Fitting temperature-dependent Re(*Z*) and Im(*Z*) spectra for EE MoS_2. Examples of fits for **(a-c)** log(Re(*Z*)) and **(d-f)** log(-Im(*Z*)) vs. angular frequency, *ω*. In this case, fits were achieved using Eqns. S8 and S9 (representing inhomogeneous networks) and all fits are applied to the entire frequency range. This sample is the one reported in Fig. 5 of the main text.

When fitting impedance spectra, the frequency range across which the fit is performed can have significant influence on the quality of the fits. We tested the most appropriate fitting range by fitting data for Re(*Z*) and -Im(*Z*) versus *ω* in various ways. In all cases, the log of the impedance versus *ω* was plotted and fit using the log of Eqn. S8 and Eqn. S9, as using a log *y*-axis gives better fit quality for data whose *y*-range is greater than an order of magnitude. For both real and imaginary impedance data sets, fits were applied by fixing the high frequency limit at the upper experimental limit (63×10^6 rad s^{-1}). We then varied the low frequency limit with values decreasing from 3.8×10^6 rad s^{-1} to 63×10^3 rad s^{-1}. Examples of fits for the widest frequency range are shown in Suppl. Fig. 41. The fit parameters for each frequency range are plotted versus measurement temperature, *T*, in Suppl. Fig. 42 and Suppl. Fig. 43.

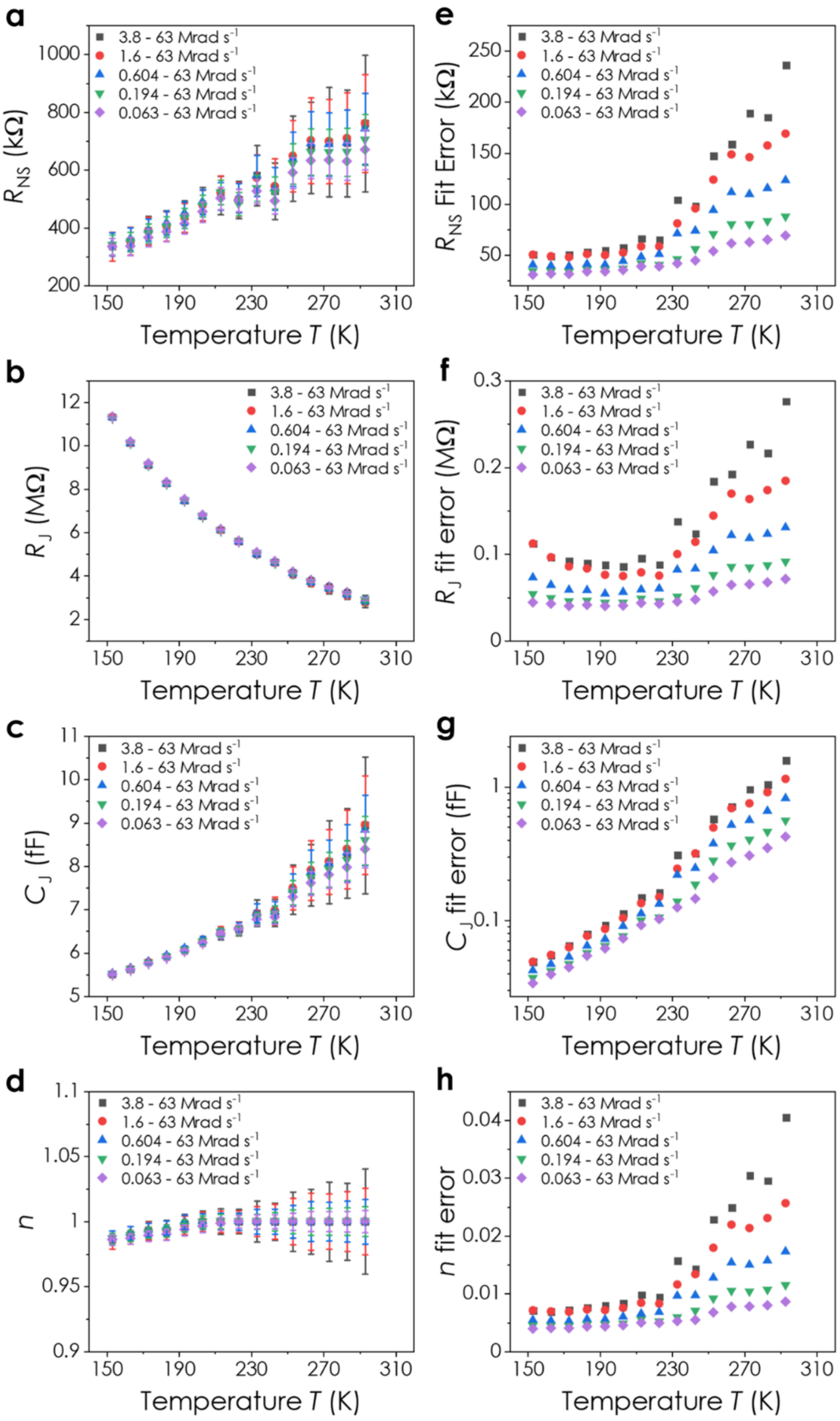

Supplementary figure 42: Fit parameters extracted from temperature-dependent Re(*Z*) spectra for EE MoS_2. (a-d) Fit parameters extracted from the log(Re(*Z*)) vs. ω fits (Suppl. Fig. 41) across various frequency ranges with **(e-h)** their accompanying fit errors.

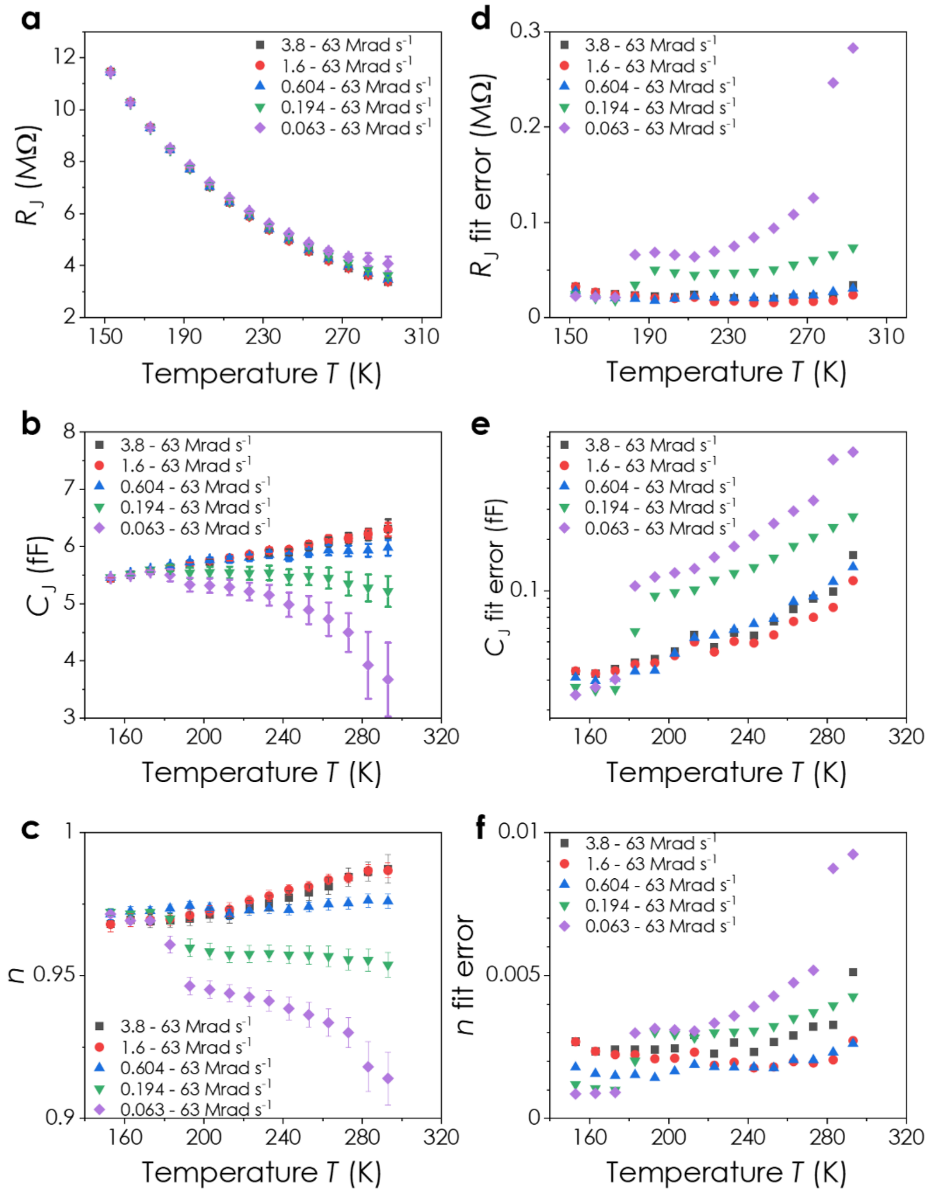

Supplementary figure 43: Fit parameters extracted from temperature-dependent Im(*Z*) spectra for EE MoS_2. (a-c) Fit parameters extracted from the log(-Im(*Z*)) vs. *ω* fits (Suppl. Fig. 41) across various frequency ranges with **(d-f)** their accompanying fit errors.

When fitting log(Re(*Z*)) vs. frequency data, it is important to fit a wide frequency range which includes the low frequency plateau, as demonstrated by Suppl. Fig. 42. With wide frequency ranges, the error in R_{NS}, R_J, C_J and *n* are all significantly reduced. Conversely, when fitting log(-Im(*Z*)) vs. frequency data, only a smaller frequency range that contains the peak is necessary. Suppl. Fig. 43 shows that at very wide frequency ranges, the errors in R_J, C_J and *n* increases meaning here the error is minimised when fit across a narrower frequency range. This is due to significant noise associated with the small values of Im(*Z*) found at very low frequencies.

Supplementary note 20. Measuring the inter-sheet junction area

The area of the inter-sheet junctions in a Langmuir-Schaefer deposited network of electrochemically exfoliated MoS_2 nanosheets was measured from SEM images (Suppl. Fig. 44). SEM measurements were performed on the network of electrochemically exfoliated MoS_2 nanosheets used for the temperature-dependent measurements (Fig. 5, main text). This network was formed using a single Langmuir-Schaefer deposition, resulting in the network being primarily comprised of a single layer of nanosheets. This, combined with the incomplete surface coverage shown in Suppl. Fig. 44a-b, provided enhanced topographic contrast in the SEM, allowing inter-sheet junctions to be easily imaged. Each junction in a SEM image was then identified and the overlap area was measured using *FIJI*[33], as shown in Suppl. Fig. 44b. The average junction area for the network was calculated from 807 measurements across the network surface and was found to be ~ 0.42 μm^2 (Suppl. Fig. 44c).

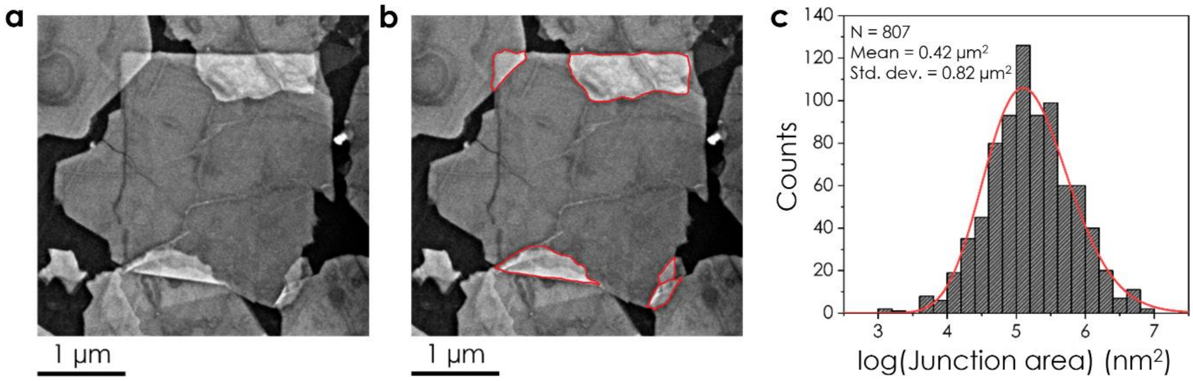

Supplementary figure 44: Measurement of nanosheet junction area. (a) High-resolution SEM image of a Langmuir-Schaefer deposited network of MoS_2 nanosheets produced using electrochemical exfoliation. The SE2 detector was used, which allowed inter-sheet junctions to be seen due to the topographic contrast. **(b)** The inter-sheet junctions were then outlined manually, and the area of each junction was measured. **(c)** Histogram of the measured inter-sheet junction areas across the sample.

Supplementary note 21. The effective permittivity of an MoS_2 network

Combining the equations in Supplementary note 15, we find that

$$R_J + R_{NS} = \frac{A_{Net}(1-P_{Net})}{2L_{Ch}t_{NS}\kappa_{Net}}\left[1+\frac{2}{n_{NS}t_{NS}l_{NS}^2}\right]^{-1}(R_P + R_S)$$

For simplicity we consider a situation where $R_J >> R_{NS}$ and n_{NS} is large. Then

$$R_J \approx \frac{A_{Net}(1-P_{Net})}{2L_{Ch}t_{NS}\kappa_{Net}}R_P$$

We also found above that $\omega_{peak} = \left(R_P C_P\right)^{-1} = \left(R_J C_J\right)^{-1}$ so $R_P C_P = R_J C_J$. We note that C_P is the effective capacitance of the nanosheet network as $C_P = R_J C_J / R_P$. This allows us to use the equation above to find

$$C_P = \frac{A_{Net}(1-P_{Net})}{2L_{Ch}t_{NS}\kappa_{Net}}C_J.$$

In the main text, we utilise $C_J = \varepsilon_0 A_J / l_J$. In addition, the effective network permittivity is defined via $C_P = \varepsilon_0 \varepsilon_{r,Net} A_{Net} / L_{Ch}$, so

$$\varepsilon_{r,Net} = \frac{(1-P_{Net})A_J}{2t_{NS}\kappa_{Net}l_J}$$

Taking $P_{Net} = 0.5$, $A_J = 0.5$ mm^2, $l_J = 0.6$ nm, $t_{NS} = 3.3$ nm, and $\kappa_{Net} = 1$, we find a value for $\varepsilon_{r,Net}$ of 6×10^4, which is extremely large.

To check this, we note that the C_P value found from fitting the Re(Z_{Net}) spectrum measured for the MoS_2 network analysed in Fig. 4 in the main text was 7×10^{-12} F. Here, the channel length and width were $L_{Ch} = 50$ µm and $W_{Net} = 19.4$ mm, while the network thickness was $t = 15$ nm. This implies a network permittivity of 14×10^4, in reasonable agreement with the model (any discrepancies are due to inaccuracies in our parameter estimation). Despite these very high permittivity values, we don't expect MoS_2 networks to make good dielectrics due to the expected large leakage currents since R_J is large but not infinite.

The equation above for the in-plane capacitance can also be obtained in a manner similar to the derivation of $Z_{\mathrm{Net}} = Z_{\mathrm{NS\text{-}J}} n/m$ at the end of Supplementary note 6. We assume each junction has a capacitance: $C_0 = \varepsilon_0 A_{\mathrm{J}} / l_{\mathrm{J}}$. Then, each conducting path is a chain of n junction capacitors in series, $n = \kappa_{\mathrm{Net}} L_{\mathrm{Ch}} / (l_{\mathrm{NS}} / 2)$, where κ_{Net} accounts for the fact that the path is probably not straight and the factor of 2 is to account for the fact that on average the flakes are separated by half the nanosheet length. There are m such paths in parallel, $m = A_{Net}(l_{NS} t_{NS} / (1-P_{Net}))^{-1}$ where the factor $(1-P_{\mathrm{Net}})$ accounts for the free space between nanosheets. Combining gives

$$C_{\mathrm{Net}} = C_{\mathrm{J}} \frac{m}{n} = \varepsilon_0 \frac{A_{\mathrm{J}}}{l_{\mathrm{J}}} \frac{A_{\mathrm{Net}} / \left[l_{\mathrm{NS}} t_{\mathrm{NS}} / (1-P_{\mathrm{Net}}) \right]}{\kappa_{\mathrm{Net}} L_{\mathrm{Ch}} / (l_{\mathrm{NS}} / 2)} = \varepsilon_0 \frac{A_{\mathrm{J}}}{l_{\mathrm{J}}} \frac{A_{\mathrm{Net}}}{L_{\mathrm{Ch}}} \frac{(1-P_{\mathrm{Net}})}{2\kappa_{\mathrm{Net}} t_{\mathrm{NS}}} = \varepsilon_{\mathrm{r,Net}} \varepsilon_0 \frac{A_{\mathrm{Net}}}{L_{\mathrm{Ch}}}$$

$$\varepsilon_{\mathrm{r,Net}}^{\mathrm{IP}} = \frac{A_{\mathrm{J}}}{l_{\mathrm{J}}} \frac{(1-P_{\mathrm{Net}})}{2\kappa_{\mathrm{Net}} t_{\mathrm{NS}}}$$

Here we explicitly add the label IP to signify that it is the in-plane permittivity.

This method is useful as it allows us to estimate the out-of-plane network permittivity in the same way. We can consider a chain of p capacitors spanning the thickness of the network, t. There are q of these chains distributed over the area ($W_{\mathrm{Ch}} \times L_{\mathrm{Ch}}$) of the film. We estimate $q \approx L_{\mathrm{ch}} W_{\mathrm{ch}} / l_{\mathrm{NS}}^2$ and $p = (1-P_{\mathrm{Net}}) t / t_{\mathrm{NS}}$. Following the same methodology, we find

$$\varepsilon_{\mathrm{r,Net}}^{\mathrm{OOP}} = \frac{A_{\mathrm{J}}}{l_{\mathrm{J}}} \frac{t_{\mathrm{NS}}}{l_{\mathrm{NS}}^2} \frac{1}{(1-P_{\mathrm{Net}})}$$

Supplementary note 22. Temperature-dependent transport in nanosheet networks

Literature

Several papers have now performed temperature-dependent conductivity measurements on nanosheet networks of solution-processed MoS_2, two using liquid-phase exfoliated MoS_2[19, 34] and two using electrochemically exfoliated MoS_2[35], including this work. The distinction between exfoliation methods is important to note as both types of nanosheet show a change in transport behaviour in the range of 160-200 K; liquid-phase exfoliated nanosheets transition from tunnelling to hopping as temperature is increased[19, 34], while electrochemically exfoliated nanosheets transition from variable-range hopping to an activated behaviour[35,This work].

The difference here is caused by the dominating effect of the inter-sheet junctions which is strongly mediated by nanosheet aspect ratio[5], where electrochemically exfoliated nanosheets tend to have a much higher aspect ratio than liquid-phase exfoliated nanosheets. The relative rigidity of the low aspect-ratio, liquid-phase exfoliated nanosheets prevents intimate contact at the junctions, thus creating a large junction resistance (Fig. 3, main text). The resistance associated with hopping depends exponentially on both the distance between hopping sites and temperature meaning that a wide inter-sheet distance combined with a low temperature produces an excessively high inter-sheet hopping resistance, which rules it out below a critical temperature. Below this temperature, transport can only occur by inter-sheet tunnelling which is a comparatively inefficient transport process that suppresses the conductivity of the network to $< 10^{-9}$ S m^{-1} [19, 34].

By contrast, the large area and conformal junctions between the electrochemically exfoliated nanosheets result in a very low junction resistance (Fig. 4, main text) which allows a more efficient hopping transport to occur across the junctions at all temperatures. Variable-range hopping at lower temperatures (< 200 K) is commonly seen in measurements of individual nanosheets[36, 37, 38] as the energetic disorder of the nanosheet means a low energy site will always be accessible within a reasonable range of the carrier. To the best of the authors knowledge, intra-sheet tunnelling transport in MoS_2 has not been seen at low temperatures.

Activated hopping

Miller-Abrahams theory shows that the resistance associated with hopping between two sites under an applied electric field is given by[39]

$$R_{\mathrm{ij}} \approx \frac{k_{\mathrm{B}}T}{e^2\nu_0}\exp\left(\frac{2r_{\mathrm{ij}}}{a}\right)\exp\left(\frac{\varepsilon_{\mathrm{ij}}}{k_{\mathrm{B}}T}\right) \qquad \text{Equation S10}$$

Where r_{ij} and e_{ij} are the spatial and energy distances between sites while a is the localisation length and ν_0 is an attempt frequency. In the simplest case, we can apply this to describe the junction resistance data at high temperature where activated behaviour is observed

$$R_{\mathrm{J}} \approx \frac{k_{\mathrm{B}}T}{e^2\nu_0}\exp\left(\frac{2l_{\mathrm{J}}}{a}\right)\exp\left(\frac{E_{\mathrm{a}}}{k_{\mathrm{B}}T}\right) = R_{\mathrm{J},0}\exp\left(\frac{2l_{\mathrm{J}}}{a}\right)\exp\left(\frac{E_{\mathrm{a}}}{k_{\mathrm{B}}T}\right)$$

Where l_{J} is the inter-sheet separation (distance across the junction) and E_{a} is the activation energy. The equation describes the R_{J} versus T data for T >235 K reasonably well with $R_{\mathrm{J},0}\exp(2l_{\mathrm{J}}/a)$ = 282 kΩ and E_{a} = 58 meV. Approximating $\nu_0 = 10^{13}$ Hz and taking l_{J} = 0.6 nm and a = 0.7 nm (see below) predicts $R_{\mathrm{J},0}\exp(2l_{\mathrm{J}}/a)$ = 90 kΩ, lower than the measured value. The discrepancy might be due to the limitations of the model (prefactors are always to be treated with caution in these models), or inaccuracies in the values of l_{J} and a used.

At temperatures below 235 K, our data is consistent with 3D variable range hopping (3D-VRH). Activated hopping is often observed to transition to variable range hopping at low temperatures[40, 41]. The reason for this is that the reduced temperature limits the number of states within a reasonable energy range that a carrier can hop to. This forces the carrier to hop to more distant states that are closer in energy. The standard derivation of variable range hopping[39] considers hopping between nearby localised states, usually in the band tail or within the bandgap.

However, when considering the junction resistance between two nanosheets, we must consider hopping across the van der Waals gap between the sheets (Suppl. Fig. 45a). This defines a minimum hopping distance. In addition, because our data implies band-like transport within the nanosheets, we must consider hopping from states near the conduction band edge of one nanosheet to the conduction band of another. These differences require modification to the standard VRH derivation which we outline here by modifying the derivation given ref. [39].

Following Eqn. S10, the junction resistance can be written as

$$R_{\mathrm{J}} \approx R_{\mathrm{J},0}\exp(\alpha)$$

where

$$\alpha = \frac{2r_{ij}}{a} + \frac{\varepsilon_{ij}}{k_B T}$$

Variable range hopping is the situation where the resistance is minimised by considering the factors which minimise α.

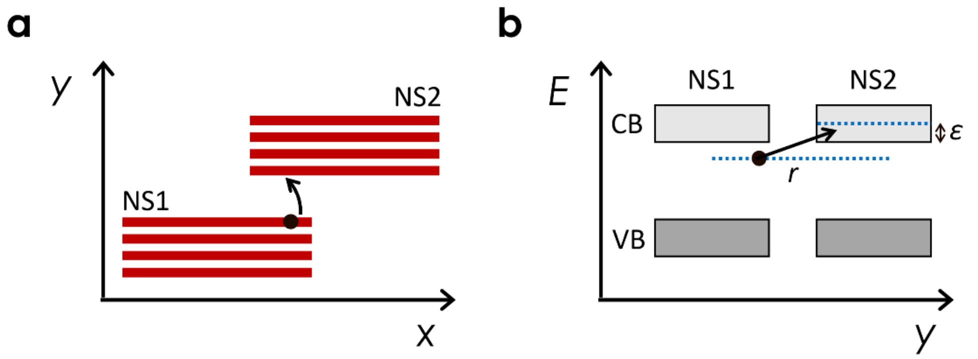

Supplementary figure 45: Electron hopping between nanosheets. (a) Schematic showing two multilayer nanosheets (NS1 and NS2) with a planar junction and an electron (black circle) hopping from one to another (NS1 to NS2)**. (b)** Energy band diagram showing the conduction (CB) and valence bands (VB) of NS1 and NS2. Here, an electron is shown hopping from a localised state just below the bottom of the conduction band of NS1 to the conduction band of NS2. The hopping distance is *r*. We assume the electron can hop into states within an energy ε of the bottom of the CB of NS2.

The hopping scheme we envisage is shown in Suppl. Fig. 45a-b where an electron hops from a localized state just below the conduction band-edge of one nanosheet to the conduction band of the other nanosheet. We assume it can hop into a range of states within an energy, ε, of the bottom of the conduction band of the second nanosheet. The minimum hopping distance is the inter-sheet separation (l_J) with some hops being slightly longer than that depending on the final state. The number of states (per unit volume) accessible within the conduction band of the second nanosheet is $N = g\varepsilon$, where g is the density of states at the bottom of the conduction band. The average spatial separation between these states is $d = (g\varepsilon)^{-1/3}$. Thus, we assume the hopping distance to be the sum of the inter-sheet separation and the distance to the nearest state $r_{ij} = l_J + d = l_J + (g\varepsilon)^{-1/3}$, while we approximate $\varepsilon_{ij} = \varepsilon$. This yields

$$\alpha = \frac{2}{a}(l_J + (g\varepsilon)^{-1/3}) + \frac{\varepsilon}{k_B T}$$

The minimum resistance is achieved when α is minimized. We identify the conditions for this via differentiation:

$$\frac{d\alpha}{d\varepsilon} = \frac{-2}{3a}(g)^{-1/3}(\varepsilon)^{-4/3} + \frac{1}{k_{\mathrm{B}}T} = 0$$

Rearranging yields the most likely hopping energy

$$\varepsilon_{\mathrm{o}} = \left[\left(\frac{3a}{2k_{\mathrm{B}}T}\right)^{3} g\right]^{-1/4}$$

Combining this value with $r_{\mathrm{ij}} = l_{\mathrm{J}} + (g\varepsilon)^{-1/3}$, yields the most probable hopping distance

$$r_0 = l_{\mathrm{J}} + \left[\frac{3a}{2k_{\mathrm{B}}Tg}\right]^{1/4}$$

Next, we replace e_{ij} and r_{ij} in Eqn. S10 with their most probable values, ε_0 and r_0

$$R_{\mathrm{J}} \approx \frac{k_{\mathrm{B}}T}{e^2\nu_0}\exp\left(\frac{2}{a}l_{\mathrm{J}}\right)\exp\left(\left[\frac{76}{k_{\mathrm{B}}Tga^3}\right]^{1/4}\right)$$

yielding

$$R_{\mathrm{J}} \approx R_{\mathrm{J},0}\exp\left(\frac{2l_J}{a}\right)\exp\left(\left[\frac{T_0}{T}\right]^{1/4}\right)$$

With

$$T_0 = \frac{76}{k_{\mathrm{B}}ga^3}$$

These equations are almost identical to the standard 3D VRH equation. However, here, g represents the density of states (DOS) at the bottom of the conduction band (in the standard equation, it represents the DOS at the Fermi energy). We can approximate g within each monolayer using the equation for the density of states of a 2D electron gas

$$g_{\mathrm{2D}} = \frac{m}{\pi\hbar^2}$$

However, the nanosheets themselves are a few nanometers thick and are essentially quasi-3D. We can convert this 2D density of states to an effective 3D density of states by dividing through by the monolayer thickness, d_0

$$g \approx g_{3D} = \frac{g_{2D}}{d_0} = \frac{m}{\pi\hbar^2 d_0}$$

This equation gives values which are in reasonable agreement with more sophisticated calculations[42] and yields a final approximate expression for T_0

$$T_0 \approx \frac{76\pi\hbar^2 d_0}{k_B a^3 m}$$

This equation is different to the standard 3D-VRH expression for T_0 and we now use this equation to analyse our experimental data. Taking the fit value of $T_0 = 4.93\times10^5$ K and the effective mass known for MoS_2 ($m = 0.7m_e$) allows one to calculate the localisation length in MoS_2 to be $a \approx 0.7$ nm. This is similar to published data which varies between 0.2 and ≈ 3 nm[35, 36, 37, 43]. We can also compare this value to the Bohr radius in MoS_2 to which it should be similar to the localisation length[44]

$$a_B = \frac{4\pi\hbar^2 \varepsilon_r \varepsilon_0}{e^2 m}$$

Taking $\varepsilon_r = 6$ [45] and $m = 0.7m_e$ yields $a_B = 0.45$ nm which is very similar to the value obtained above. We can modify the equation for the most probable hopping distance using the approximation for g given above

$$r_0 = l_J + \left[\frac{3a\pi\hbar^2 d_0}{2mk_B T}\right]^{1/4}$$

Taking $d_0 = 0.6$ nm, this gives a value which is 1.7 nm larger than the inter-sheet distance at room temperature, leading to most probable hopping distance of 2.3 nm. Similarly, we modify the equation for the most probable hopping energy using the approximation for g given above

$$\varepsilon_o = \left[\left(\frac{3a}{2k_B T}\right)^3 \frac{m}{\pi\hbar^2 d_0}\right]^{-1/4}$$

This yields a most probable hopping energy which increases with temperature roughly as 1.5 k_BT, reaching 35 meV at 233 K, which is roughly where the behaviour transitions from VRH to activated. We note that this value is lower than the activation energy of 58 meV found in the activated region.

Discussion

It is worth noting that both the Miller-Abrahams and VRH equations describe hopping between localised states[39]. However, here we are considering a situation where the carriers undergo bandlike transport within the nanosheets, presumably via delocalised states, interspaced with hopping events between nanosheets. This means that on its journey, a carrier must transition between delocalised states (transport within the nanosheets) and localised states (during the inter-sheet transfer process) multiple times. Presumably this also applies in other systems, such as in networks of carbon nanotubes. We note that Schießl et al.[46] successfully modelled inter-tube hopping in nanotube networks using the Miller-Abrahams equation without considering the details of the transition between delocalised and localised states.

It is likely that our nanosheets have localised states below the band edge, perhaps due to the presence of S-vacancies[47] or other defects created during electrochemical exfoliation. Indeed, papers have shown hopping transport within individual nanosheets[35, 44, 48]. However, like other papers on electrochemically exfoliated MoS_2[11], we see high *n*-doping for reasons that are not fully understood. This doping pushes the Fermi energy up toward the conduction band edge, filling these trap states and allowing band-like transport to occur in the nanosheets themselves. Such band-like transport within nanosheets has also been seen in the literature by many authors[49, 50, 51] with various papers showing transitions from hopping to band-like transport as the carrier density is increased[44, 52].

To explain the presence of hopping between localised states, one possibility is that immediately prior to the inter-sheet hop, carriers get trapped in a localised state close to the junction. Such “transit” states might be associated with the nanosheet edge or be due to band bending[53] near the junction causing the Fermi energy to locally move into the band tail. From this localised state, the carriers hop, either directly into a delocalised state in the conduction band of the second nanosheet or to a similar localised “transit” state just below the mobility edge of the second nanosheet whence they can be rapidly-promoted to a delocalised state. Understanding the details of this process will be an important part of the future work following this project.

References

1. De S, Coleman JN. The effects of percolation in nanostructured transparent conductors. *MRS Bulletin* **36**, 774-781 (2011).

2. De S, King PJ, Lyons PE, Khan U, Coleman JN. Size Effects and the Problem with Percolation in Nanostructured Transparent Conductors. *ACS Nano* **4**, 7064-7072 (2010).

3. Tjaden B, Brett DJL, Shearing PR. Tortuosity in electrochemical devices: a review of calculation approaches. *International Materials Reviews* **63**, 47-67 (2016).

4. Biccai S*, et al.* Negative Gauge Factor Piezoresistive Composites Based on Polymers Filled with MoS2 Nanosheets. *ACS Nano* **13**, 6845-6855 (2019).

5. Kelly AG, O'Suilleabhain D, Gabbett C, Coleman JN. The electrical conductivity of solution-processed nanosheet networks. *Nature Reviews Materials* **7**, 217-234 (2021).

6. Ponzoni A. The contributions of junctions and nanowires/nanotubes in conductive networks. *Applied Physics Letters* **114**, (2019).

7. O'Suilleabhain D, Vega-Mayoral V, Kelly AG, Harvey A, Coleman JN. Percolation Effects in Electrolytically Gated WS2/Graphene Nano:Nano Composites. *ACS Applied Materials & Interfaces* **11**, 8545-8555 (2019).

8. Tian R*, et al.* Quantifying the factors limiting rate performance in battery electrodes. *Nature Communications* **10**, (2019).

9. Gabbett C*, et al.* Quantitative analysis of printed nanostructured networks using high-resolution 3D FIB-SEM nanotomography. *Nature Communications* **15**, (2024).

10. Neilson J, Avery M, Derby B. Tiled Monolayer Films of 2D Molybdenum Disulphide Nanoflakes Assembled at Liquid/Liquid Interfaces. *ACS Applied Materials & Interfaces*, (2020).

11. Lin Z*, et al.* Solution-processable 2D semiconductors for high-performance large-area electronics. *Nature* **562**, 254-258 (2018).

12. Backes C*, et al.* Equipartition of Energy Defines the Size-Thickness Relationship in Liquid-Exfoliated Nanosheets. *ACS Nano* **13**, 7050-7061 (2019).

13. Forro C, Demko L, Weydert S, Voros J, Tybrandt K. Predictive Model for the Electrical Transport within Nanowire Networks. *ACS Nano* **12**, 11080-11087 (2018).

14. Backes C*, et al.* Spectroscopic metrics allow in situ measurement of mean size and thickness of liquid-exfoliated few-layer graphene nanosheets. *Nanoscale* **8**, 4311-4323 (2016).

15. Synnatschke K*, et al.* Length- and Thickness-Dependent Optical Response of Liquid-Exfoliated Transition Metal Dichalcogenides. *Chemistry of Materials* **31**, 10049-10062 (2019).

16. Carey T*, et al.* High-Mobility Flexible Transistors with Low-Temperature Solution-Processed Tungsten Dichalcogenides. *ACS Nano* **17**, 2912-2922 (2023).

17. Harvey A*, et al.* Non-resonant light scattering in dispersions of 2D nanosheets. *Nature Communications* **9**, (2018).

18. Backes C*, et al.* Edge and confinement effects allow *in situ* measurement of size and thickness of liquid-exfoliated nanosheets. *Nature Communications* **5**, (2014).

19. Kelly AG*, et al.* All-printed thin-film transistors from networks of liquid-exfoliated nanosheets. *Science* **356**, 69-73 (2017).

20. Klein CA, Straub WD. Carrier densities and mobilities in pyrolytic graphite. *Physical Review* **123**, 1581-& (1961).

21. Paton KR*, et al.* Scalable production of large quantities of defect-free few-layer graphene by shear exfoliation in liquids. *Nature Materials* **13**, 624-630 (2014).

22. Cancado LG*, et al.* Science and Metrology of defects in graphene using Raman Spectroscopy. *Carbon* **220**, (2024).

23. Backes C*, et al.* Production of Highly Monolayer Enriched Dispersions of Liquid-Exfoliated Nanosheets by Liquid Cascade Centrifugation. *ACS Nano* **10**, 1589-1601 (2016).

24. Lazanas AC, Prodromidis MI. Electrochemical Impedance Spectroscopy-A Tutorial. *ACS Measurement Science Au* **3**, 162-193 (2023).

25. Chen M*, et al.* Inkjet-Printed MoS2 Nanoplates on Flexible Substrates for High-Performance Field Effect Transistors and Gas Sensing Applications. *ACS Applied Nano Materials* **6**, 3236-3244 (2023).

26. Heil T, Jossen A. Continuous approximation of the ZARC element with passive components. *Measurement Science and Technology* **32**, (2021).

27. Boukamp BA. Distribution (function) of relaxation times, successor to complex nonlinear least squares analysis of electrochemical impedance spectroscopy? *Journal of Physics-Energy* **2**, (2020).

28. Lloyd-Hughes J, Jeon TI. A Review of the Terahertz Conductivity of Bulk and Nano-Materials. *Journal of Infrared Millimeter and Terahertz Waves* **33**, 871-925 (2012).

29. Spies JA*, et al.* Terahertz Spectroscopy of Emerging Materials. *Journal of Physical Chemistry C* **124**, 22335-22346 (2020).

30. Mak KF, Lee C, Hone J, Shan J, Heinz TF. Atomically Thin MoS2: A New Direct-Gap Semiconductor. *Physical Review Letters* **105**, (2010).

31. Grozema FC, Siebbeles LDA. Mechanism of charge transport in self-organizing organic materials. *International Reviews in Physical Chemistry* **27**, 87-138 (2008).

32. Ulbricht R, Hendry E, Shan J, Heinz TF, Bonn M. Carrier dynamics in semiconductors studied with time-resolved terahertz spectroscopy. *Reviews of Modern Physics* **83**, 543-586 (2011).

33. Schindelin J*, et al.* Fiji: an open-source platform for biological-image analysis. *Nature Methods* **9**, 676-682 (2012).

34. Ippolito S*, et al.* Unveiling Charge-Transport Mechanisms in Electronic Devices Based on Defect-Engineered MoS2 Covalent Networks. *Advanced Materials*, (2023).

35. Piatti E*, et al.* Charge transport mechanisms in inkjet-printed thin-film transistors based on two-dimensional materials. *Nature Electronics* **4**, 893-905 (2021).

36. Xue JH, Huang SY, Wang JY, Xu HQ. Mott variable-range hopping transport in a MoS2 nanoflake. *RSC Advances* **9**, 17885-17890 (2019).

37. Qiu H*, et al.* Hopping transport through defect-induced localized states in molybdenum disulphide. *Nature Communications* **4**, (2013).

38. Park T-E*, et al.* Hopping conduction in p-type MoS2 near the critical regime of the metal-insulator transition. *Applied Physics Letters* **107**, (2015).

39. Shlimak I. *Is Hopping a Science?: Selected Topics of Hopping Conductivity*. World Scientific Publishing (2015).

40. Rudra M, Tripathi HS, Dutta A, Sinha TP. Existence of nearest-neighbor and variable range hopping in Pr2ZnMnO6 oxygen-intercalated pseudocapacitor electrode. *Materials Chemistry and Physics* **258**, (2021).

41. Halim J, Moon EJ, Eklund P, Rosen J, Barsoum MW, Ouisse T. Variable range hopping and thermally activated transport in molybdenum-based MXenes. *Physical Review B* **98**, (2018).

42. Wang KC, *et al.* Control of interlayer physics in 2H transition metal dichalcogenides. *Journal of Applied Physics* **122**, (2017).

43. Kim JS, *et al.* Electrical Transport Properties of Polymorphic MoS2. *ACS Nano* **10**, 7500-7506 (2016).

44. Cheng J, *et al.* Temperature dependent carrier transport in few-layered MoS2: from hopping to band transport. *Journal of Physics D-Applied Physics* **55**, (2022).

45. Hou YP, Wang GR, Ma CF, Feng ZH, Chen YH, Filleter T. Quantification of the dielectric constant of MoS2 and WSe2 Nanosheets by electrostatic force microscopy. *Materials Characterization* **193**, (2022).

46. Schiess SP, *et al.* Modeling carrier density dependent charge transport in semiconducting carbon nanotube networks. *Physical Review Materials* **1**, (2017).

47. Zhao YF, *et al.* Electrical spectroscopy of defect states and their hybridization in monolayer MoS2. *Nature Communications* **14**, (2023).

48. Peng OG, *et al.* Metal-Contact-Induced Transition of Electrical Transport in Monolayer MoS2: From Thermally Activated to Variable-Range Hopping. *Advanced Electronic Materials* **5**, (2019).

49. Huo N, Yang Y, Wu Y-N, Zhang X-G, Pantelides ST, Konstantatos G. High carrier mobility in monolayer CVD-grown MoS2 through phonon suppression. *Nanoscale* **10**, 15071-15077 (2018).

50. Lin M-W, *et al.* Thickness-dependent charge transport in few-layer MoS2 field-effect transistors. *Nanotechnology* **27**, (2016).

51. Radisavljevic B, Kis A. Mobility engineering and a metal-insulator transition in monolayer MoS2. *Nature Materials* **12**, 815-820 (2013).

52. Rani V, *et al.* Carrier Induced Hopping to Band Conduction in Pentacene. *Scientific Reports* **9**, (2019).

53. Zhu Z, Kim J-S, Moody MJ, Lauhon LJ. Edge and Interface Resistances Create Distinct Trade-Offs When Optimizing the Microstructure of Printed van der Waals Thin-Film Transistors. *ACS Nano* **17**, 575-586 (2022).
